# Influence of axle length on the rate and mechanism of shuttling in rigid H-shaped [2]rotaxanes†
†Electronic supplementary information (ESI) available: Preparation and characterization of all precursors and [2]rotaxanes; experiments used to determine shuttling rates; X-ray diffraction experiments and computational details. CCDC 1563641–1563644. For ESI and crystallographic data in CIF or other electronic format see DOI: 10.1039/c7sc03736h


**DOI:** 10.1039/c7sc03736h

**Published:** 2017-09-25

**Authors:** Ghazale Gholami, Kelong Zhu, Giorgio Baggi, Eduardo Schott, Ximena Zarate, Stephen J. Loeb

**Affiliations:** a Department of Chemistry and Biochemistry , University of Windsor , Windsor , Ontario N9B 3P4 , Canada . Email: loeb@uwindsor.ca; b School of Chemistry , Sun Yat-Sen University , Guangzhou , 510275 , P. R. China . Email: zhukelong@mail.sysu.edu.cn; c Departamento de Química Inorgánica , Facultad de Química , Pontificia Universidad Católica de Chile , Avenida Vicuña Mackenna, 4860 , Santiago , Chile; d Instituto de Ciencias Químicas Aplicadas , Facultad de Ingeniería , Universidad Autónoma de Chile , Avenida Pedro de Valdivia 425 , Santiago , Chile

## Abstract

A series of [2]rotaxane molecular shuttles was prepared containing a dibenzo[24]crown-8 (**DB24C8**) wheel and a rigid H-shaped axle with varying track lengths between recognition sites; from 7.4 to 20.3 Å as defined by 1–4 phenyl rings or a naphthyl group. The rate of shuttling was measured by variable temperature ^1^H NMR spectroscopy for neutral compounds and EXSY experiments for dicationic species. The rates were found to be independent of the length of the axle, except when the distance between the two recognition sites might be short enough (*n* = 1) to allow the crown ether to simultaneously interact with both recognition sites providing a short-cut mechanism which could lower the energy barrier. This notion is supported by DFT calculations and solid-state characterization of model compounds that mimic possible transition states.

## Abstract 2

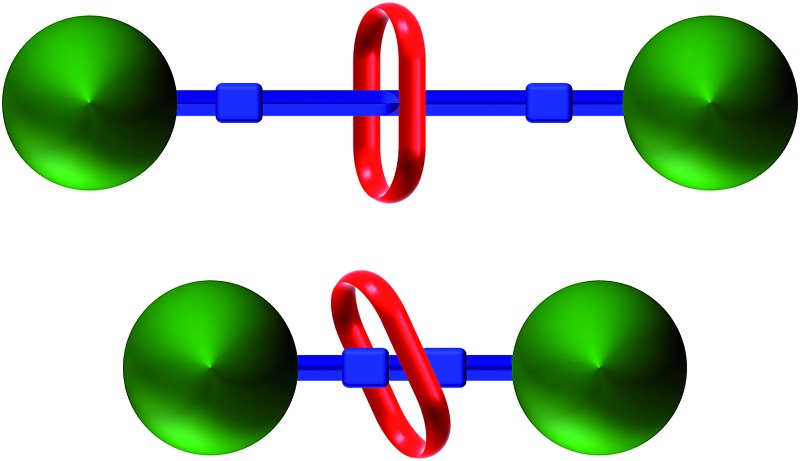
Shuttling rates for neutral and charged [2]rotaxanes with rigid axles varying in lengths from 7.4 to 20.3 Å were found to be independent of the length of the axle, except when the distance was short enough to allow the ring to interact with both recognition sites which provided a short-cut mechanism that significantly lowered the energy barrier.

## Introduction

One of the earliest classes of mechanically interlocked molecules (MIMs) to be investigated for dynamic motion was the molecular shuttle.^1^ The first such system reported by Stoddart in 1991, consisted of a tetracationic macrocycle that translocated between two identical electron-rich aromatic groups attached to a flexible polyether chain.^2^ The energy barrier for this to-and-fro motion was determined to be 13 kcal mol^–1^ in acetone solution. Since that pioneering work, many different structural versions of the molecular shuttle have been prepared and the corresponding energy barriers to translational motion measured.^3^

A number of studies have attempted to correlate the rate of shuttling to the structure of the molecular path along which the ring must travel.^4^ In particular, the conformational freedom of the track along which the ring shuttles, can have a significant effect on the barrier. For example, when the track is a flexible polyether or hydrocarbon chain folding can cause a significant increase to the shuttling barrier and clearly a very long track would require a non-linear trajectory with several different sized energy barriers.^5^ Studies have shown that folded conformations can impede shuttling by presenting steric barriers^6^ and flexibility can even change the mechanism by allowing the wheel to simultaneously interact with both initial and final recognition sites.^7^ Even answering a seemingly simple question such as – is there a relationship between the shuttling rate and the distance between the recognition sites? – can be difficult.

**Fig. 1:**
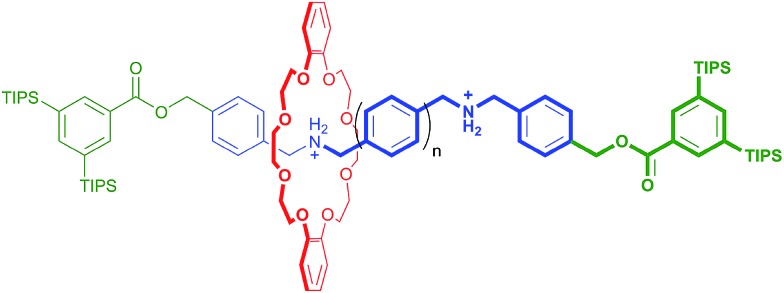
Hirose and co-worker's degenerate molecular shuttle with alkyl ammonium recognition sites and phenylene spacers (*n* = 1–4); ref. 8.

In an elegant study in 2014, Hirose and co-workers prepared a series of degenerate molecular shuttles with alkyl ammonium recognition sites and a rigid track comprised of phenyl rings; Fig. 1.^8^ By incorporating an increasing number of phenylene spacers in the axle (*n* = 1–4), they were able to extend the distance between the two ammonium N-atoms from 6.9 (*n* = 1) to 19.6 Å (*n* = 4). This eliminated the effects of conformational isomerism and it was concluded that “axle length does not affect switching dynamics in degenerate molecular shuttles with rigid spacers”.

During our investigations on incorporating molecular shuttles into solid-state materials by using rotaxanes as linkers for metal–organic frameworks (MOFs),^9^ we prepared a series of [2]rotaxane molecular shuttles with a rigid H-shaped axle.^10^ We rationalised that it should also be possible to extend this motif by adding an increasing number of phenyl rings, or equally rigid groups, to prepare a series of molecular shuttles with a fairly wide variation in shuttling track length. As in Hirose's work, these could then be used to more accurately probe the effect of track length – the distance between recognition sites – on the rate of shuttling without the previously encountered flexibility of the axle being an issue. These new elongated [2]rotaxanes would also be amenable to incorporation into solid-state materials.

This study corroborates Hirose's major tenant that axle length does not appreciably effect shuttling rate, but expands significantly upon their work as our system can be studied in both fast (neutral) and slow (dication) NMR rate regimes. We also add a cautionary tale about oversimplifying the mechanism of shuttling as we show it can be easily short circuited, even in a rigid molecular design. We report herein, the preparation of a series of five rigid, H-shaped [2]rotaxane molecular shuttles comprised of two equivalent benzimidazolium recognition sites, a track containing *n* = 1–4 phenyl rings or a naphthyl ring and a single dibenzo[24]crown-8 ether, **DB24C8** macrocycle. The distance between the N-atoms of the recognition sites varies between 7.4 and 20.3 Å and the rate of shuttling can be measured for benzimidazole and benzimidazolium forms.

## Results and discussion

### Preparation and characterisation of molecular shuttles

**Scheme 1:**
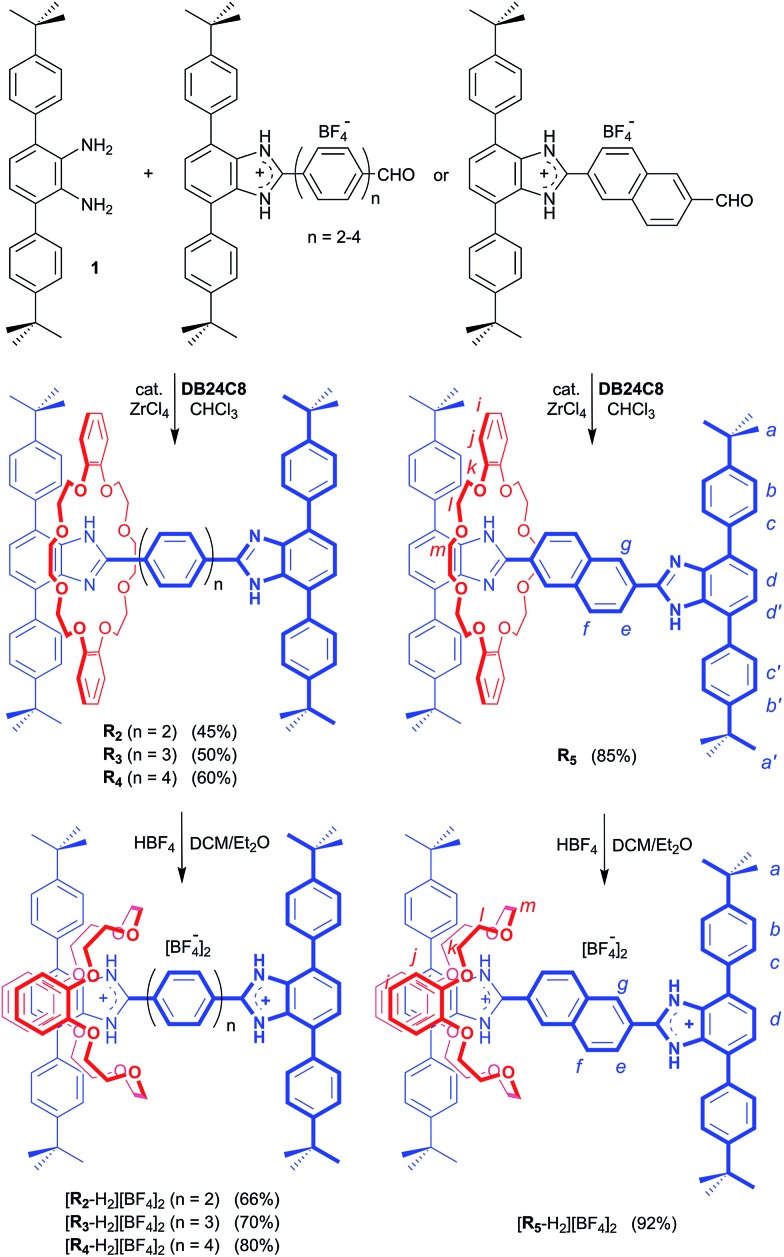
Preparation of rigid, H-shaped [2]rotaxane molecular shuttles containing 2, 3 and 4 phenyl rings or a naphthyl group along the track. Preparation of aldehyde precursors (*n* = 2–4, Np) are described in the ESI.† The *n* = 1 analogue was previously prepared as described in ref. 11. H-atom labels are for NMR spectral assignments shown in Fig. 2.

Preparation of the required aldehyde precursors with *n* = 2–4 and Np (naphthyl) spacers are described in the ESI.† Formation of the [2]rotaxanes was accomplished by condensation between the appropriate aldehyde with *n* = 2–4 or Np and diamine **1** in the presence of **DB24C8** utilising ZrCl_4_ as catalyst as outlined in Scheme 1. The neutral species **R_2_–R_5_** were isolated initially and then converted to the dicationic versions [**R_2_**-H_2_]^2+^ – [**R_5_**-H_2_]^2+^ by protonation with HBF_4_.

**Fig. 2:**
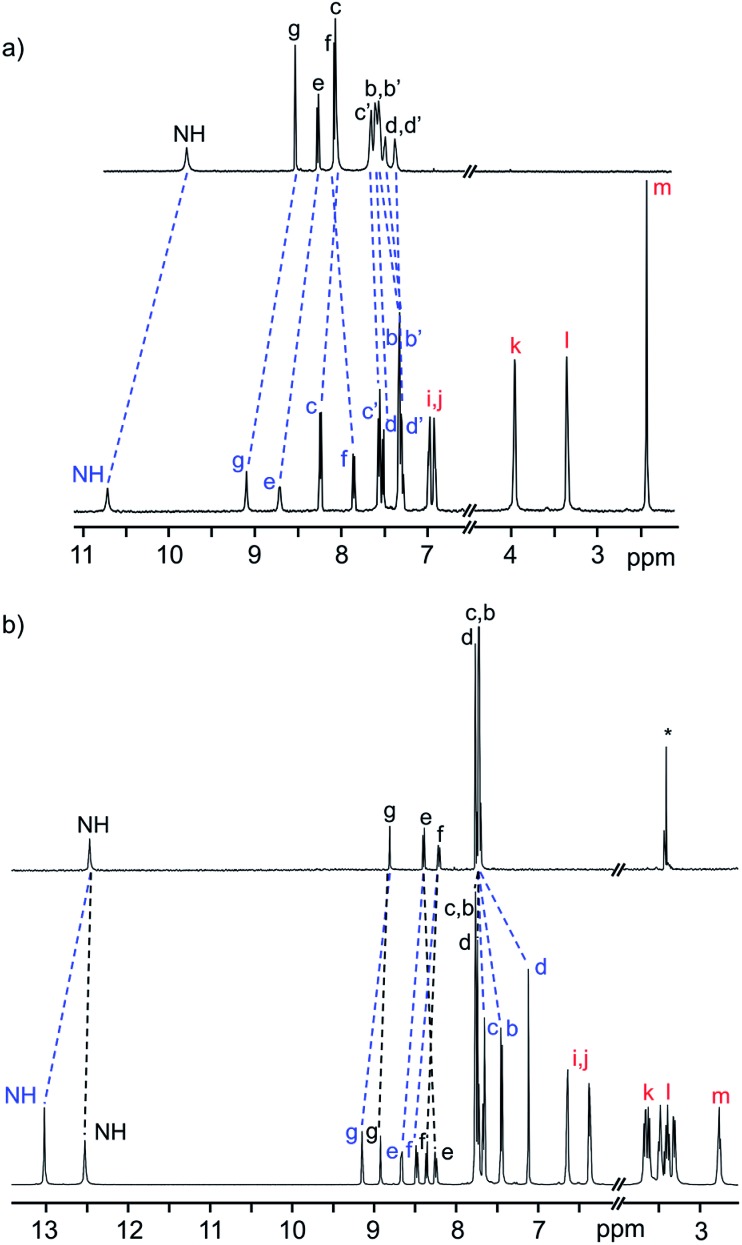
(a) ^1^H NMR spectra (500 MHz, CD_2_Cl_2_, 298 K) of naked axle of **R_5_** (top) and neutral [2]rotaxane **R_5_** (bottom). (b) ^1^H NMR spectra (500 MHz, CD_3_CN, 298 K) of naked axle of [**R_5_**-H_2_]^2+^ (top) and dicationic [2]rotaxane [**R_5_**-H_2_]^2+^ (bottom). The change in chemical shifts due to threading of the **DB24C8** wheel on the axle are highlighted. Colour key: blue = [2]rotaxane axle, red = crown ether wheel, black = naked axle. H-atom labels are as shown in Scheme 1.

The ^1^H NMR spectra of the neutral [2]rotaxanes display resonances for both axle and wheel protons that are indicative of molecular shuttling at a rate that is fast compared to the NMR timescale. As an example, Fig. 2a shows that for **R_5_** there are significant interactions resulting from hydrogen bonding between the axle and wheel – for example a down field shift of the NH peak from 9.77 to 10.69 ppm – but only averaged signals are observed.

For the dicationic version [**R_5_**-H_2_]^2+^, the interactions between axle and wheel are much stronger due to additional ion-dipole and π-stacking interactions and the ^1^H NMR spectra show resonances for all axle protons as the rate of shuttling is now slow on the NMR timescale; see Fig. 2b.

**Fig. 3:**
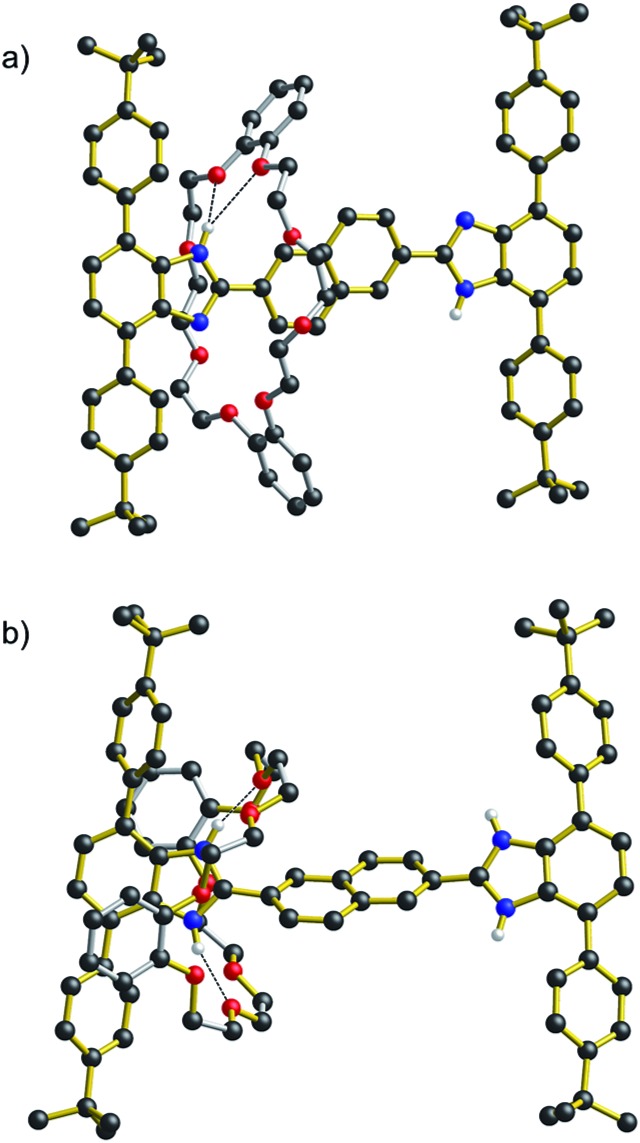
Ball-and-stick representations of the X-ray structures of [2]rotaxanes: (a) **R_5_** (*n* = Np) and (b) [**R_5_**-H_2_][BF_4_]_2_ (*n* = Np). Only H-atoms involved in hydrogen bonds are shown for clarity. Color key: red = O; blue = N, black = C; white = H; gold bonds = axle; silver bonds = wheel.

An analysis of the non-covalent interactions between axle and wheel can be obtained from the solid-state structure.‡
‡Complete details of the four X-ray structures can be obtained from the Cambridge Crystallographic Data Centre at http://www.ccdc.cam.ac.uk for CCDC accession numbers 1563641–1563644.
Fig. 3 shows the results of X-ray diffraction experiments for both the neutral and dicationic pair **R_5_** and [**R_5_**-H_2_][BF_4_]_2_. Most importantly, the structures verify the array of non-covalent interactions observed from the solution NMR studies. In the neutral species **R_5_**, the only significant interaction is a bifurcated hydrogen bond between the benzimidazole NH and crown ether O-atoms, while for the dication, [**R_5_**-H_2_]^2+^, the **DB24C8** wheel is clamped around the benzimidazolium core and involved in extensive charge-assisted hydrogen bonding accompanied by π-stacking interactions.^12^

### Measurement of shuttling rates

Shuttling rates for neutral and dicationic [2]rotaxanes were measured using ^1^H NMR spectroscopy. Since for the neutral series, **R_1_–R_5_**, the rate of shuttling was fast on the NMR timescale, variable temperature studies were used to obtain rates at different temperatures and these fit to the Eyring equation (see ESI†). Since both signals from complexed and uncomplexed species were observed in the ^1^H NMR spectra for the dicationic species [**R_2_**-H_2_]^2+^ – [**R_5_**-H_2_]^2+^, EXSY experiments were used to determine the rates of shuttling at room temperature. Table 1 summarizes the shuttling rates and energy barriers for both the neutral and dicationic series.

**Table 1:** Shuttling data from ^1^H NMR experiments for (**R_1_–R_5_**)^*a*^ in CD_2_Cl_2_ and ([**R_1_**-H_2_]^2+^ – [**R_5_**-H_2_]^2+^)^*b*^ in CD_3_CN

[2]Rotaxane	*k* (s^–1^)	Δ*G*^≠^ (kcal mol^–1^)
**R_1_**	1.0 × 10^7^	7.7^*c*^
**R_2_**	6.2 × 10^3^	9.0
**R_3_**	3.3 × 10^3^	9.6
**R_4_**	4.7 × 10^3^	9.2
**R_5_**	3.1 × 10^3^	9.8
[**R_1_**-H_2_]^2+^	17.0 × 10^–1^	17.3^*d*^
[**R_2_**-H_2_]^2+^	6.1 × 10^–3^	20.2^*e*^
[**R_3_**-H_2_]^2+^	7.8 × 10^–3^	20.1^*e*^
[**R_4_**-H_2_]^2+^	9.0 × 10^–3^	19.8^*f*^
[**R_5_**-H_2_]^2+^	12.4 × 10^–3^	20.0^*d*^

^*a*^Obtained from fit to VT data; reported at coalescence temperature, see ESI.

^*b*^Obtained from 2D EXSY data; reported at RT.

^*c*^Estimated from coalescence temperature see ESI.

^*d*^298 K.

^*e*^295 K.

^*f*^293 K.

**Fig. 4:**
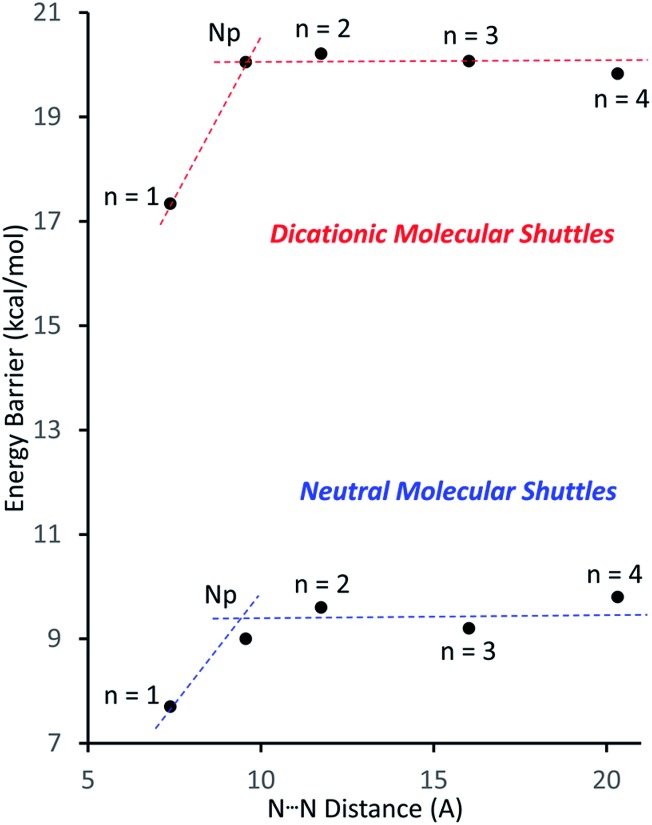
Plots of the energy barrier to translational motion in the molecular shuttles *versus* the distance between N-atoms on opposite ends of the track. Dotted lines are for visualisation purposes only (*n* = number of phenyl rings, Np = naphthyl).

Fig. 4 shows a plot of the energy barriers to shuttling, for [2]rotaxane molecular shuttles (neutral and charged) as a function of the distance between the benzimidazole N-atoms on the axle. For the most part, the data corroborates ‘Hirose and co-workers’ conclusion that axle length does not affect the shuttling rate. The neutral species **R_2_–R_5_** have a fairly low barrier to shuttling of approximately 9.7 kcal mol^–1^ (blue horizontal line), while the dicationic compounds [**R_2_**-H_2_]^2+^ – [**R_5_**-H_2_]^2+^ have a substantially larger energy barrier of approximately 20.1 kcal mol^–1^ (red horizontal line). Surprisingly, for both the neutral and dicationic species, it appears that the energy barriers for the shortest (*n* = 1) compounds are outliers. They appear to have significantly lower energy barriers. In order to try and understand these observations, we turned to DFT calculations.

## DFT calculations

Energy barriers to molecular shuttling and transition state structures were determined for two sets of molecular shuttles, **R_1_–R_4_** and [**R_1_**-H_2_]^2+^ – [**R_4_**-H_2_]^2+^, with varying track lengths defined by *n* = 1–4 phenyl rings using DFT calculations (B3LYP/6-31G**, PCM *ε* = 8.93). The calculated energy barrier for both (neutral and charged) series showed the same trends as the experimentally obtained data, where for *n* = 2–4 the shuttling energies are similar and fairly insensitive to the distance between the recognition sites (see ESI†). However, as was observed for the experimental measurements, the calculated energy barriers for the molecular shuttling were significantly lower for the shortest *n* = 1 systems (neutral and charged).

**Fig. 5:**
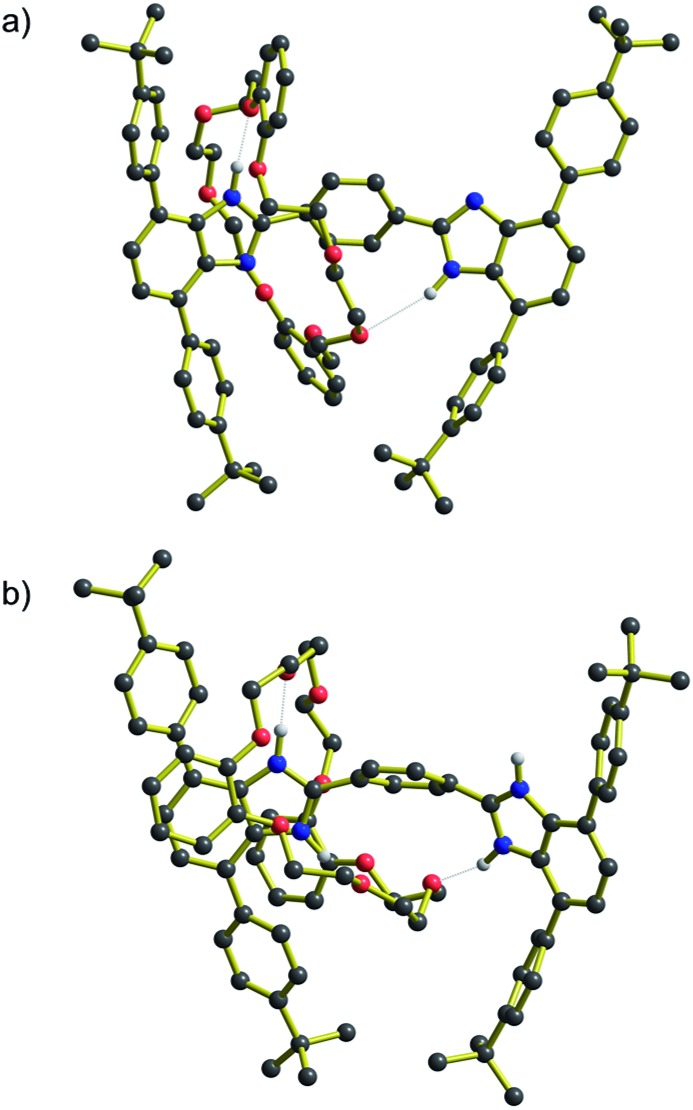
Structural representations of the transition state (TS) for molecular shuttling as determined by DFT calculations. (a) **R_1_** and (b) [**R_1_**-H_2_]^2+^. NH···O interactions are shown as dotted lines.

Moreover, DFT geometry optimizations of the transition states (TS) for these molecules provided a rational explanation for the observed differences between the energy barriers. For both the **R_1_** and [**R_1_**-H_2_]^2+^, the TS structures showed that the short length of the axles allows interaction of the crown ether wheel with both recognition sites at the same time. These interactions are stabilized by hydrogen bonds between the H-shaped axle and the **DB24C8** macrocycle with NH···O values between 1.9 and 2.7 Å. The predicted TS structures for **R_1_** and [**R_1_**-H_2_]^2+^ are shown in Fig. 5. These TS structures provide a shortcut in terms of the energy barrier to shuttling as interactions holding the wheel unit in place are never completely severed. On the other hand, for *n* = 2–4 this type of geometrical structure for the TS is not possible, see ESI.† Furthermore, it is interesting that the structures at the TS position show that for *n* = 1 (neutral and charged) the H-shaped axle bends, which allows both recognitions sites to be closer to the **DB24C8** macrocycle (see Fig. 5), but for *n* = 2–4 (neutral and charged) the H-shaped axle remains straight, the recognition sites are further apart and the wheel interacts only with the central aromatic rings in the TS geometry (see ESI†).

### Further experimental evidence: transition state “snap-shots”

For all of the neutral molecular shuttles, the energy barriers to translational motion (measured in CD_2_Cl_2_) are quite low and this is undoubtedly due to the minimal interactions that bind the wheel to the recognition site – primarily a single NH···O hydrogen bond and some weak CH···O interactions. In this respect it should be noted that previous attempts to measure association constants for [2]pseudorotaxanes formation between neutral T-shaped benzimidazole axles and 24-membered crown ether wheels were unsuccessful, even in completely non-competitive solvents such as toluene.^13^ Only charged benzimidazolium axles have been successfully used for the template preparation of H-shaped MIMs.

**Fig. 6:**
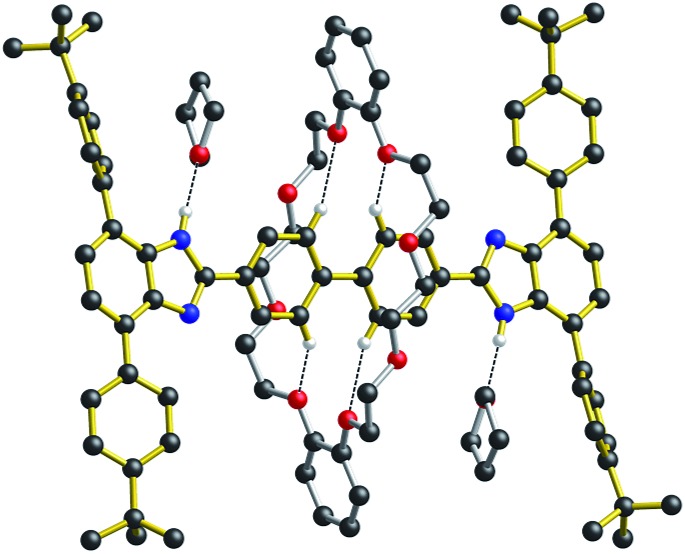
Ball-and-stick representation of the single-crystal X-ray structure of the [2]rotaxane molecular shuttle **R_2_** (*n* = 2) crystallized from THF (crystal formula **R_2_** (THF)_4_). Only H-atoms involved in hydrogen bonds are shown for clarity. Color key: red = O; blue = N, black = C; white = H; gold bonds = axle; silver bonds = wheel.

It was therefore interesting to note that when the neutral molecular shuttle **R_2_** was crystallized from the hydrogen-bond accepting solvent THF, the solvent interacts with the benzimidazole NH groups rather than the **DB24C8** macrocycle.^14^Fig. 6 shows the X-ray crystal structure of **R_2_**·(THF)_4_ in which a molecule of THF hydrogen bonds to each of the benzimidazole NH groups and the **DB24C8** macrocycle is displaced to a position between the two axle phenyl groups. This is presumably a position where only very weak interactions are possible between phenyl H-atoms and crown ether O-atoms. This can therefore be thought of as “snap-shot” of the crown ether position during a cycle of molecular shuttling between the two recognition sites along a rigid biphenyl axle. Indeed, this positioning of the crown ether on the axle is very similar to that calculated for the transition state structure of this shuttling process using DFT (see ESI†).

The identification by DFT and inference from experiment that for the shorter (*n* = 1) molecular shuttles the macrocyclic ring can adopt a conformation that allows interaction with both recognition states simultaneously is an important aspect of the shuttling mechanism for these rigid molecular systems. In an effort to induce this type of ambivalent structural conformation between axle and wheel, a model [2]rotaxane **R_1_**-Br with one phenyl ring as the spacer was prepared. This compound lacks: (1) the aromatic groups at the 4- and 7-positions of the benzimidazole units of the axle as they are replaced by Br-atoms and (2) the aromatic groups of the crown ether since the wheel is simply **24C8**.^13^

**Fig. 7:**
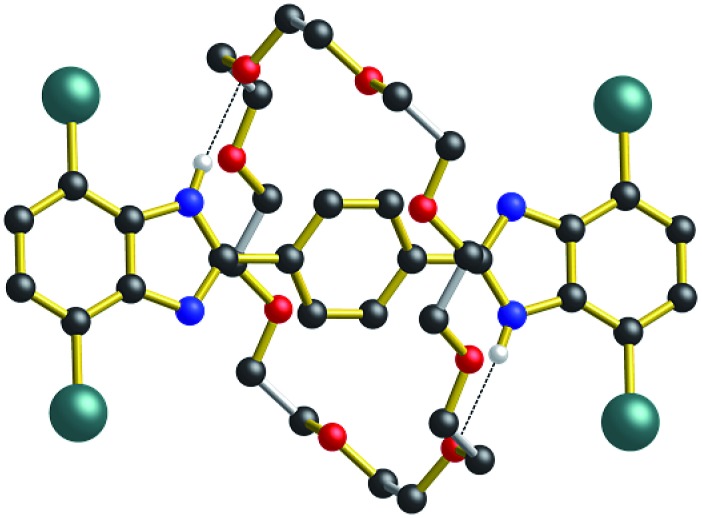
Ball-and-stick representation of the single-crystal X-ray structure of the [2]rotaxane molecular shuttle **R_1_**-Br (*n* = 1). Only H-atoms involved in hydrogen bonds are shown for clarity. Color key: teal = bromine, red = O; blue = N, black = C; white = H; gold bonds = axle; silver bonds = wheel.

As shown for the X-ray structure of **R_1_**-Br in Fig. 7, the absence of secondary CH···O stabilizing interactions between axle and wheel – only the primary NH···O hydrogen bonds remain – the flexible **24C8** macrocyclic can indeed span the two recognition sites and is involved in a centrosymmetric conformation utilizing two NH···O hydrogen bonds to simultaneously interact with both benzimidazole sites; conceptually mimicking the transition state calculated for **R_1_** using DFT.^14^

## Conclusions

This study not only corroborates ‘Hirose and co-workers’ conclusions about the translational motion in molecular shuttles with a rigid axle – that the length of the axle does not affect the shuttling rate^8^ – it expands upon that single system demonstrating that, with one significant exception, both the neutral and dicationic forms of a class of rigid H-shaped [2]rotaxanes also show length-independent shuttling rates. Most importantly, we were able to identify a mechanism for shuttling that can act to lower the usual energy barrier if the axle length is short enough, even in a very rigid system. Finally, a pair of single-crystal X-ray structures provide interesting mechanistic insight into: (1) what the transition state structure for the passage of a **DB24C8** macrocycle between two well separated neutral recognition sites (two phenyl rings) during a shuttling event might look like and (2) what the structure of a “short-cut” transition state for a neutral shuttle with a short and rigid axle (one phenyl ring) could resemble.

## Conflicts of interest

There are no conflicts to declare.

## Supplementary Material

Supplementary information

Crystal structure data
