# Supplementary material for: Influence of axle length on the rate and mechanism of shuttling in rigid H-shaped [2]rotaxanes
Source: Chem Sci. 2017 Sep 25;8(11):7718–23. doi: 10.1039/c7sc03736h (PMC5851341; doi:10.1039/c7sc03736h)
Supplement: Supplementary file 1 [file SC-008-C7SC03736H-s001.pdf]

Electronic Supplementary Material (ESI) for Chemical Science.
This journal is © The Royal Society of Chemistry 2017

Electronic Supplementary Information for:

Influence of axle length on the rate and mechanism of shuttling in rigid H-shaped [2]rotaxanes

Ghazale Gholami,[a] Kelong Zhu,*[b] Giorgio Baggi[a], Eduardo Schott*[c], Ximena Zarate*[d], Stephen J. Loeb*[a]

[a]*Department of Chemistry & Biochemistry, University of Windsor, Windsor, Ontario, N9B 3P4, Canada*
[b]*School of Chemistry, Sun Yat-Sen University, Guangzhou, 510275, P. R. China*

[c]*Departamento de Química Inorgánica, Facultad de Química, Pontificia Universidad Católica de Chile, Avenida Vicuña Mackenna, 4860, Santiago, Chile.*

[d]*Instituto de Ciencias Químicas Aplicadas, Facultad de Ingeniería, Universidad Autónoma de Chile. Avenida Pedro de Valdivia 641, Santiago, Chile*

**Table of Contents**	Page
General Comments	S2
Synthetic Steps	S3
Synthesis, ^{1}H and ^{13}C NMR spectra of **2**	S5
Synthesis, ^{1}H and ^{13}C NMR spectra of **3**	S7
Synthesis, ^{1}H and ^{13}C NMR spectra of [**3**-H][BF_4]	S9
Synthesis, ^{1}H and ^{13}C NMR spectra of $\mathbf{R_2}$	S11
Synthesis, ^{1}H and ^{13}C NMR spectra of [$\mathbf{R_2}$-H_2][BF_4]$_2$	S13
Synthesis, ^{1}H and ^{13}C NMR spectra of **4**	S15
Synthesis, ^{1}H and ^{13}C NMR spectra of [**4**-H][BF_4]	S17
Synthesis, ^{1}H and ^{13}C NMR spectra of $\mathbf{R_3}$	S19
Synthesis, ^{1}H and ^{13}C NMR spectra of [$\mathbf{R_3}$-H_2][BF_4]$_2$	S21
Synthesis, ^{1}H and ^{13}C NMR spectra of **5**	S23
Synthesis, ^{1}H and ^{13}C NMR spectra of **5b**	S25
Synthesis, ^{1}H and ^{13}C NMR spectra of [**5b**-H][BF_4]	S27
Synthesis, ^{1}H and ^{13}C NMR spectra of $\mathbf{R_4}$	S29
Synthesis, ^{1}H and ^{13}C NMR spectra of [$\mathbf{R_4}$-H_2][BF_4]$_2$	S31
Synthesis, ^{1}H and ^{13}C NMR spectra of **6**	S33
Synthesis, ^{1}H and ^{13}C NMR spectra of [**6**-H][BF_4]	S35
Synthesis, ^{1}H and ^{13}C NMR spectra of $\mathbf{R_5}$	S37
Synthesis, ^{1}H and ^{13}C NMR spectra of [$\mathbf{R_5}$-H_2][BF_4]$_2$	S40
Determination of Shuttling Rates for Neutral Molecular Shuttles	S43
Determination of Shuttling Rates for Dicationic Molecular Shuttles	S52
Details of Single-Crystal X-ray Structure Determinations	S56
Methods and Computational Details	S60
References	S62

General Comments

4,7-Di(4′-t-butylphenyl)-benzothiodiazole[S1], 1,2-diamino-3,6-di(4′-t-butylphenyl)-benzene (**1**)[S2], terphenyl-4,4′-dicarbaldehyde[S3], 4′-bromo-4-triphenylbenzaldehyde[S4] and naphthalene-2,6-dicarbaldehyde[S5] were synthesized according to literature methods. Dibenzo[24]crown-8 ether **DB24C8** was purchased from TCI Chemical. 4,4′-Dibromobiphenyl, 1,4-dibromobenzene and 4-t-butylphenylboronic acid were purchased from Sigma-Aldrich. The solvents were dried and distilled prior to use. Deuterated solvents were obtained from Cambridge Isotope Laboratories and used as received. ^{1}H, ^{13}C, and 2D NMR spectra were recorded on Brüker Avance 500 or 300 instruments. All peak positions are listed in ppm relative to TMS. Melting point measurements were performed on MPA100 melting point apparatus. Column chromatography was performed using Silicycle Ultra-Pure Silica Gel (230−400 mesh).

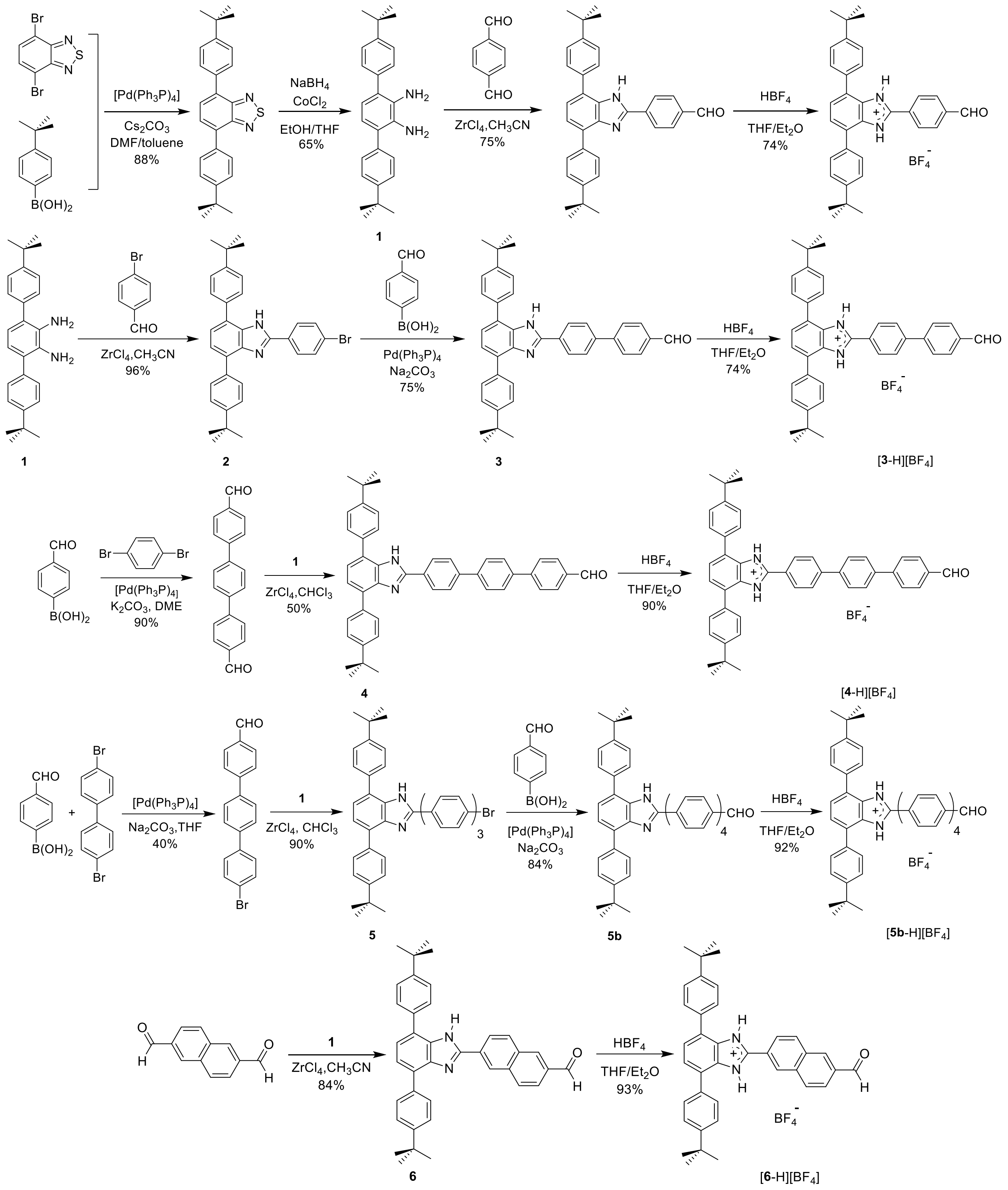

Scheme S1. Synthetic steps involved in the preparation of precursor aldehydes (n = 1-4, Np).

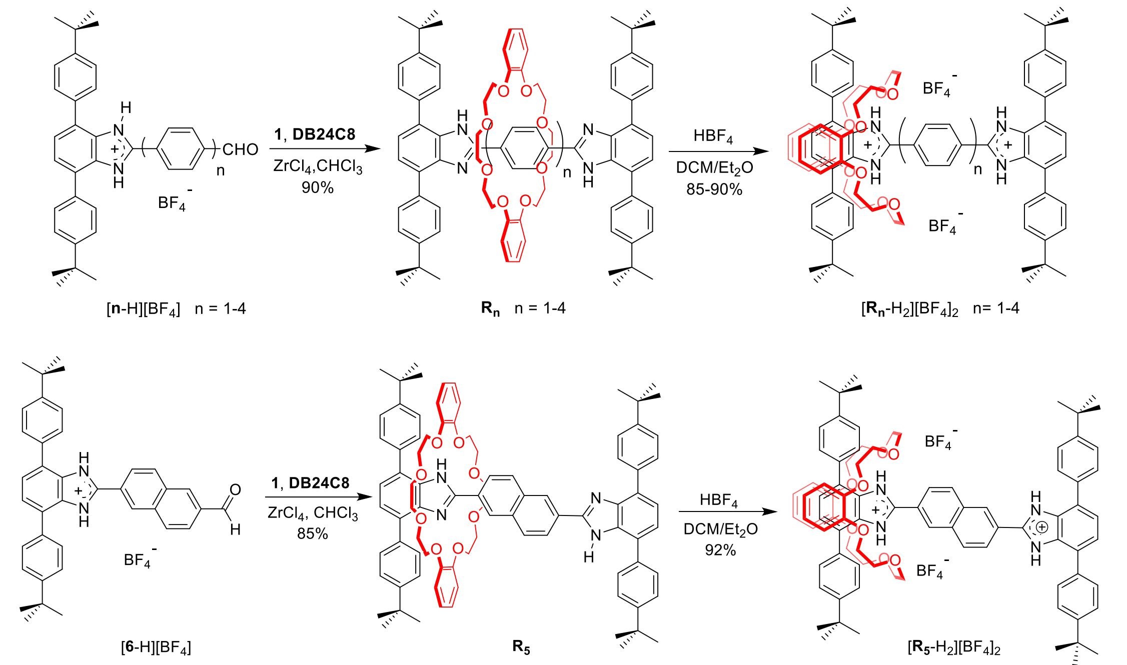

Scheme S2. Synthesis of neutral [2]rotaxanes and dicationic [2]rotaxanes (n = 1-4, Np)

Synthesis of 2

$ZrCl_4$ (3.20 mg, 0.0136 mmol) was added to a round bottom flask containing **1** (505 mg, 0.136 mmol). Bromobenzaldehyde (25.3 mg. 0.136 mmol) was diluted with $CHCl_3$ in a small vial and then transferred to the round bottom flask containing **1**. The orange solution was stirred for 24 h at room temperature. Solvents were removed and a white solid residue was obtained. Yield 96% (70.0 mg). Mp: 257-259 °C. ^{1}H NMR (300 MHz, $CDCl_3$, 298 K) δ = 1.45 (s, 18H), 7.50 (s, 2H), 7.63, (m, 8H, J = 7.6 Hz), 7.97, (d, 4H, J = 7.9 Hz), 9.52 (br s, 1H).^{13}CNMR (300 MHz, $CDCl_3$ 298K) δ = 31.51, 34.79, 123.01, 124.57, 128.29, 128.52, 128.72, 132.23, 135.52, 150.37, 150.74. IR (neat): 3358, 3031, 2691, 2902, 2866, 1602, 1493, 1474, 1461, 1425, 1260 cm^{-1}. HR-MS (ESI): calcd for $[M+H]^+$, $[C_{33}H_{33}BrN_2]^+$, m/z = 537.1906, found m/z = 537.1905.

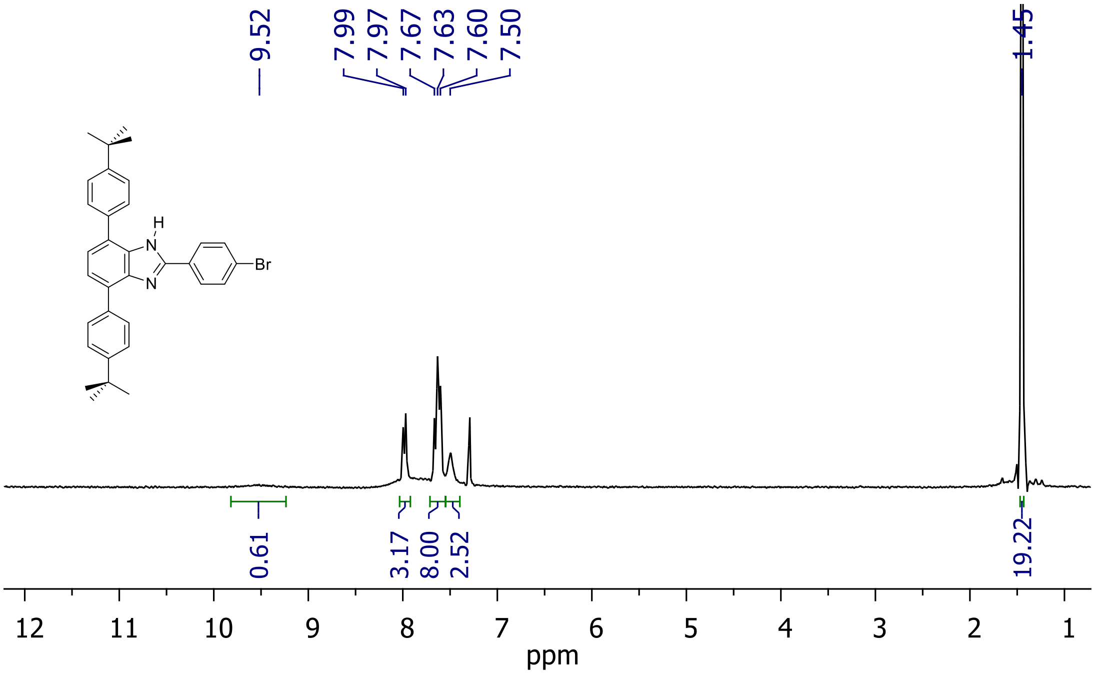

Figure S1. ^{1}H NMR spectrum of **2** (300 MHz, $CDCl_3$, 298 K).

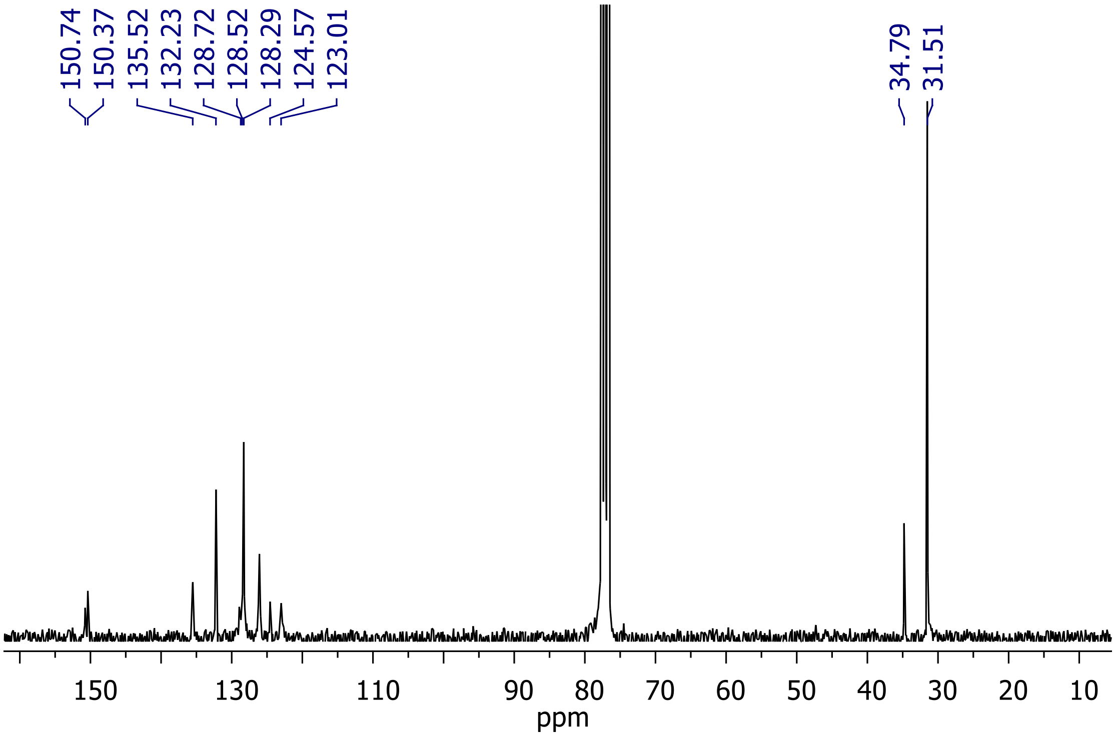

Figure S2. ^{13}C NMR spectrum of **2** (125 MHz, $CDCl_3$, 298 K).

Synthesis of 3

2 (100 mg, 0.178 mmol), 4-formylphenylboronic acid (69.46 mg, 0.3909 mmol) and [Pd(PPh_3)$_4$] (20.5 mg, 0.0178 mmol) were added to a 100 mL Schlenk flask, degassed and backfilled with N_2. A solution of 2M Na_2CO_3 (11 mL, 120 eq.) and THF (11 mL, 120 eq.) were added and the yellow solution was refluxed (80 °C) for 24 h. The solution was cooled to room temperature, the organic layer was extracted with ethyl acetate (2 x 50 mL) and then dried over anhydrous $MgSO_4$. CH_3CN (20 mL) was added, the solution heated to reflux and then cooled to room temperature. The resulting solids were filtered and air dried producing a yellow solid. Yield 75% (91 mg). Mp: 191-194°C. ^{1}H NMR (500 MHz, $CDCl_3$, 298 K) δ = 1.41 (s, 18H), 7.38 (d, 1H, J = 7.7 Hz), 7.55 (d, 1H, J = 7.7 Hz), 7.58 (d, 1H, J = 7.5), 7.56 (d, 2H, J = 7.5), 7.60 (d, 2H, J = 8.5 Hz), 7.64 (d, 2H, J = 8.5 Hz), 7.75 (d, 2H, J = 8.6) 7.80(d, 2H, J = 8.2 Hz), 7.97 (d, 2H, J = 8.45), 7.12(d, 2H, J = 8.5), 8.18 (d, 2H, J = 8.5), 9.63(s, 1H) 10.08(s, 1H). ^{13}C NMR (126 MHz, $CDCl_3$) δ = 191.94, 150.91, 150.29, 146.10, 142.30, 140.96, 135.67, 135.46, 133.26, 131.43, 130.37, 129.97, 128.98, 127.72, 127.63, 127.41, 126.32, 125.47, 124.44, 123.39, 122.28, 34.64, 31.41. HR-MS (ESI): calcd for [M+H]$^+$, [$C_{40}H_{38}N_2O$]$^+$, m/z = 563.3018, found m/z = 563.3032.

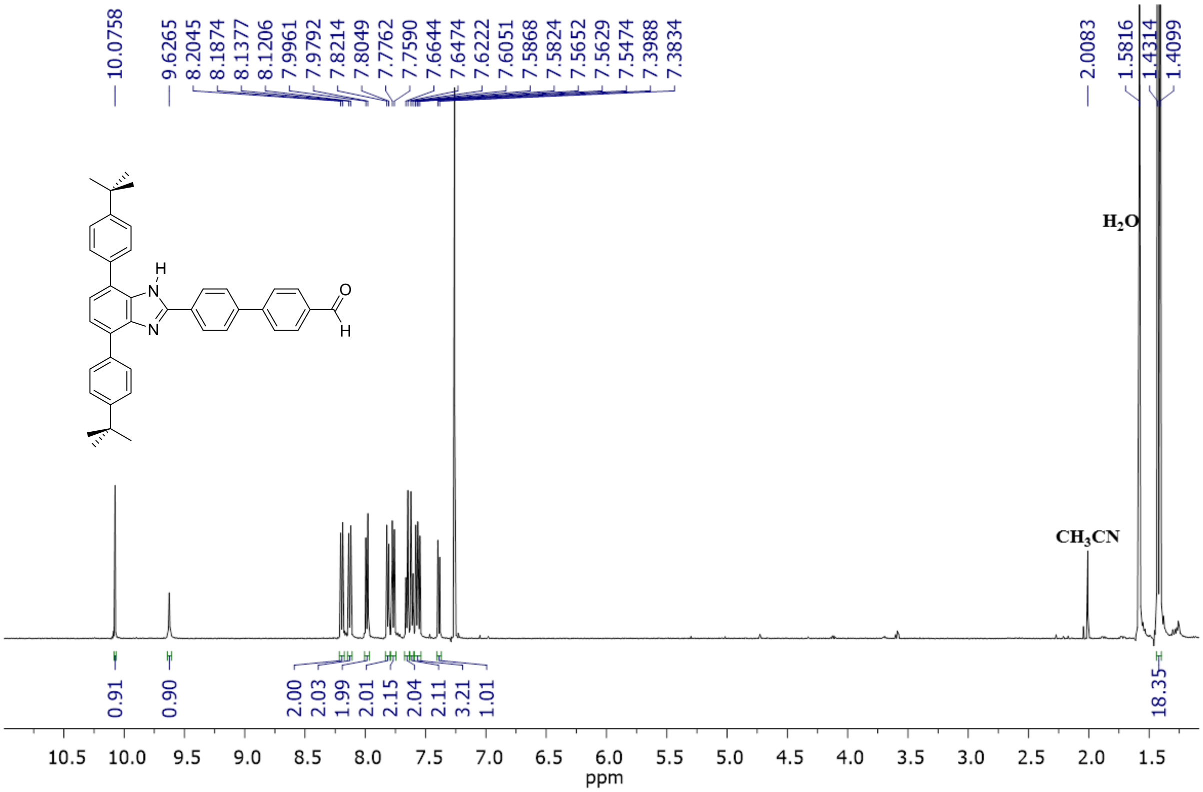

Figure S3. ^{1}H NMR spectrum of **3** (500 MHz, $CDCl_3$, 298 K).

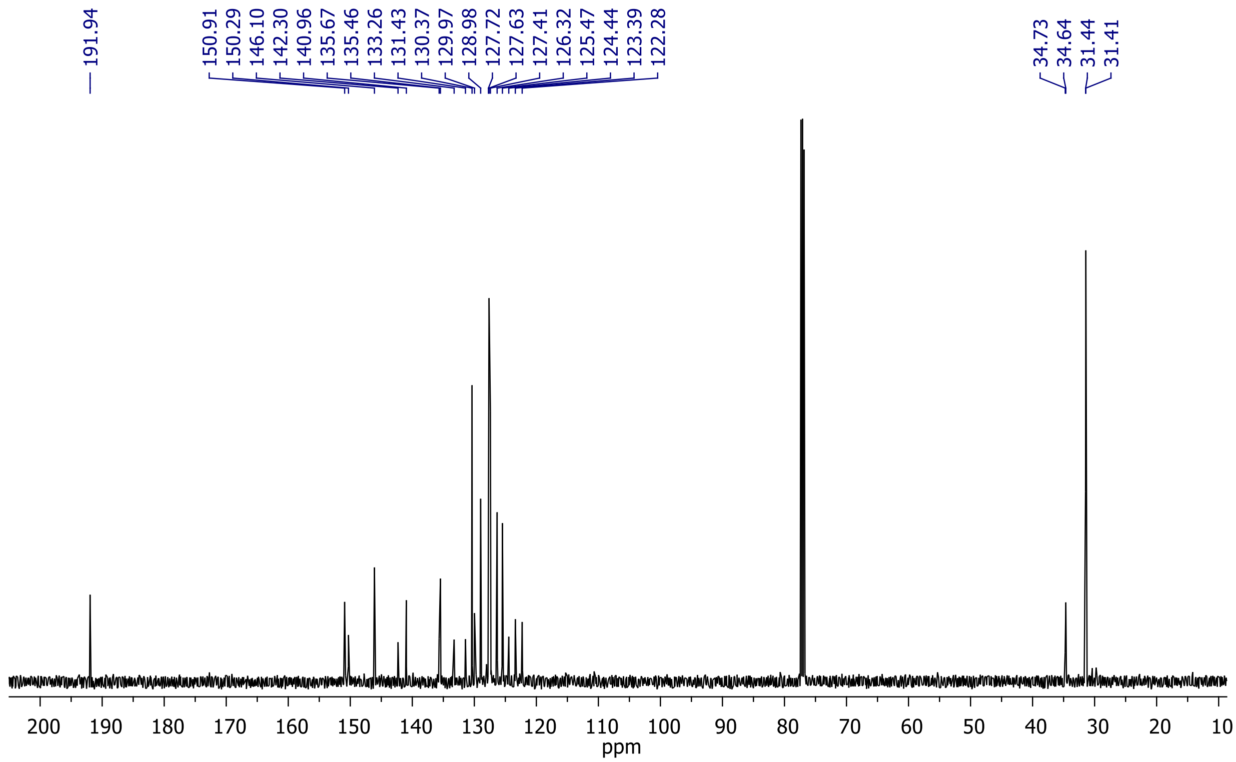

Figure S4. ^{13}C NMR spectrum of **3** (125 MHz, $CDCl_3$, 298 K).

Synthesis of [3-H][BF$_4$]

3 (118 mg, 0.220 mmol) was dissolved in diethyl ether (100 mL). Tetrafluoroboric acid diethyl ether complex (22 µL, 0.24 mmol) was added in a dropwise fashion. The resulting yellow solid was vacuum filtered and washed with ether. Yield 74% (87 mg). Mp: 214-217°C. ^{1}H NMR (500 MHz, CD_3CN, 298 K) δ = 1.42 (s, 18H), 7.69 (s, 10H), 7.95 (d, 2H, J = 8.3 Hz), 8.01 (dd, 4H, J = 8.03Hz), 8.15 (d, 2H, J = 8.6Hz), 10.10(s, 1H), 12.60 (br s, 2H). ^{13}C NMR (125 MHz, CD_3CN) δ = 192.32, 152.26, 151.06, 144.45, 132.50, 132.50, 130.41, 130.20, 129.94, 128.53, 128.21, 128.06, 127.59, 127.14, 126.43, 121.66, 121.66, 34.48, 30.60. HR-MS (ESI): calculated for [M+H]$^+$, [$C_{40}H_{38}N_2O$]$^+$, m/z = 563.3062, found m/z = 563.3063.

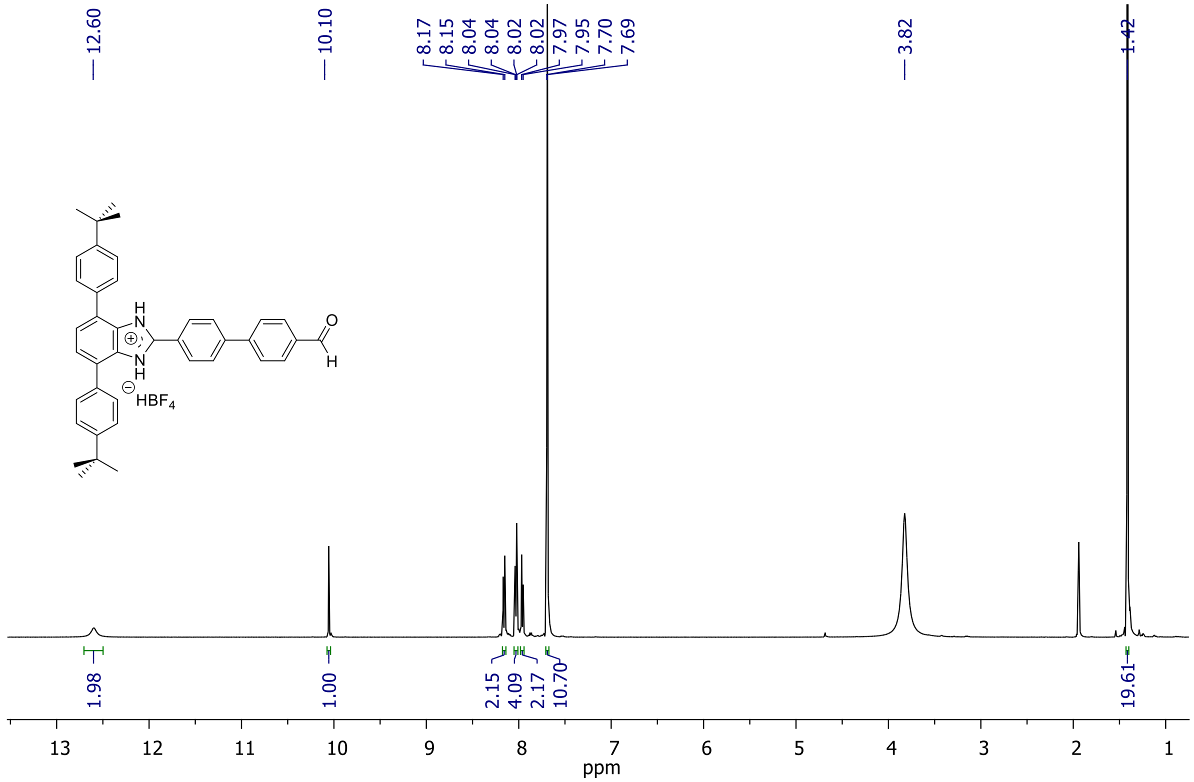

Figure S5. ^{1}H NMR spectrum of [**3**-H][BF_4] (500 MHz, CD_3CN, 298 K).

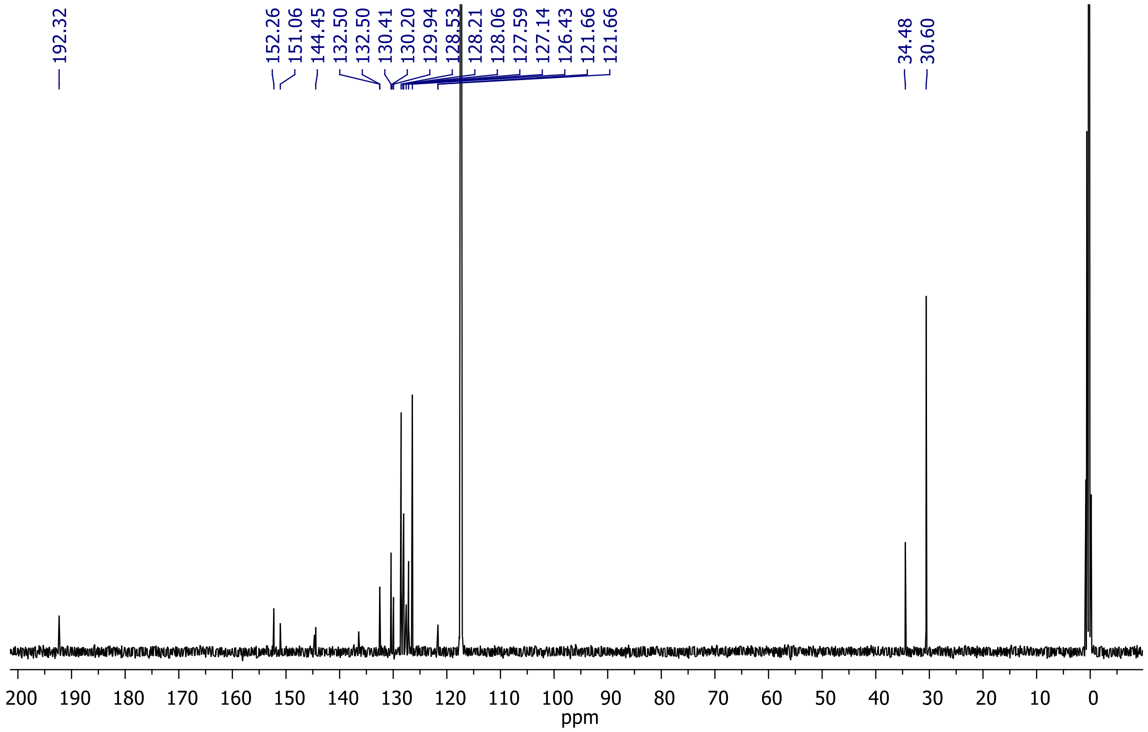

Figure S6. ^{13}C NMR spectrum of [**3**-H][BF_4] (125 MHz, CD_3CN).

Synthesis of R_2

DB24C8 (179 mg, 0.399 mmol) was added to [**3**-H][BF_4] (65 mg, 0.0997 mmol) in a round bottom flask. $CHCl_3$ (30 mL) was added and stirred at room temperature until a clear solution formed. 1,2-Diamino-3,6-di(4-t-butylphenyl)benzene **1** (41 mg, 0.1097 mmol) was added, followed by $ZrCl_4$ (26 mg, 0.1097 mmol) and the yellow solution was stirred at room temperature for 24 h. The solution was filtered, solvents were evaporated and CH_3CN (30 mL) was added to dissolve the solids. Et_3N (0.2 mL) was slowly added and the formation of crystals was observed. The solution was filtered and air dried producing a white solid. Yield 45% (65 mg). Mp >300°C. ^{1}H NMR (500 MHz, CD_2Cl_2, 298 K) δ = 1.29 (d,36H), 2.62 (s,8H), 3.45 (s, 8H), 4.01 (s, 8H), 6.91 (m, 4H), 6.98 (m,4H), 7.30 (d, 2H, J= 5.7 Hz), 7.32 (d, 4H, J= 8.4 Hz), 7.37 (d, 4H, J= 8.4 Hz), 7.55 (d, 2H, J= 7.3 Hz), 7.57 (d, 4H, J= 8.6 Hz), 7.80 (d, 4H, J= 8.4 Hz), 8.23 (d, 4H, J= 8.2 Hz), 8.66 (d, 4H, J= 8.5 Hz), 10.7 (s, 2H). ^{13}C NMR (300 MHz, CDCl3, 298K) δ = NMR (125 MHz, CD_2Cl_2) δ 152.46, 150.51, 148.86, 142.83, 135.93, 130.89, 129.66, 129.34, 128.18, 126.81, 126.23, 125.54, 123.43, 121.55, 121.26, 112.12, 69.38, 68.91, 68.60, 34.81, 31.53, 31.43. HR-MS (ESI): calcd for $[M+H]^+$, $[C_{90}H_{98}N_4O_8]^+$, m/z = 1363.7463, found m/z = 1363.7483.

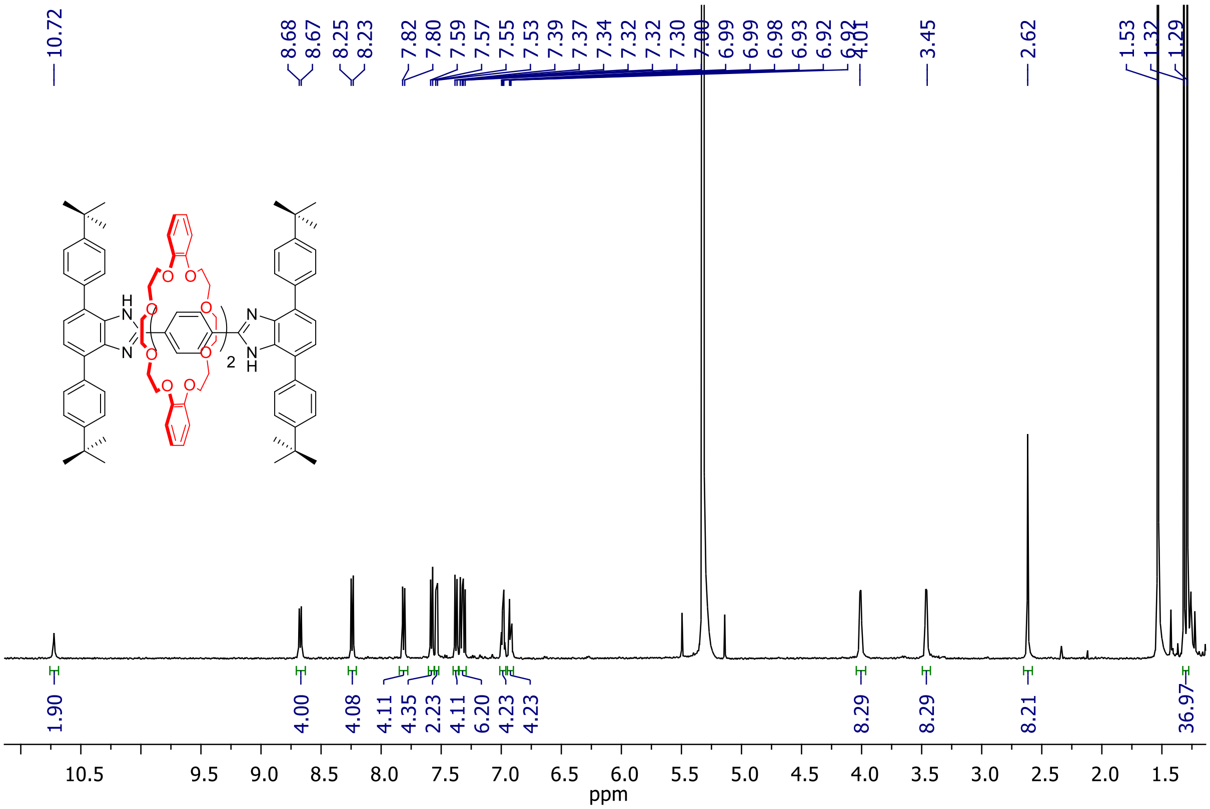

Figure S7. ^{1}H NMR spectrum of **R_2** (500 MHz, CD_2Cl_2, 298 K).

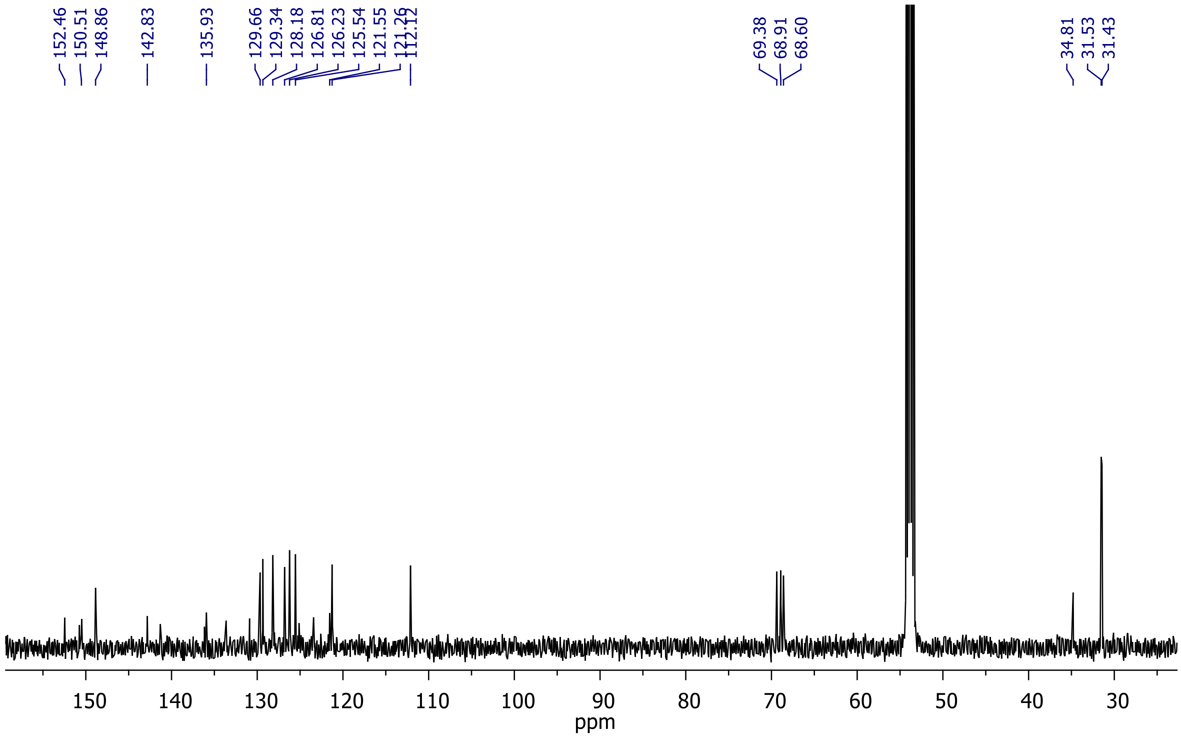

Figure S8. ^{13}C NMR spectrum of **R_2** (125 MHz, CD_2Cl_2, 298 K).

Synthesis of [R_2-H_2][BF_4]$_2$

Tetrafluoroboric acid (2.8 µL, 0.0322 mmol) was added dropwise to **R_2** which was dissolved in CH_3CN (1 mL). After 30 min, the CH_3CN was removed and diethyl ether (20 mL) was added. The solution was sonicated and vacuum filtered. Yield 66% (13.7 mg). Mp: 217-220°C. ^{1}H NMR (500 MHz, CD_3CN, 298 K) δ =1.44 (s,36H), 2.95 (m,4H), 3.36 (m, 8H), 3.52 (m, 4H), 3.65 (m, 8H), 6.63 (m, 4H), 6.62 (m,4H), 7.10 (s,1H),7.43 (d, 4H, J= 7.3 Hz), 7.62 (d, 4H, J= 7.3 Hz), 7.72 (d, 10H, J= 7.6 Hz), 8.12 (m, 4H, J= 8.1 Hz), 8.23 (d, 2H, J= 8.2 Hz), 8.54 (d, 2H, J= 8.5 Hz), 12.32 (s, 2H), 12.89 (s, 2H). ^{13}C NMR (75 MHz, CD_3CN, 298 K) δ = 152.36, 152.11, 151.13, 150.95, 147.34, 142.23, 132.61, 132.33, 130.60, 129.96, 129.28, 128.75, 128.64, 128.10, 127.31, 126.72, 126.54, 126.34, 125.90, 124.91, 120.84, 112.49, 69.98, 69.74, 67.86, 34.55, 30.68. HR-MS (ESI): calculated for $[M+H]^{2+}$, $[C_{90}H_{100}N_4O_8]^{2+}$, m/$z$ = 682.3765, found m/z = 682.3756.

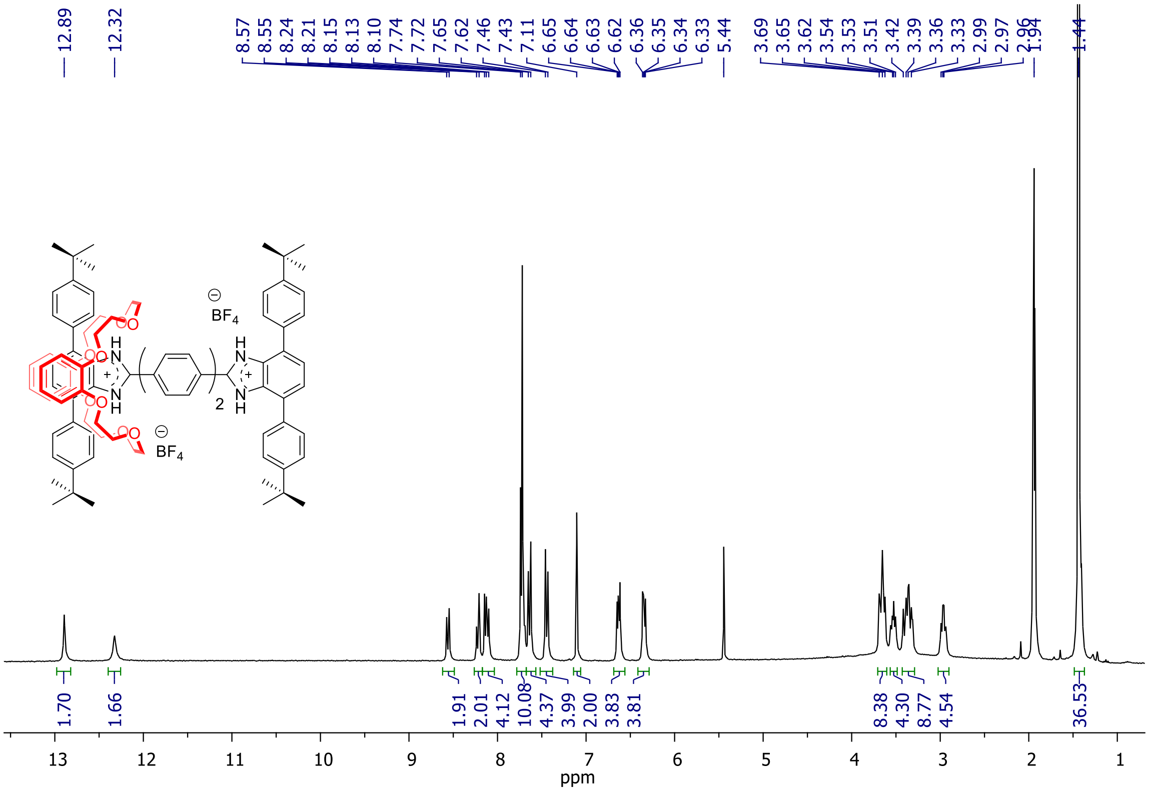

Figure S9. ^{1}H NMR spectrum of [**R₂**-H₂][BF₄]₂ (500 MHz, CD_3CN, 298 K).

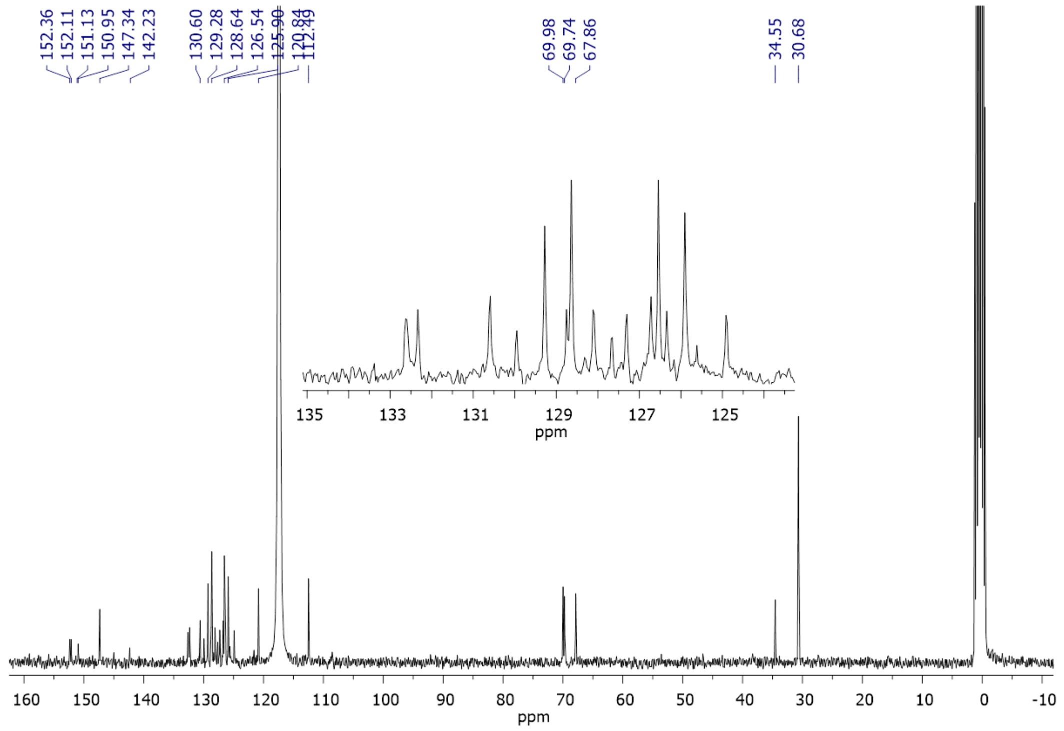

Figure S10. ^{13}C NMR spectrum of [**R₂**-H₂][BF₄]₂ (75 MHz, CD_3CN, 298 K).

Synthesis of 4

1 (65 mg, 0.174mmol) and terphenyl-4,4′-dicarbaldehyde (0.25 g, 0.873 mmol) were dissolved in $CHCl_3$. $ZrCl_4$ (4 mg, 0.0174mmol) was added. After 24 h stirring, the solution was filtered and solvent removed. The crude product was purified by column chromatography (SiO_2, dichloromethane). Yield: 100 mg, 89%. Mp: 185-190 °C. ^{1}H NMR (300 MHz, $CDCl_3$, 298 K) δ = 1.42 (s, 18H), 7.47 (s, 2H), 7.58 (d, 4H, *J*= 7.5 Hz), 7.76 (m, 8H), 7.97 (d, 2H *J*= 7.9 Hz), 8.1 (d, 2H *J*= 8 Hz), 10.07 (s, 1H). ^{13}C NMR (126 MHz, $CDCl_3$, 298 K) δ = 192.00, 151.12, 151.05, 146.58, 142.12, 141.89, 140.37, 139.69, 139.16, 135.63, 135.40, 130.46, 129.20, 127.98, 127.77, 127.66, 127.54, 127.33, 34.77, 31.50. H-MS (ESI): calcd for $[M+H]^+$, $[C_{46}H_{43}N_2O]^+$, *m/z* = 639.3370, found *m/z* = 639.3375.

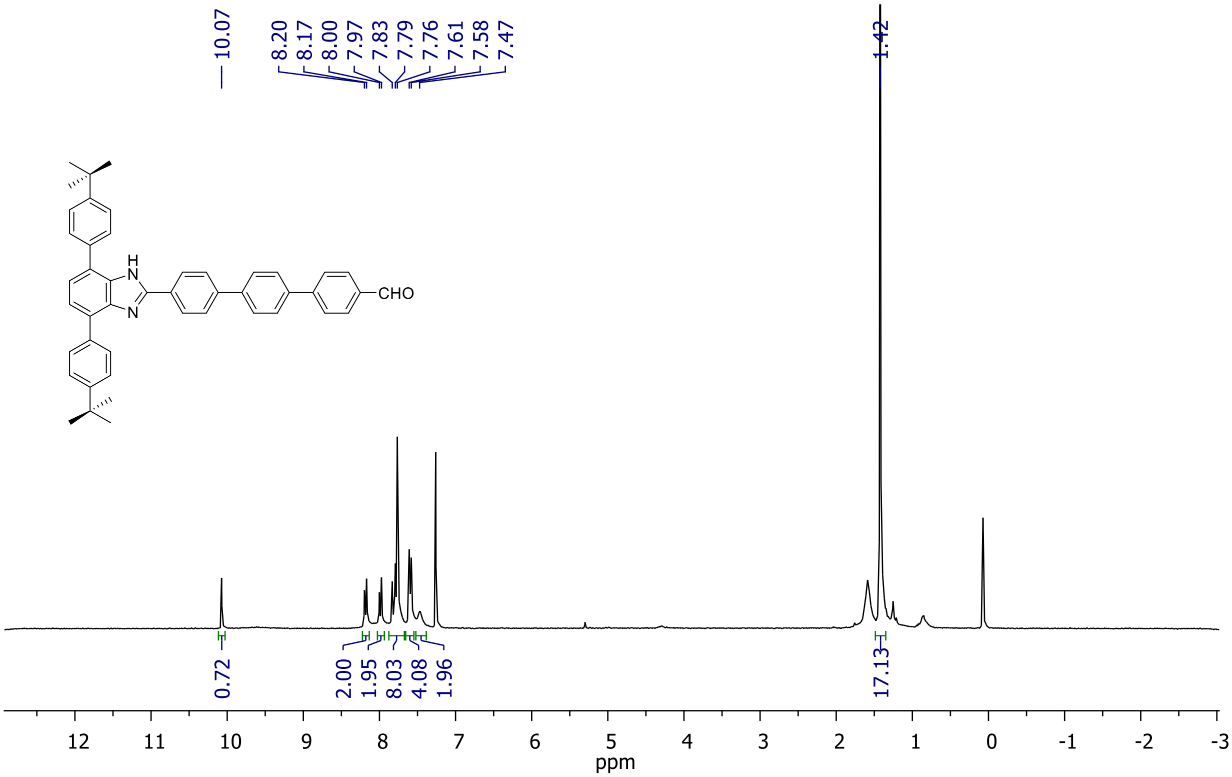

Figure S11. ^{1}H NMR spectrum of **4** (300 MHz, $CDCl_3$, 298 K).

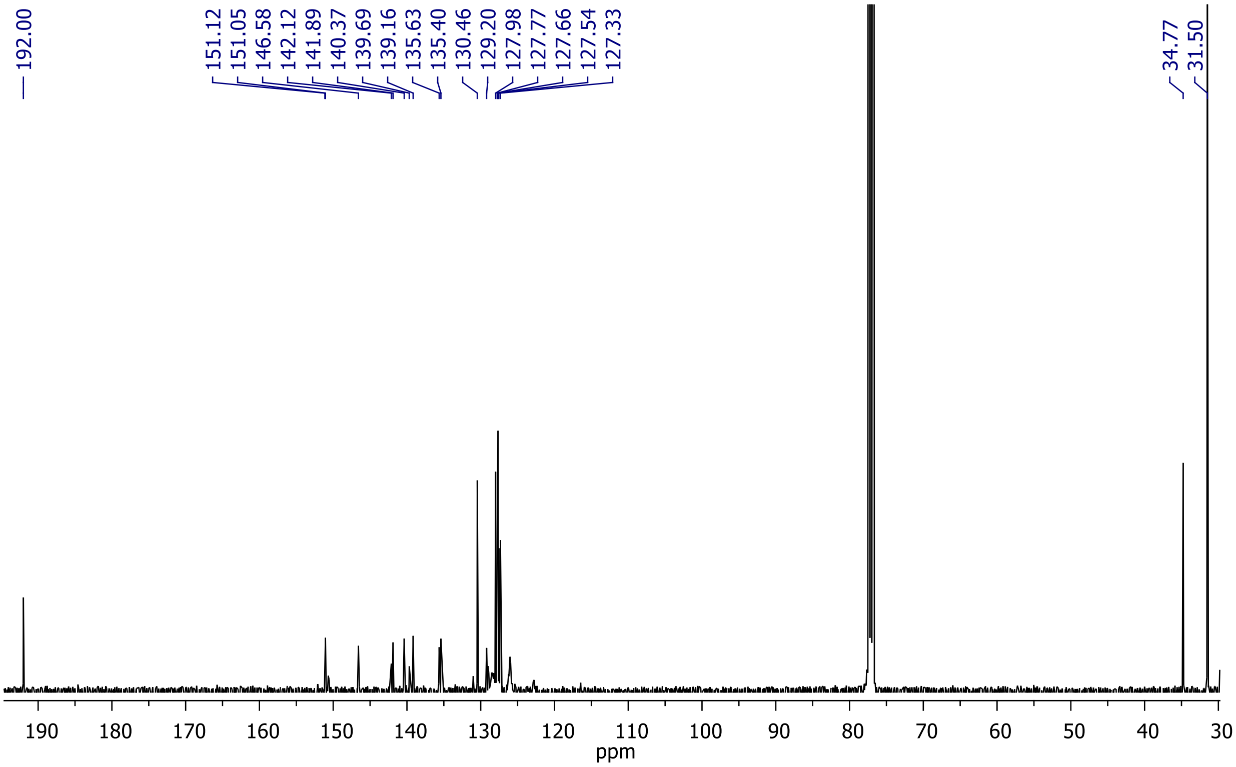

Figure S12. ^{13}C NMR spectrum of **4** (125 MHz, $CDCl_3$, 298 K).

Synthesis of [4-H][BF_4]

4 (100 mg, 0.16 mmol) was dissolved in THF (15 mL) and tetrafluoroboric acid diethyl ether complex (24 µL, 0.176 mmol) was added. The resulting yellow solid was vacuum filtered and washed with diethyl ether. Yield 83% (93 mg). Mp: >300°C, ^{1}H NMR (500 MHz, CD_3CN, 298 K) δ = 1.44 (s, 18H), 7.72 (m, 10H), 7.93 (m, 2H), 7.98 (s, 2H), 8.03 (d, 1H, J = 6.5 Hz), 8.07 (m, 2H, 10.07), 8.17 (m, 2H), 10.08(s, 1H), 12.45(s, 2H). ^{13}C NMR (125 MHz, CD_3CN, 298 K) δ = 153.12, 133.39, 131.25, 130.99, 130.76, 129.40, 128.94, 128.82, 128.64, 128.41, 128.01, 127.30, 71.00, 35.35, 31.45, 27.22. H-MS (ESI): calcd for [**M** - BF_4] $^+$, [$C_{46}H_{43}N_2O$] $^+$, m/z =639.3370, found m/z = 639.3375.

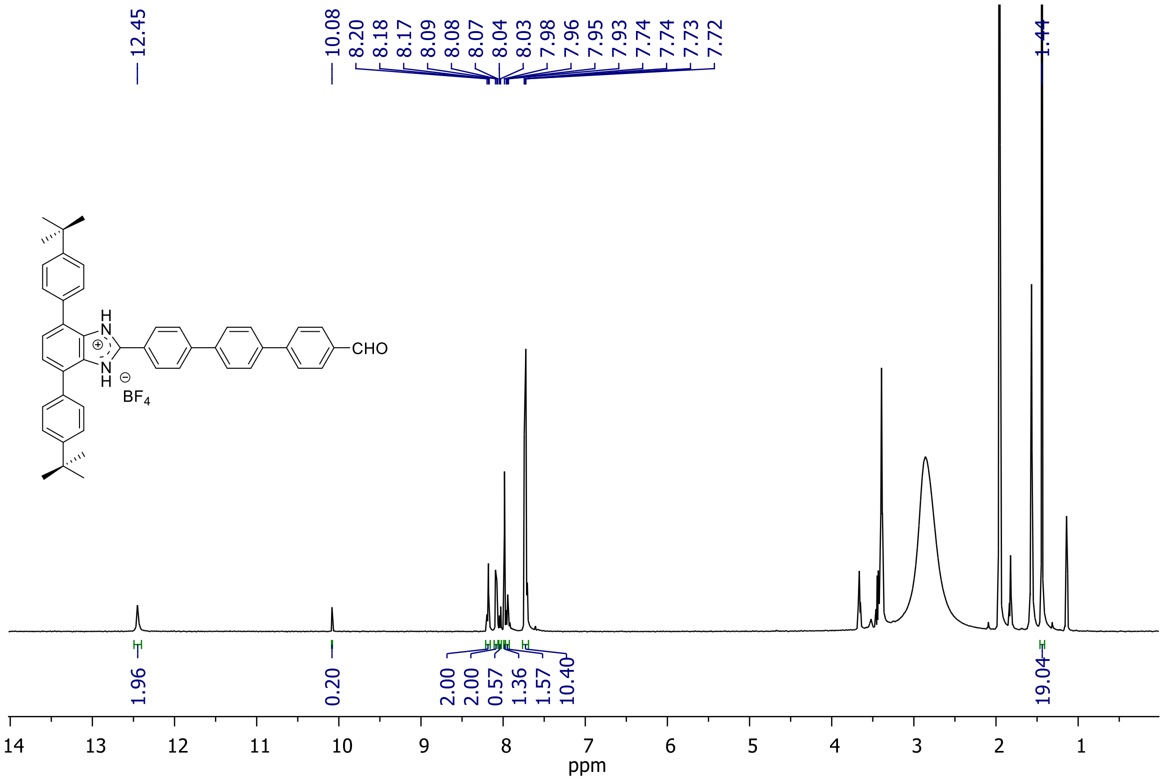

Figure S13. ^{1}H NMR spectrum of [**4**-H][BF_4]; THF and Et_2O (500 MHz, CD_3CN, 298 K).

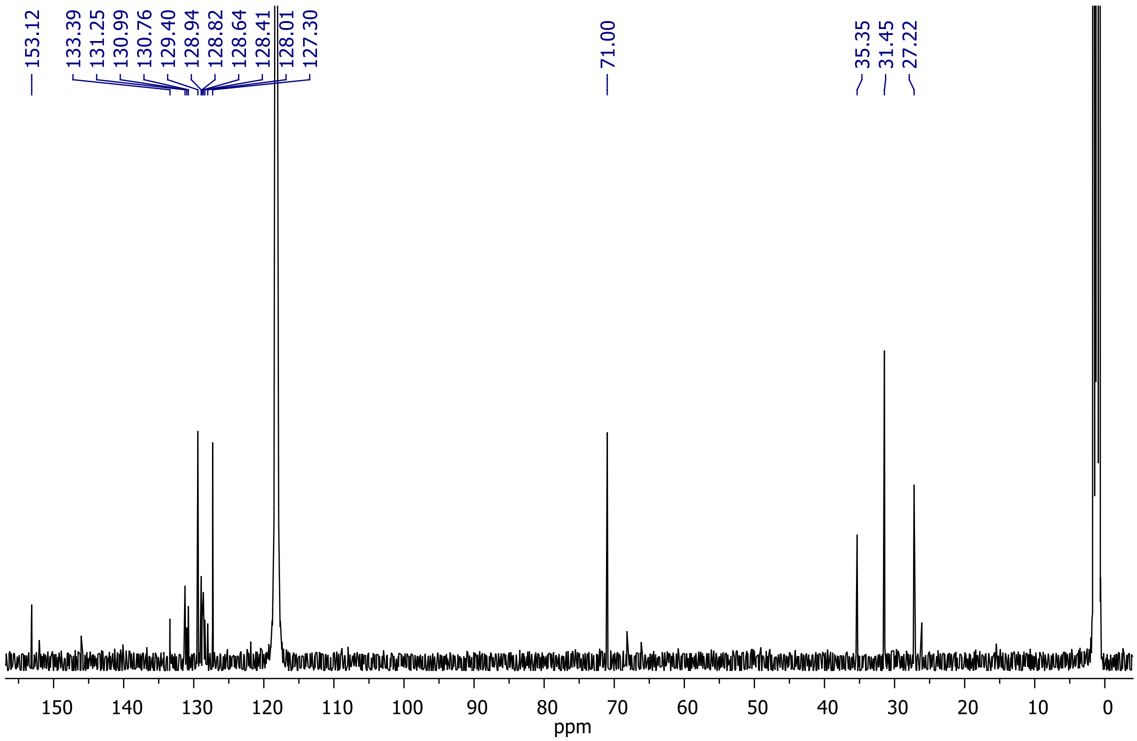

Figure S14. ^{13}C NMR spectrum of [**4**-H][BF_4] (125 MHz, CD_3CN, 298 K).

Synthesis of R_3

DB24C8 (0.25 g, 0.56 mmol) was added to [**4**-H][BF_4] (90 mg, 0.14 mmol) in a round bottom flask. $CHCl_3$ (20 mL) was added and the mixture stirred at room temperature until a clear solution formed. 1,2-Diamino-3,6-di(4-t-butylphenyl)benzene **1** (52.4 mg, 0.14 mmol) was added, followed by $ZrCl_4$ (32.6 mg, 0.014 mmol) and the yellow solution was stirred at room temperature for 24 h. The solution was filtered, solvents were evaporated and CH_3CN (20 mL) was added to dissolve the solids. Et_3N (0.2 mL) was slowly added and formation of a solid was observed. The solution was filtered and air dried producing a white solid. Yield 50% (110 mg). Mp: 230-235°C. ^{1}H NMR (500 MHz, CD_2Cl_2, 298 K) δ = 1.31 (s, 36H), 2.64(s, 8H), 3.48(s, 8H), 4.03(s, 8H), 6.93(m, 4H), 7.01(m, 4H), 7.32 (m, 10H), 7.55(d, 2H, *J*= 7.7), 7.59 (d, 4H, *J*= 8.2), 7.79(d, 8H, *J*= 7.8), 8.26 (d, 4H, *J*= 8.2), 8.7(d, 4H, *J*= 8.1), 10.77(s, 2H). ^{13}C NMR (125 MHz, CD_2Cl_2, 298 K) δ = 30.82, 30.92, 34.19, 68.00, 68.27, 68.74, 111.48, 120.63, 120.92, 122.79, 124.92, 125.61, 126.16, 127.00, 127.56, 128.72, 128.97, 129.14, 130.24, 135.31, 135.54, 139.27, 140.22, 148.25, 149.85,150.12, 151.86. HR-MS (ESI): calcd for $[M+H]^+$, $[C_{96}H_{103}N_4O_8]^+$, *m/z* =1439.7770, found *m/z* = 1439.7786.

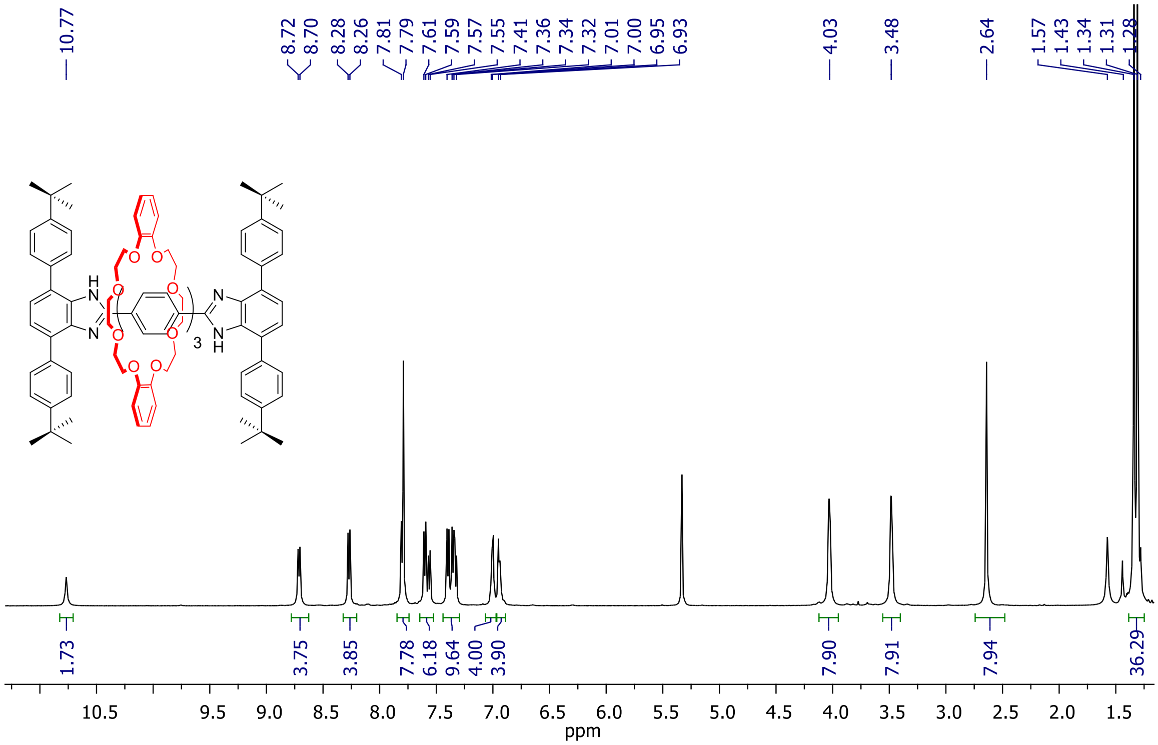

Figure S15. ^{1}H NMR spectrum of **R_3** (500 MHz, CD_2Cl_2, 298 K).

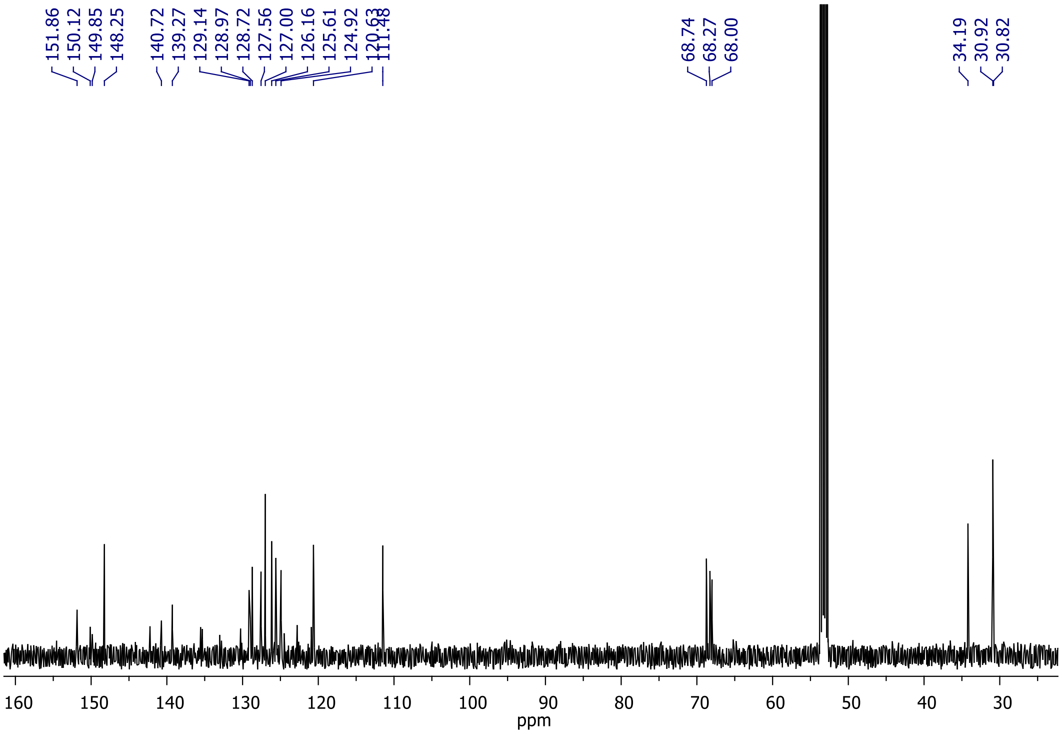

Figure S16. ^{13}C NMR spectrum of **R_3** (125 MHz, CD_2Cl_2, 298 K).

Synthesis of [R_3-H_2][BF_4]$_2$

Tetrafluoroboric acid was added dropwise to **R_3** which was dissolved in DCM. After 30 min, the DCM was removed and diethyl ether was added. The solid was filtered and recrystallized from CH_3CN. Yield 70% (12mg). Mp: >300°C. ^{1}H NMR (500 MHz, CD_3CN, 298 K) δ = 1.44 (d, 36H), 2.96 (dd, 4H), 3.15 (m, 4H), 3.34 (m, 8H), 3.55 (m, 8H), 6.34 (m, 4H), 6.63 (m, 4H), 7.08 (s, 2H), 7.44 (d, 4H, *J*= 8.35 Hz), 7.64 (d, 4H, *J*= 8.35 Hz), 7.72 (m, 12H), 8.01 (m, 4H), 8.11 (m, 4H), 8.18 (d, 2H, *J*= 8.5 Hz), 8.53 (d, 2H, *J*= 8.4 Hz), 12.33 (s, 2H), 12.91 (s, 2H). ^{13}C NMR (125 MHz, CD_3CN, 298 K) δ = 31.41, 35.26, 68.56, 70.44, 70.65, 113.19, 121.50, 121.76, 125.30, 125.49, 126.57, 126.95, 127.20, 127.87, 128.33, 128.50, 128.68, 128.81, 129.32, 129.45, 130.00, 130.73, 131.16, 132.93, 133.37, 139.57, 144.11, 148.05, 151.93, 152.05, 152.77, 153.01. HR-MS (ESI): calcd for $[M-(BF_4)_2]^{2+}$, $[C_{96}H_{104}N_4O_8]^{2+}$, *m/z* =1440.7843, found *m/z* = 1440.7831.

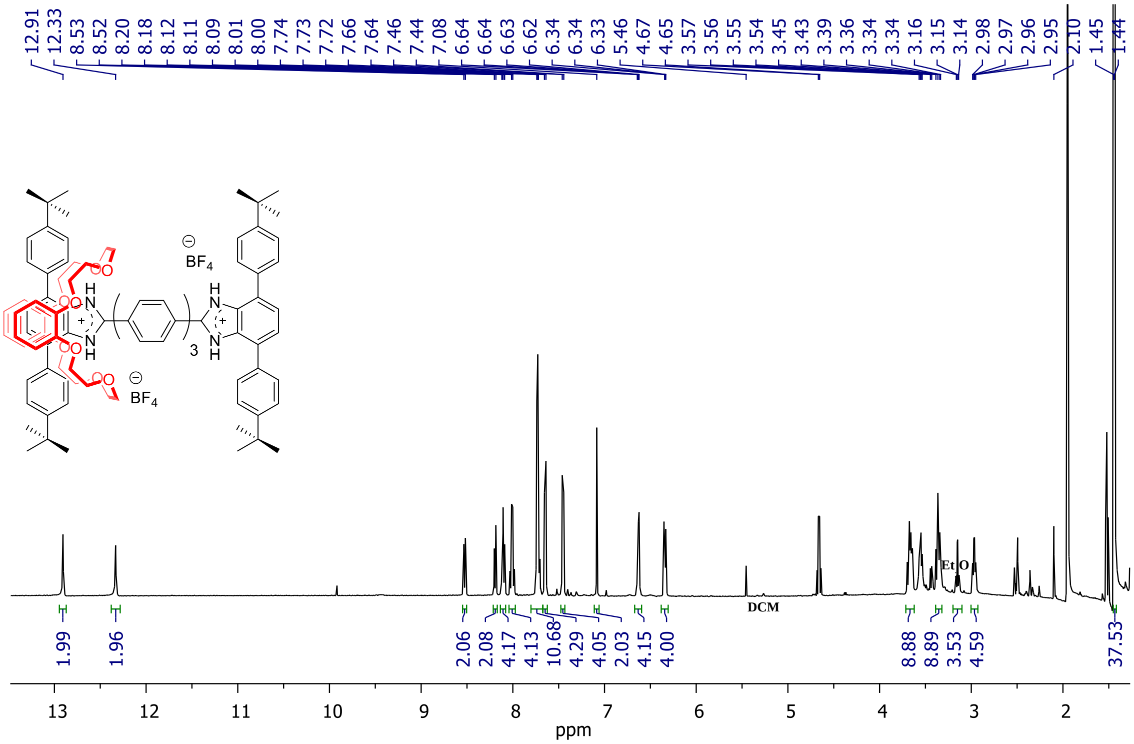

Figure S17. ^{1}H NMR spectrum of [**R**$_3$-H_2][BF_4]$_2$ (500 MHz, CD_3CN, 298 K).

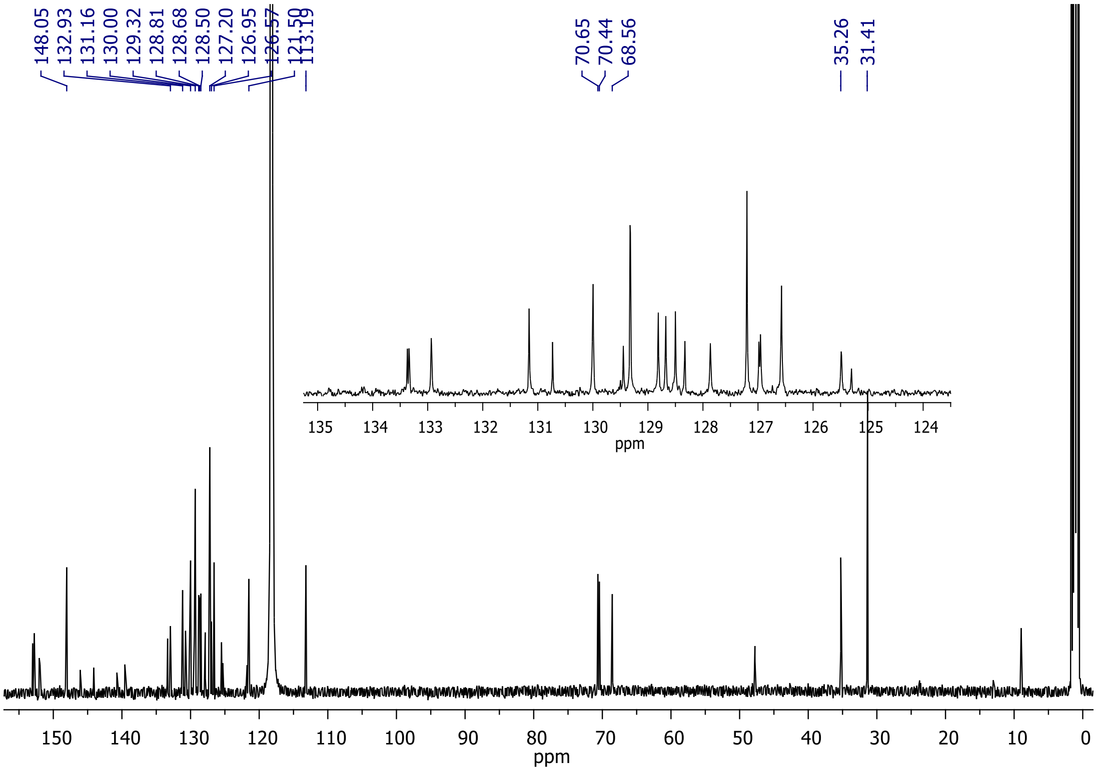

Figure S18. ^{13}C NMR spectrum of [**R**$_3$-H_2][BF_4]$_2$ (125 MHz, CD_3CN, 298 K).

Synthesis of 5

1 (300 mg, 0.805mmol) and 4′-bromo-4-triphenylbenzaldehyde (271mg, 0.805 mmol) were dissolved in $CHCl_3$. $ZrCl_4$ (18.7 mg, 0.08mmol) was added. After 24 h stirring, the solution was filtered and solvent removed. The crude product was purified by recrystallization with ethyl acetate. Yield 90% (500 mg). Mp: 238-240°C. ^{1}H NMR (500 MHz, $CDCl_3$, 298 K) δ =1.42 (s, 18H), 7.45 (s, 2H), 7.52 (d, 2H, *J*= 7.5 Hz), 7.58 (d, 2H, *J*= 7.5 Hz), 7.65 (d, 3H *J*= 7.6 Hz),7.72 (dd, 4H *J*= 7.7 Hz), 7.8 (s,3H), 8.19 (d, 2H, *J*= 8.1 Hz), 10.01 (s, 1H). ^{13}C NMR (125 MHz, $CDCl_3$, 298 K) δ = 31.50, 34.81, 121.91, 123.29, 126.09, 127.35, 127.47, 127.65, 128.40, 128.71, 132.09, 135.16, 139.47, 139.61, 142.37, 150.85, 150.99. H-MS (ESI): calcd for [M+H], $[C_{45}H_{42}BrN_2]^+$, *m/z* =689.2526, found *m/z* = 689.2531.

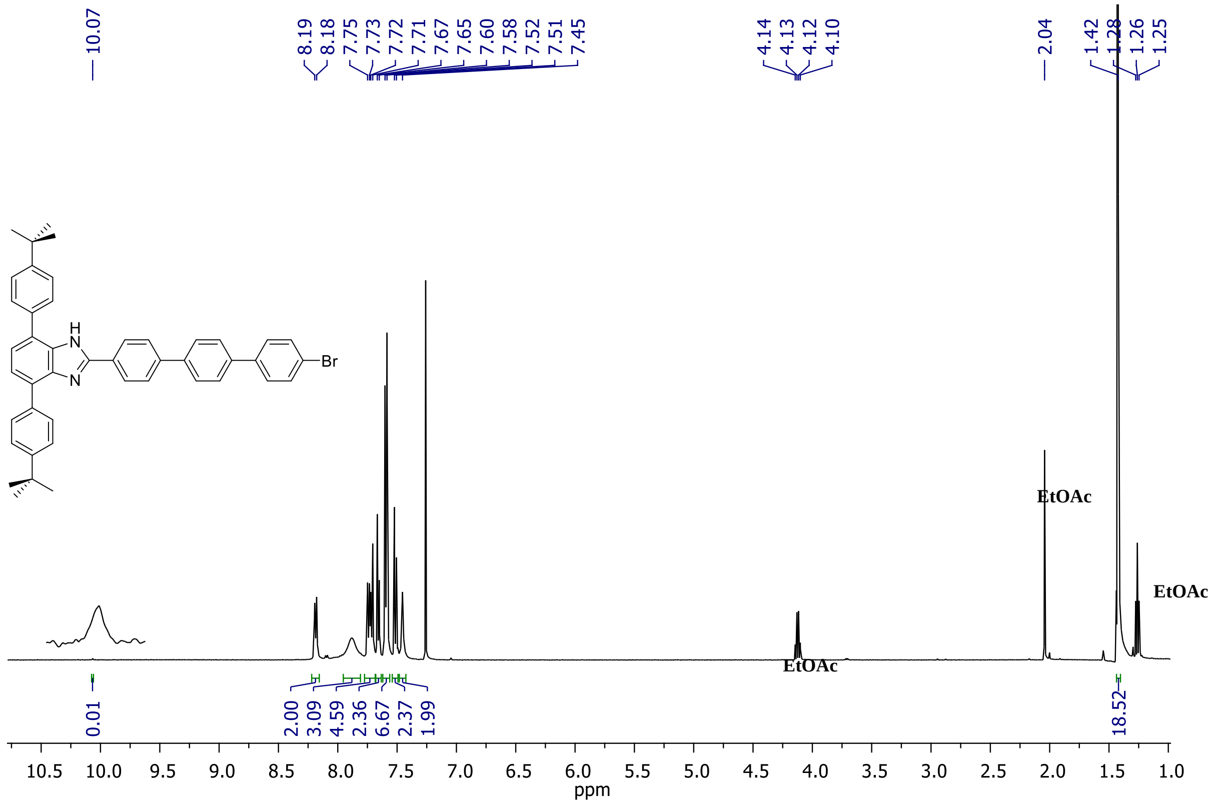

Figure S19. ^{1}H NMR spectrum of **5** (500 MHz, $CDCl_3$, 298 K).

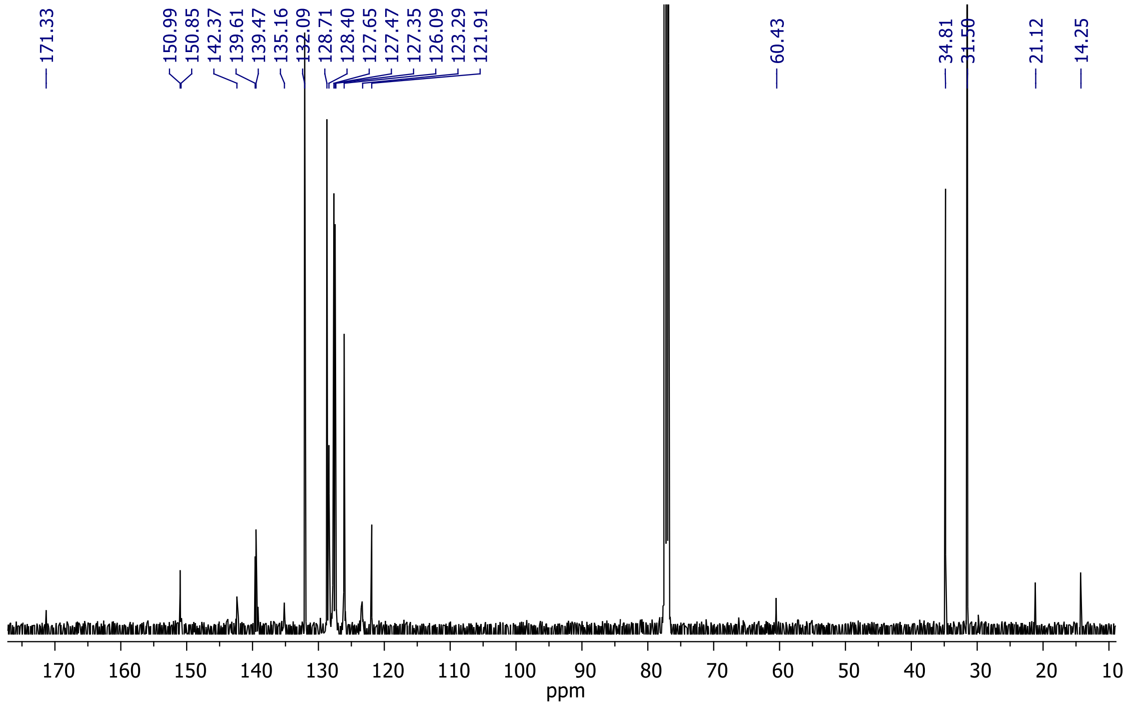

Figure S20. ^{13}C NMR spectrum of **5** (125 MHz, $CDCl_3$, 298 K).

Synthesis of 5b

5 (300 mg, 0.43 mmol), 4-formylphenylboronic acid (65.2 mg, 0.43 mmol) and [Pd(PPh_3)$_4$] (34 mg, 0.0294 mmol) were added to a 100 mL Schlenk flask, degassed and backfilled with N_2. A solution of 2M Na_2CO_3 (100 mL) and THF (100 mL) were added and the yellow solution refluxed (80 °C) for 24 h. The solution was cooled to room temperature, the organic layer was extracted with DCM (2 x 50 mL) and the solution was dried over anhydrous $MgSO_4$. The product was recrystallized from acetonitrile to give a yellow solid, Yield 85% (260 mg). Mp: 185-190, ^{1}H NMR (500 MHz, CD_2Cl_2, 298 K) δ =1.42 (s, 18H), 7.39 (d, 1H, *J*= 7.7 Hz), 7.52 (d, 1H, *J*= 7.7 Hz), 7.58 (d, 2H, *J*= 8.4 Hz), 7.64 (dd, 4H *J*= 8.4 Hz),7.80 (d, 4H *J*= 8.6 Hz), 7.81 (s,4H), 7.85 (dd, 4H, *J*= 6.5 Hz), 7.97 (d, 2H, *J*= 8.3 Hz), 8.08 (d, 2H, *J*= 8.4 Hz), 8.18 (d, 2H, *J*= 8.4 Hz), 9.7 (s, 1H), 10.06 (s, 1H). ^{13}C NMR (125 MHz, CD_2Cl_2 298K) δ =192.04, 150.71, 135.89, 130.50, 129.26, 128.13, 127.82, 127.79, 127.71, 127.39, 126.79, 125.63, 124.80, 123.38, 122.28, 70.78, 34.94, 31.42. HR-MS (ESI): calcd for $[M+H]^+$, $[C_{52}H_{47}N_2O]^+$, m/z =715.3683, found m/z = 715.3688.

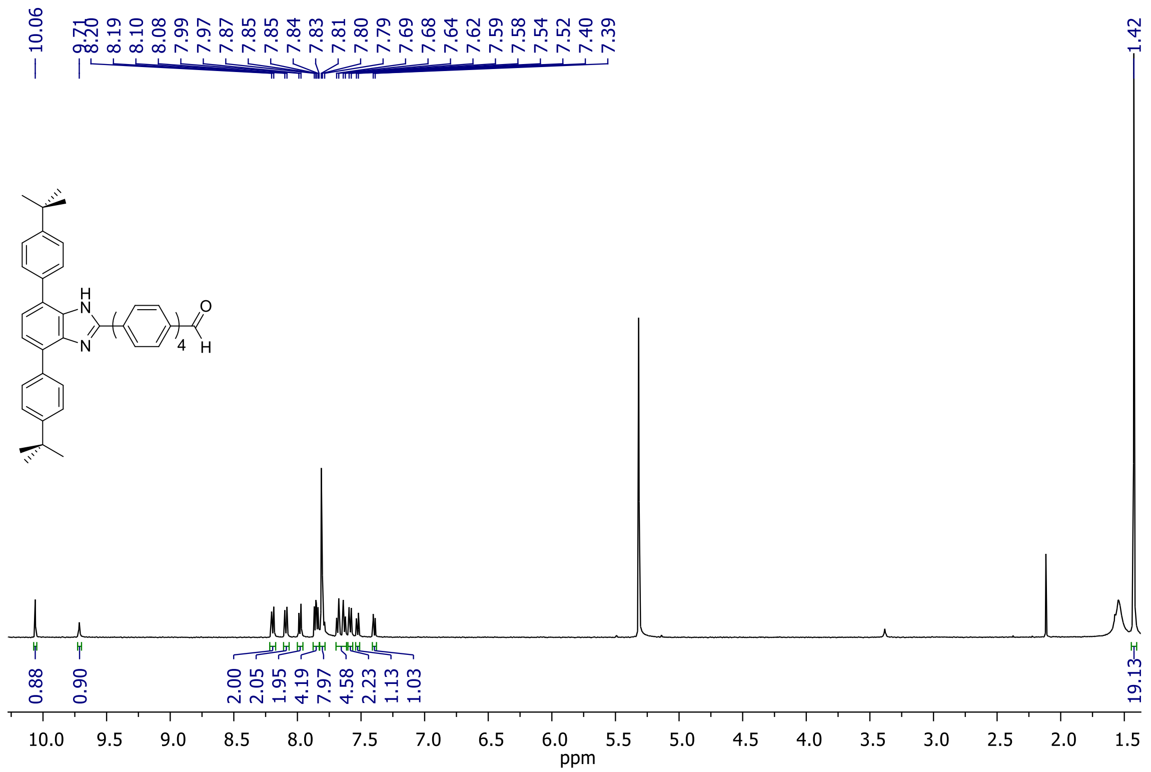

Figure S21. ^{1}H NMR spectrum of **5b** (500 MHz, CD_2Cl_2, 298 K).

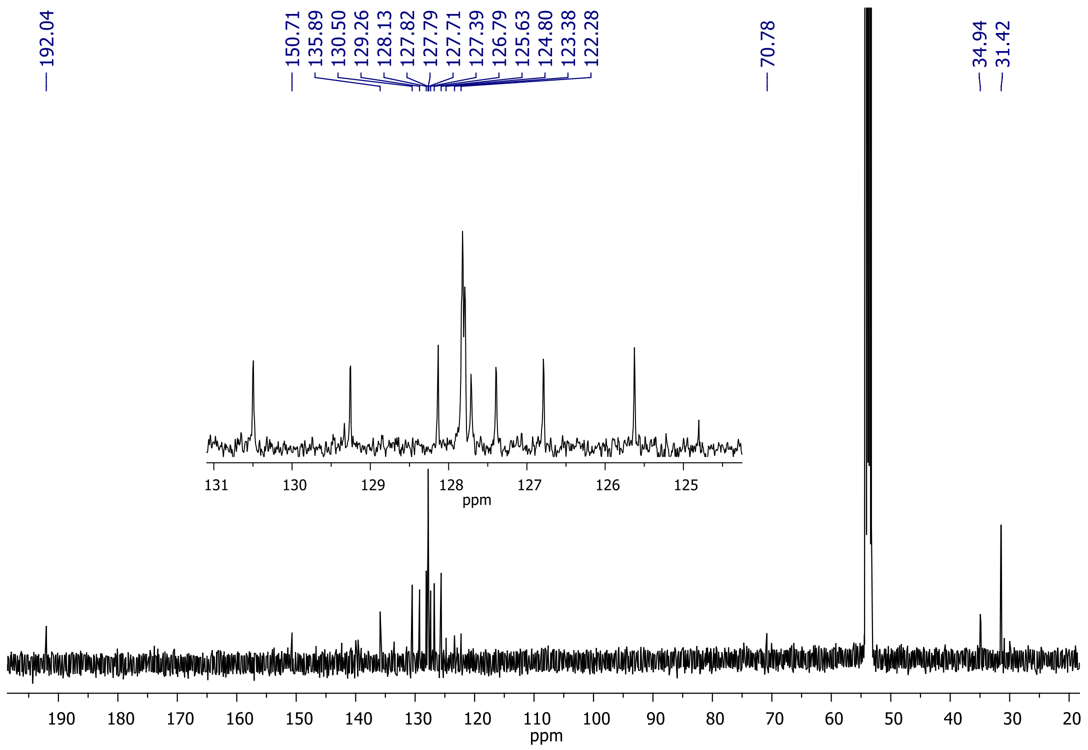

Figure S22. ^{13}C NMR spectrum of **5b** (125 MHz, CD_2Cl_2 298K).

Synthesis of [5b-H][BF$_4$]

Tetrafluoroboric acid was added dropwise to **5b** which was dissolved in DCM. After 30 min, the DCM was removed and diethyl ether was added. The solid was filtered and recrystallized from CH_3CN. Yield 75% (150 mg) Mp: 235-240. ^{1}H NMR (500 MHz, CD_3CN, 298 K) δ =1.45 (s, 18H) 7.73 (m, 10H), 7.8 (d, 4H, J= 7.8 Hz), 7.93 (d, 4H J= 8.4 Hz), 7.96 (d, 2H J= 8.3 Hz), 8.01 (d, 2H J= 8.4 Hz), 8.06 (d, 2H, J= 8.6 Hz), 8.1 (d, 2H, J= 8.5 Hz), 10.08 (s, 1H), 12.33 (s, 2H). ^{13}C NMR (125 MHz, CD_3CN, 298 K) δ = 193.21, 153.23, 152.19, 146.79, 146.50, 141.38, 140.87, 139.78, 139.00, 136.61, 133.49, 131.30, 131.07, 130.83, 129.53, 128.85, 128.82, 128.60, 128.52, 128.46, 128.10, 127.43, 121.59, 35.47, 31.58. HR-MS (ESI): calcd for $[M\text{-}BF_4]^+$, $[C_{52}H_{47}N_2O]^+$, m/z = 715.3683, found m/z = 715.3688.

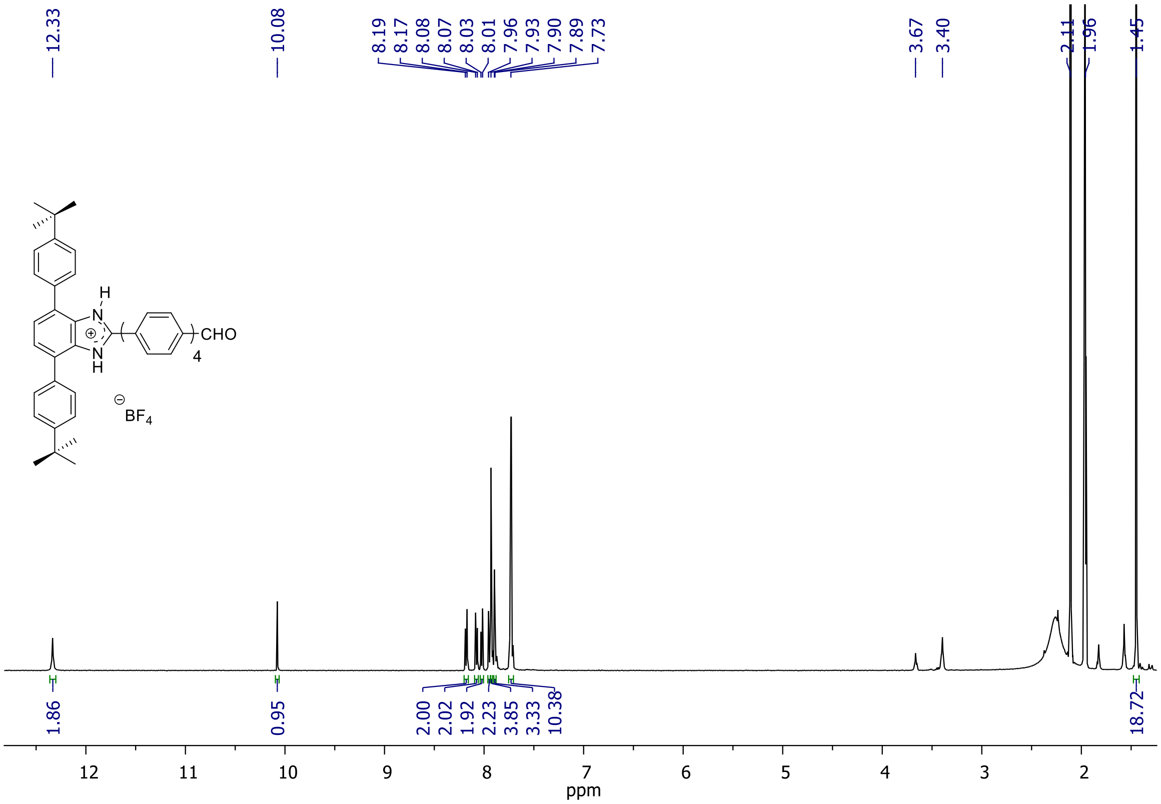

Figure S23. ^{1}H NMR spectrum of [**5b**-H][BF_4] (500 MHz, CD_3CN, 298 K).

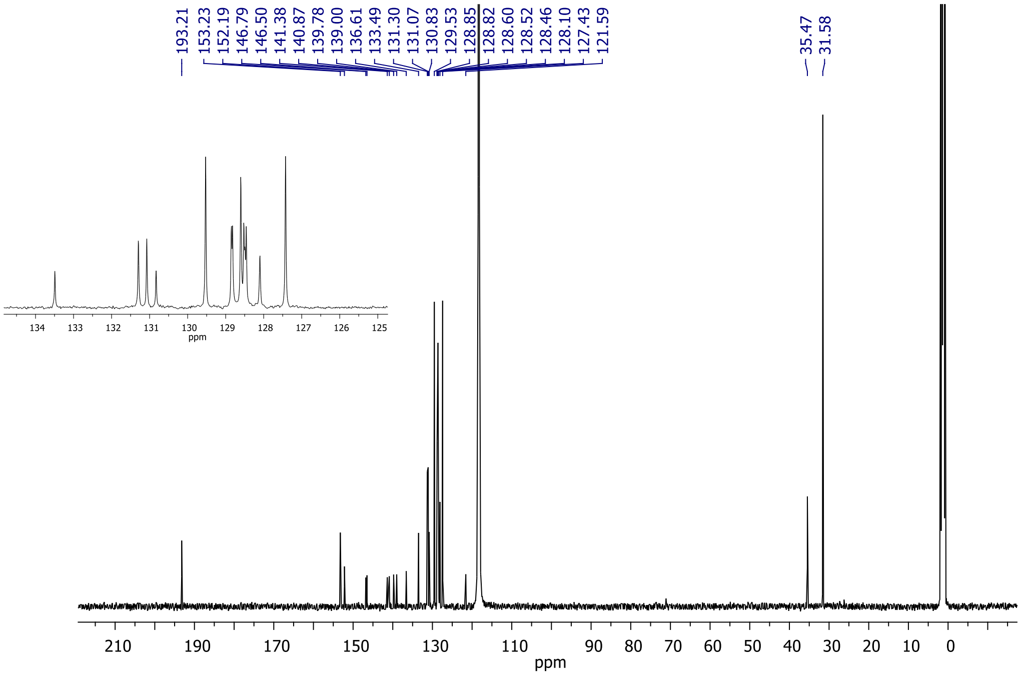

Figure S24. ^{13}C NMR spectrum of [**5b**-H][BF_4] (125 MHz, CD_3CN, 298 K).

Synthesis of R_4

DB24C8 (0.25 g, 0.56 mmol) was added to [**5b**-H][BF_4] (90 mg, 0.14 mmol) in a round bottom flask. $CHCl_3$ (20 mL) was added and the mixture stirred at room temperature until a clear solution formed. 1,2-Diamino-3,6-di(4-t-butylphenyl)benzene **1** (52.4 mg, 0.14 mmol) was added, followed by $ZrCl_4$ (32.6 mg, 0.014 mmol) and the yellow solution was stirred at room temperature for 24 h. The solution was filtered, solvents were evaporated and CH_3CN (20 mL) was added to dissolve the solids. Et_3N (0.2 mL) was slowly added and formation of a solid was observed. The solution was filtered and air dried producing a white solid. Yield 60% (120mg). Mp: >300°C. ^{1}H NMR (500 MHz, CD_2Cl_2, 298 K) δ = 1.32 (36H), 2.62 (s, 8H), 3.46 (s,8H), 4.01 (s, 8H), 6.92 (m, 4H), 6.98 (m, 4H), 7.30 (dd,6H *J*= 7.7 Hz), 7.37 (d, 4H, *J*= 8.4Hz), 7.53 (d, 2H, *J*= 7.7 Hz), 7.57 (d, 4H, *J*= 8.3 Hz), 7.76 (m, 12H), 8.24 (d, 4H, *J*= 8.4Hz), 8.69 (d, 4H, *J*= 8.1 Hz), 10.76 (s, 2H). ^{13}C NMR (125 MHz, CD_2Cl_2, 298 K) δ = 30.13, 31.03, 31.47, 31.57, 34.85, 53.43, 53.65, 53.86, 54.08, 54.30, 68.66, 68.94, 69.41, 112.15, 121.29, 121.58, 123.45, 125.17, 125.58, 126.27, 126.84, 127.68, 127.73, 128.22, 129.39, 129.83, 130.93, 135.97, 136.21, 139.87, 140.00, 142.89, 148.92, 150.52, 152.53. HR-MS (ESI): calcd for $[M+H]^+$, $[C_{102}H_{107}N_4O_8]^+$, *m/z* =1516.8117, found *m/z* = 1516.8123.

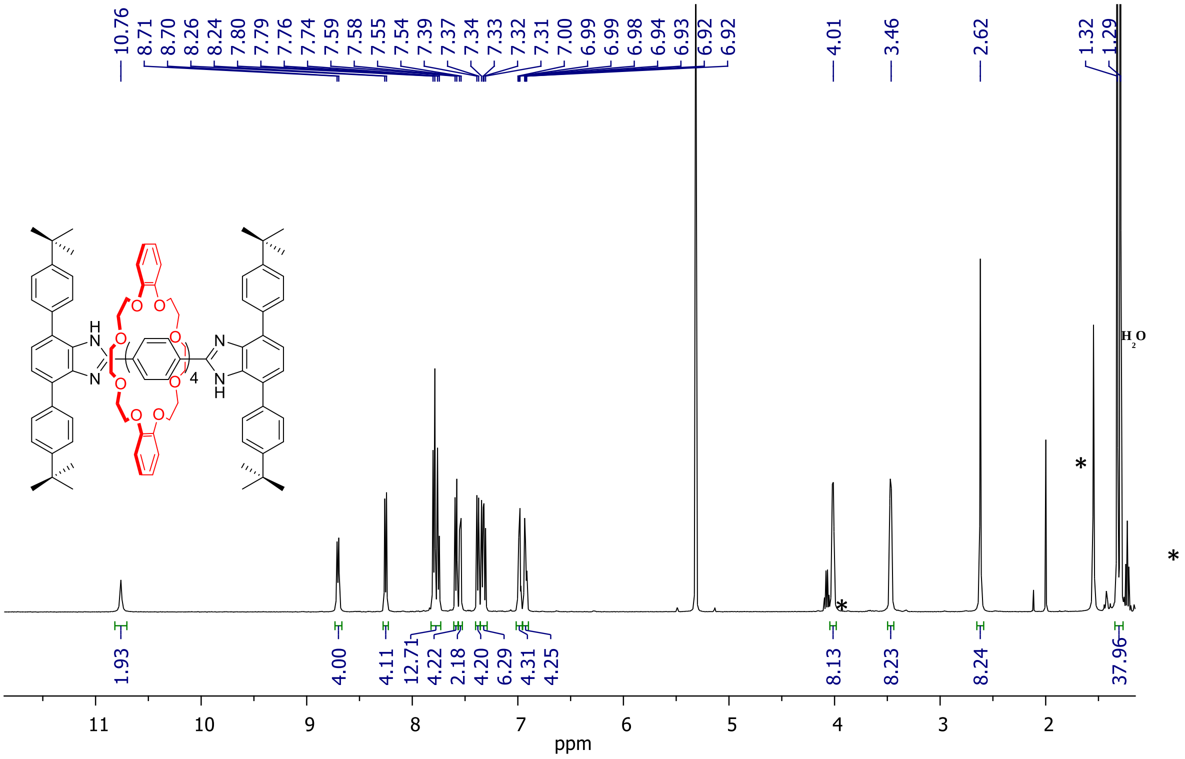

Figure S25. ^{1}H NMR spectrum of **R₄** (* = ethyl acetate) (500 MHz, CD_2Cl_2, 298 K).

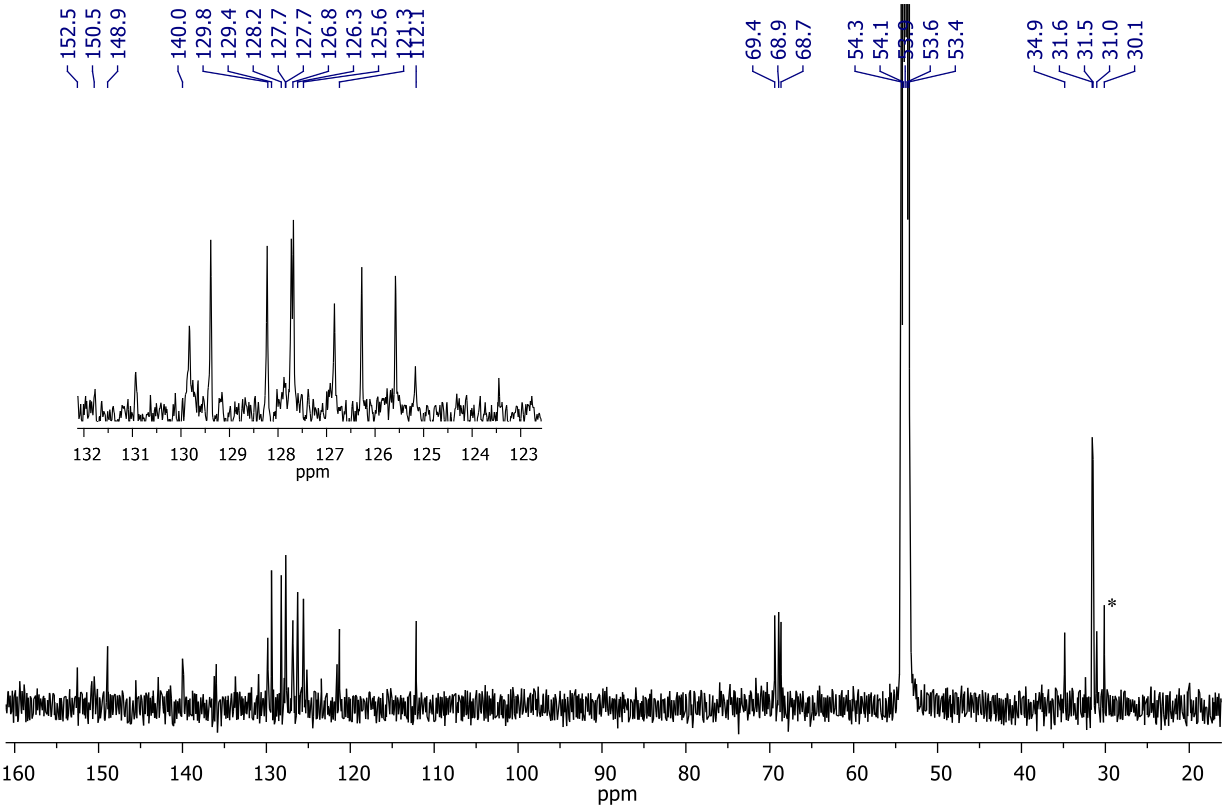

Figure S26. ^{13}C NMR spectrum of **R₄** (* = grease) (125 MHz, CD_2Cl_2, 298 K).

Synthesis of $[R_4-H_2][BF_4]_2$

Tetrafluoroboric acid was added dropwise to **R_4** which was dissolved in DCM. After 30 min, the DCM was removed and diethyl ether was added. The solid was filtered and recrystallized from CH_3CN. Yield 80% (13.5mg). Mp >300°C. ^1H NMR (500 MHz, CD_3CN, 298 K) δ =1.43 (36H), 2.95 (m, 4H), 3.33 (m,8H), 3.55 (m, 4H), 3.65 (m, 8H), 6.32 (m, 4H), 6.61 (m,4H), 7.07 (s, 2H), 7.43 (d, 4H, J = 8.3 Hz), 7.62 (d, 4H, J = 8.3 Hz), 7.69 (m, 12H), 7.92 (m, 8H), 8.06 (m, 4H), 8.16 (d, 2H, J = 8.5 Hz), 8.51 (d, 2H, J = 8.3 Hz), 12.29 (s, 2H), 12.90 (s, 2H). ^{13}C NMR (125 MHz, CD_3CN, 298 K) δ = 31.47, 35.34, 68.65, 70.54, 70.75, 113.28, 121.59, 125.55, 126.67, 126.96, 127.07, 127.33, 128.00, 128.40, 128.55, 128.59, 128.78, 129.43, 129.54, 130.11, 130.75, 131.25, 133.01, 133.40, 133.48, 139.03, 140.75, 146.42, 148.14, 152.12, 152.86, 153.12. 31.4. HR-MS (ESI): calcd for $[M-(BF_4)_2]^{2+}$, $[C_{102}H_{108}N_4O_8]^{2+}$, m/z =1516.8156, found m/z = 1516.8153.

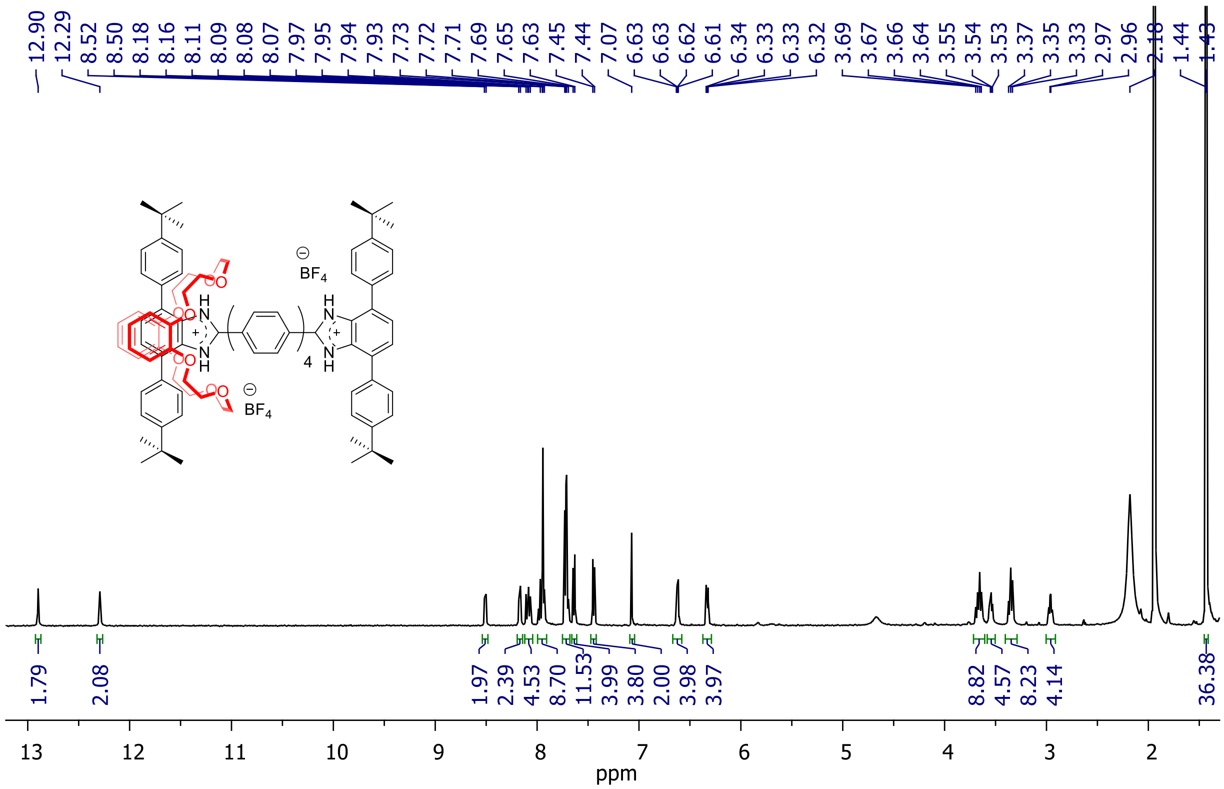

Figure S27. ^{1}H NMR spectrum of [**R_4**-H_2][BF_4]$_2$ (500 MHz, CD_3CN, 298 K).

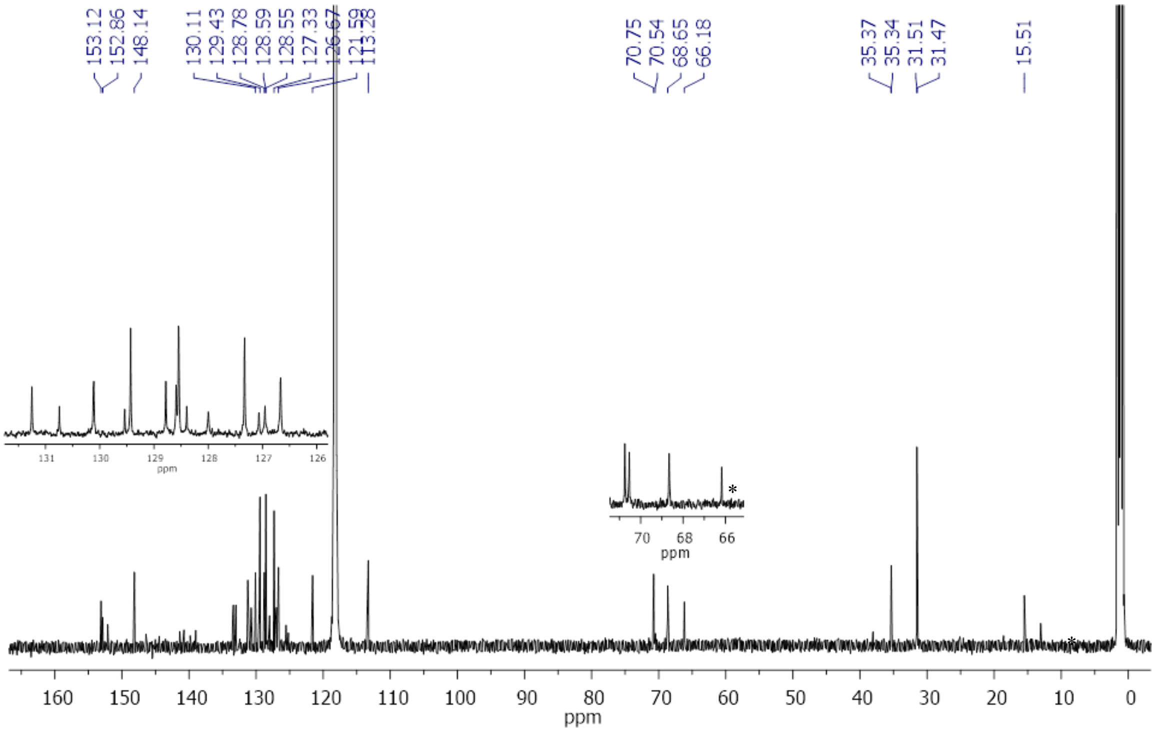

Figure S28. ^{13}C NMR spectrum of [**R_4**-H_2][BF_4]$_2$ (* = Et_2O) (125 MHz, CD_3CN, 298 K).

Synthesis of 6

1,2-Diamino-3,6-di(4′-*t*-butylphenyl)benzene (130 mg, 0.36 mmol), naphthalene-2,6-dicarbaldehyde (330 mg, 1.8 mmol), and $ZrCl_4$ (4.2 mg, 0.018 mmol) were added to 50 mL of CH_3CN. The mixture was stirred in open air at room temperature for 24 h. Trimethylamine (0.5 mL) and chloroform (20 mL) were added and the mixture was filtered. The solvents of the filtrate were removed on a rotary evaporator. The residue was purified by column chromatography using dichloromethane as eluent. Further washing with acetonitrile gave the pure product as a pale yellow solid. Yield: 162 mg, 84%. Mp: 280-283 °C. ^{1}H NMR (300 MHz, DMSO-d_6, 298 K) δ = 12.93 (s, 1H), 10.18 (s, 1H), 9.03 (s, 1H), 8.64 (s, 1H), 8.58 (d, 1H, J = 8.7 Hz), 8.30 (d, 1H, J = 8.7 Hz), 8.18 (d, 1H, J = 8.7 Hz), 8.14 (d, 2H, J = 8.4 Hz), 7.96 (d, 1H, J = 8.7 Hz), 7.70 (d, 2H, J = 8.4 Hz), 7.60 (d, 2H, J = 8.4 Hz), 7.57 (d, 2H, J = 8.4 Hz), 7.50 (d, 1H, J = 8.4 Hz), 7.31 (d, 1H, J = 8.4 Hz),1.39 (s, 9H),1.37 (s, 9H). ^{13}C NMR (75 MHz, DMSO-d_6, 298 K) δ = 193.5, 152.2, 150.5, 150.1, 142.7, 136.3, 135.9, 135.6, 135.0, 134.7, 134.3, 133.3, 131.1, 130.4, 130.1, 129.2, 128.7, 127.2, 126.7, 126.3, 125.7, 125.5, 124.2, 123.7, 121.8, 31.7, 31.7. HR-MS (ESI): calcd for $[M+H]^+$, $[C_{38}H_{37}N_2O]^+$, m/z = 537.2861, found m/z = 537.2907.

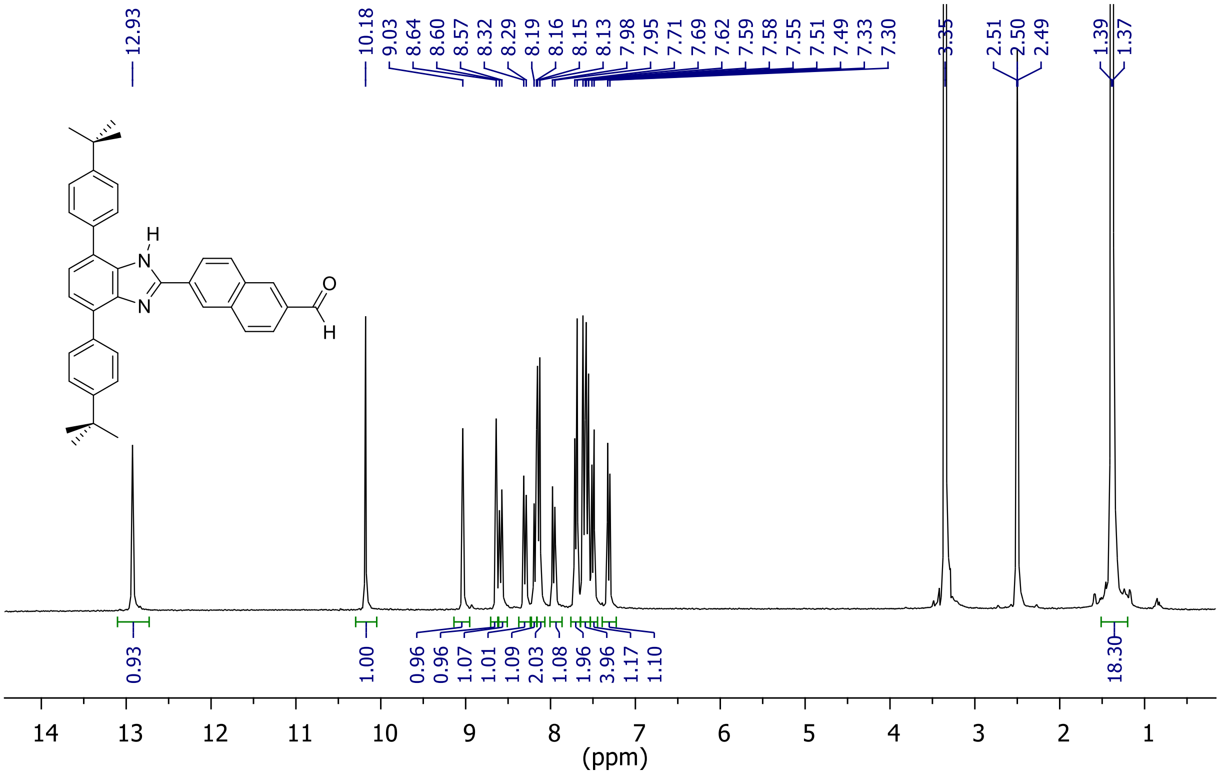

Figure S29. ^{1}H NMR spectrum of **6** (300 MHz, DMSO-d_6, 298 K).

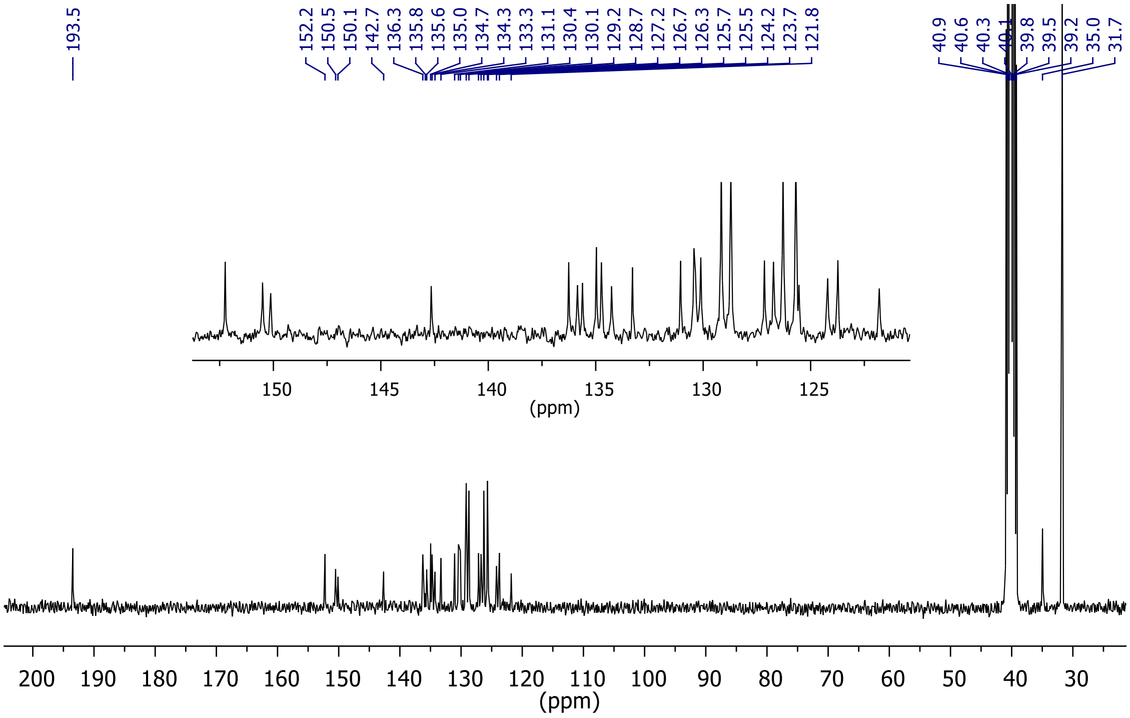

Figure S30. ^{13}C NMR spectrum of **6** (75 MHz, DMSO-d_6, 298 K).

Synthesis of [6-H][BF_4]

Tetrafluoroboric acid diethyl ether complex (36 μL, 0.26 mmol) was added to a mixture of **6** (130 mg, 0.24 mmol) in diethyl ether (100 mL) with stirring. After stirred for 10 min, the resulting white solid was filtered, rinsed with diethyl ether and air dried. Yield: 141 mg, 93%. Mp 240–243 °C. ^{1}H NMR (500 MHz, CD_3CN, 298 K) δ = 12.45 (br s, 2H), 10.21 (s, 1H), 8.76 (s, 1H), 8.60 (s, 1H), 8.38 (d, 1H, J = 8.5 Hz), 8.23 (d, 1H, J = 8.5 Hz), 8.15 (d, 1H, J = 8.5 Hz), 8.08 (d, 1H, J = 8.5 Hz), 7.72 (s, 2H), 7.70 (m, 8H), 1.42 (s, 18H). ^{13}C NMR (125 MHz, CD_3CN, 298 K) δ = 192.4, 152.3, 150.9, 136.6, 135.1, 134.75, 133.8, 132.4, 131.4, 130.9, 130.2, 130.0, 128.6, 127.6, 127.3, 126.5, 125.9, 124.5, 121.7, 34.5, 30.6. HR-MS (ESI): calcd for $[M-BF_4]^+$, $[C_{38}H_{37}N_2O]^+$, m/z = 537.2900, found m/z = 537.2908.

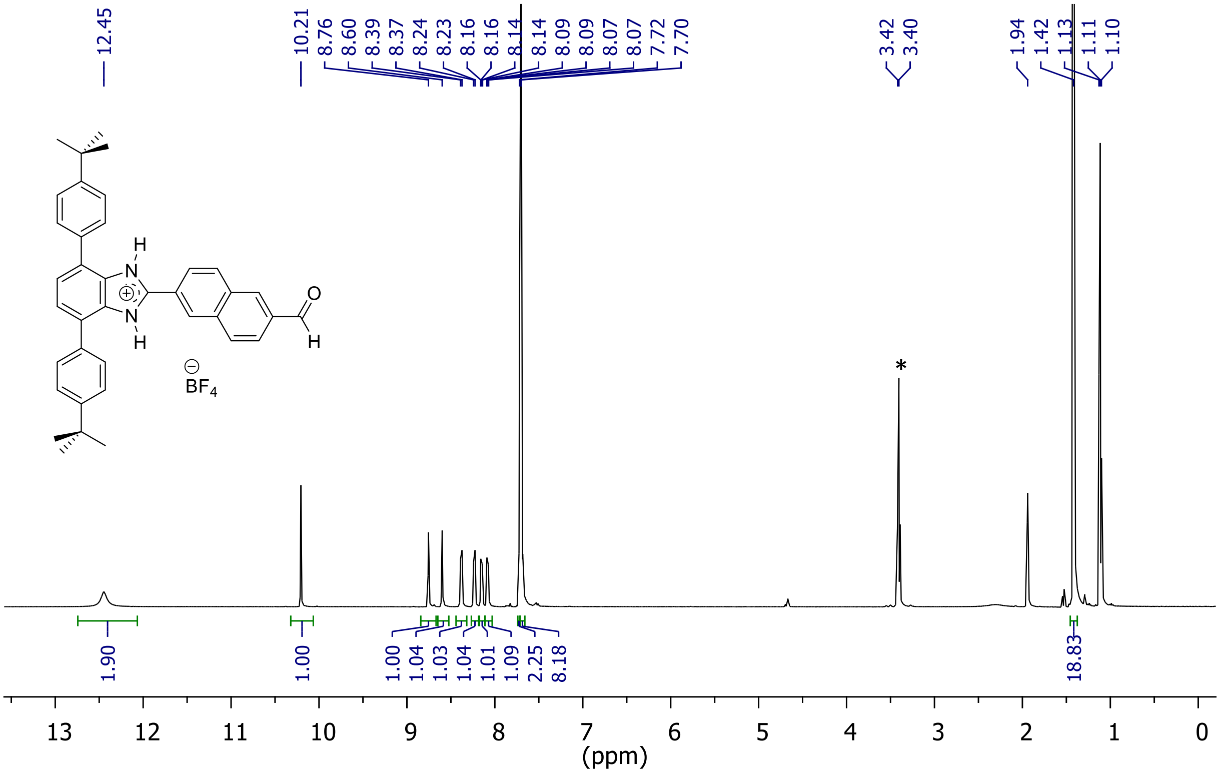

Figure S31. ^{1}H NMR spectrum of [**6**-H][BF_4] (* = diethyl ether) (500 MHz, CD_3CN, 298 K).

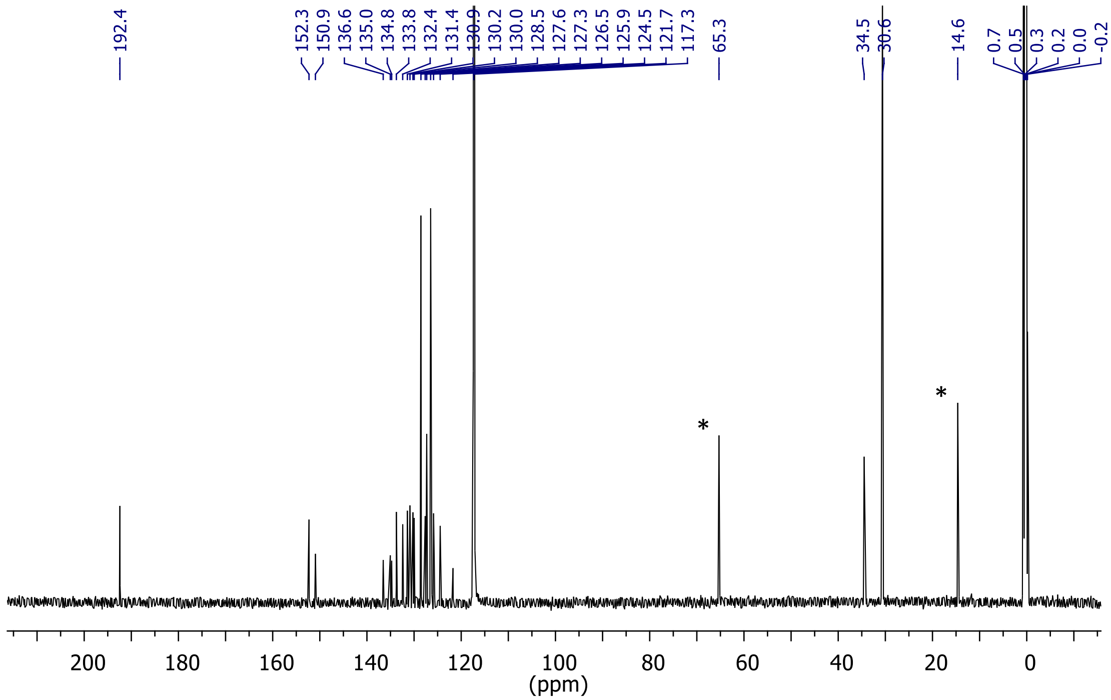

Figure S32. ^{13}C NMR spectrum of [**6**-H][BF_4] (* = diethyl ether) (125 MHz, CD_3CN, 298 K).

Synthesis of R_5

[6-H][BF_4] (94 mg, 0.15 mmol) and **DB24C8** (135 mg, 0.30 mmol) were stirred in $CHCl_3$ (20 mL) until a clear solution resulted. 1,2-Diamino-3,6-di(4′-*t*-butylphenyl)benzene (57 mg, 0.154 mmol) was added followed by $ZrCl_4$ (3.6 mg, 0.015 mmol). The mixture was stirred at room temperature overnight, filtered and the filtrate was concentrated under vacuum. The residue was washed with diethyl ether twice and air dried. The dried solid was dissolved in CH_3CN (30 mL) and filtered. Et_3N (0.2 mL) was added to the filtrate. The mixture was heated to reflux and allowed to cool to room temperature. The pale yellow solid was filtered, washed with CH_3CN, and air dried. Yield: 171 mg, 85%. Mp> 300 °C. ^{1}H NMR (500 MHz, CD_2Cl_2, 298 K) δ = 10.70 (br s, 1H), 9.08 (s, 2H), 8.70 (d, 2H, J = 4.2 Hz), 8.22 (d, 4H, J = 8.5 Hz), 7.81 (d, 2H, J = 4.2 Hz), 7.54 (d, 4H, J = 8.5 Hz), 7.49 (d, 2H, J = 8.4 Hz), 7.32 (m, 10H), 6.96 (m, 8H), 3.97 (s, 8H), 3.36 (s, 8H), 2.44 (s, 8H), 1.25 (s, 36H). ^{13}C NMR (125 MHz, CD_3CN, 298 K) δ = 192.4, 152.3, 150.9, 136.6, 135.1, 134.75, 133.8, 132.4, 131.4, 130.9, 130.2, 130.0, 128.6, 127.6, 127.3, 126.5, 125.9, 124.5, 121.7, 34.5, 30.6. ^{13}C NMR (75 MHz, CD_2Cl_2) δ 152.47, 150.45, 150.13, 148.53, 142.57, 135.77, 135.56, 133.53, 133.42, 130.62, 129.03, 128.64, 128.27, 127.82, 126.99, 125.93, 125.18, 124.77, 123.10, 121.20, 121.03, 111.87, 69.08, 68.55, 68.34, 34.50, 34.45, 31.18, 31.11. HR-MS (ESI): calcd for $[M+H]^+$, $[C_{88}H_{97}N_4O_8]^+$, m/z = 1337.7301, found m/z = 1337.7306.

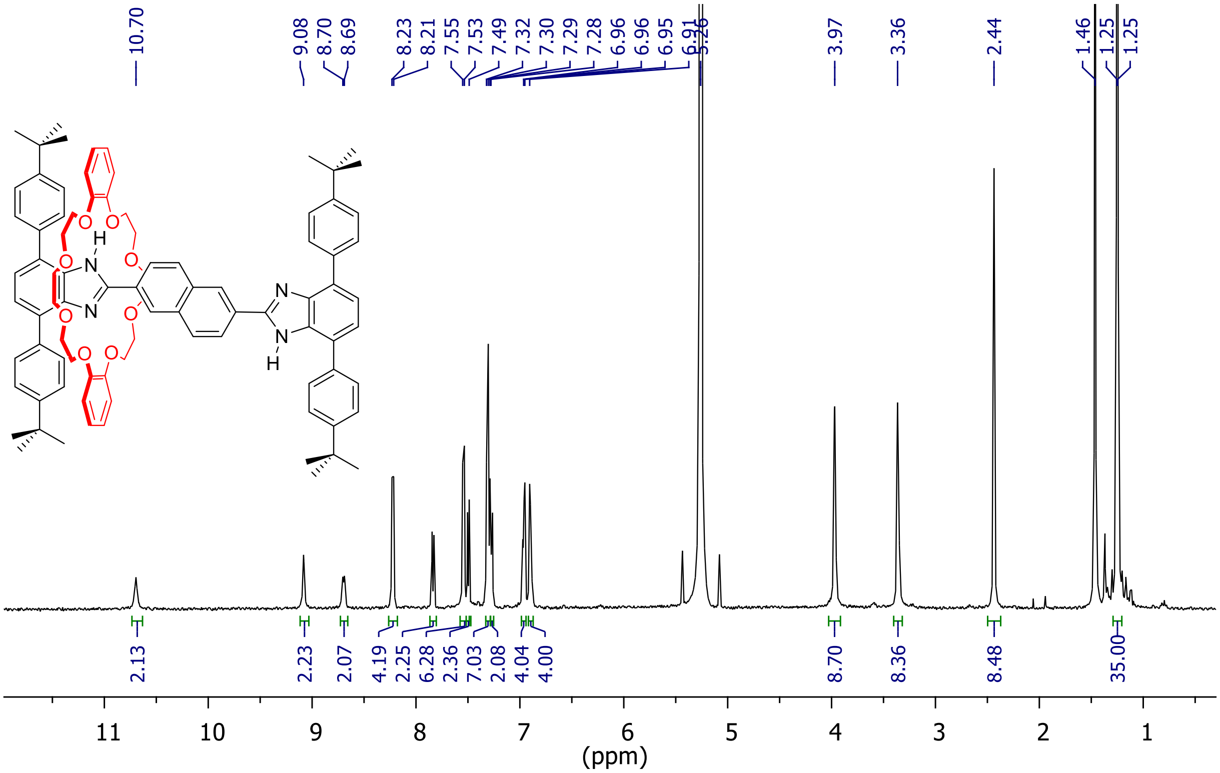

Figure S33. ^{1}H NMR spectrum of **R_5** (500 MHz, CD_2Cl_2, 298 K).

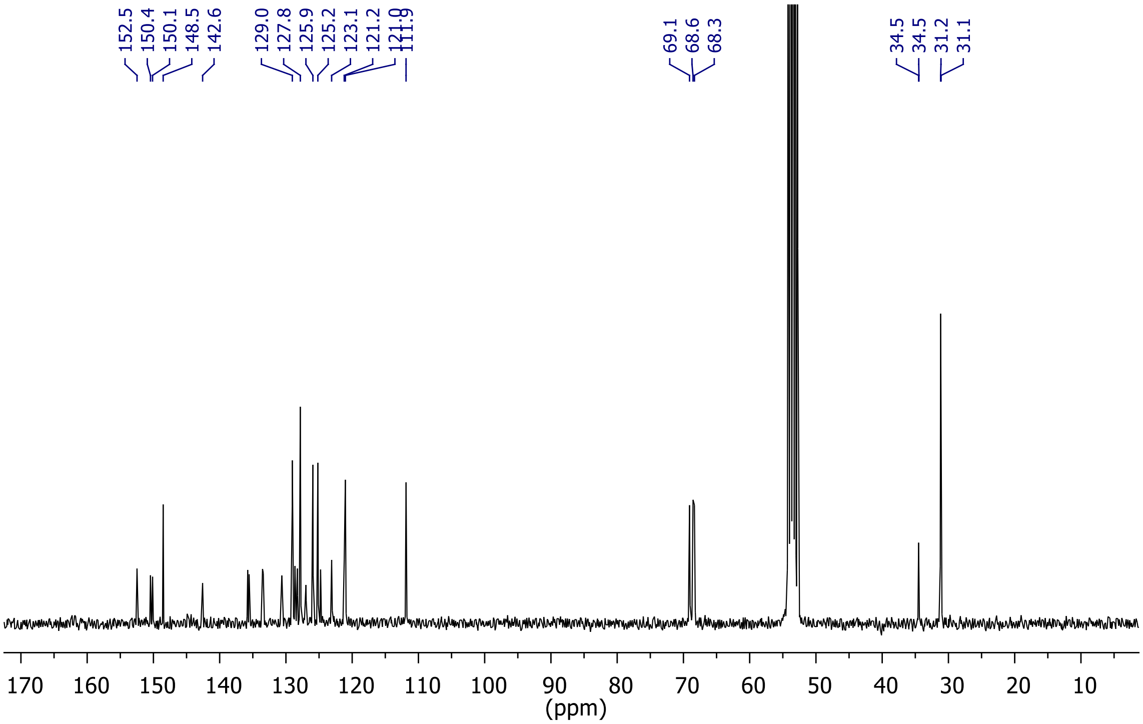

Figure S34. ^{13}C NMR spectrum of **R_5** (125 MHz, CD_3CN, 298 K).

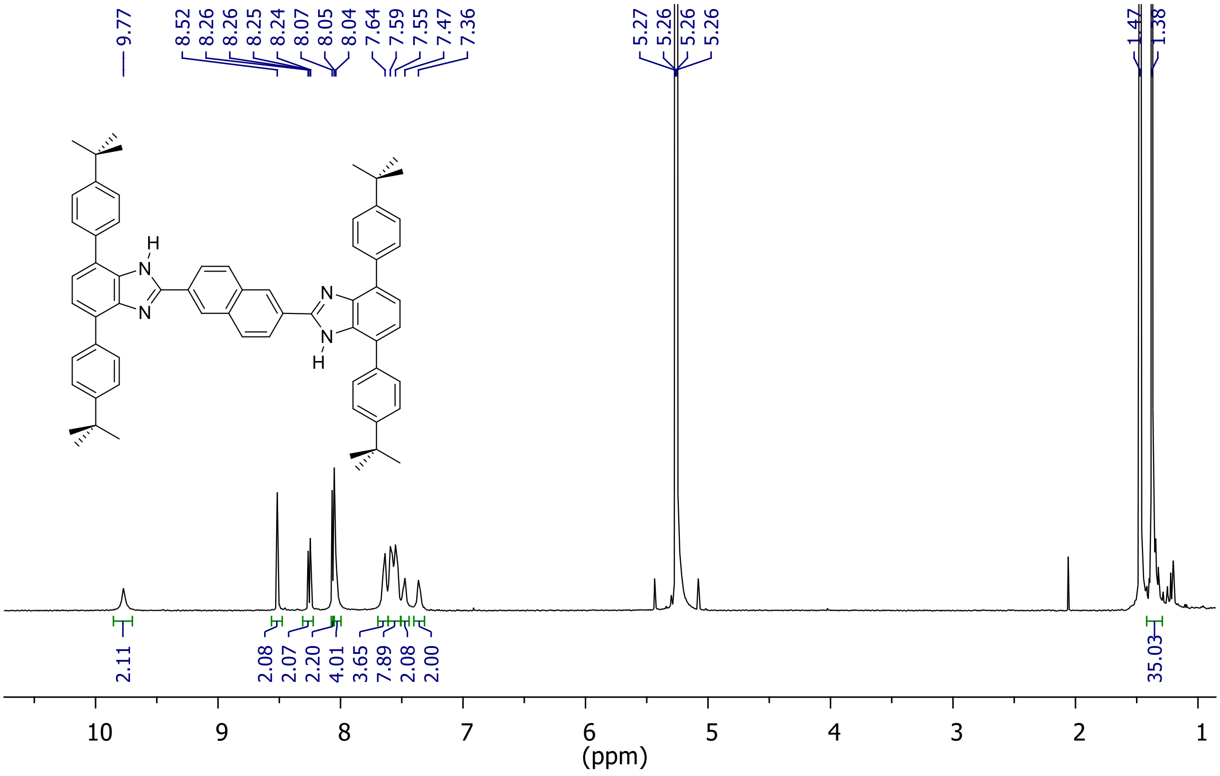

Figure S35. ^{1}H NMR spectrum of **R_5** (500 MHz, CD_2Cl_2, 298 K).

Synthesis of $[R_5\text{-}H_2][BF_4]_2$

Tetrafluoroboric acid diethyl ether complex (28 μL, 0.20 mmol) was added to a mixture of $\mathbf{R_5}$ (67 mg, 0.05 mmol) in dichloromethane (10 mL) with stirring. The solvent of the mixture was removed under vacuum. The residue was washed by diethyl ether and air dried. Yield: 70 mg, 92%. Mp> 300 °C. ^{1}H NMR (500 MHz, CD_3CN, 298 K) δ = 12.99 (br s, 2H), 12.46 (br s, 2H), 9.13 (s, 1H), 8.87 (s, 1H), 8.65 (d, 1H, J = 4.1 Hz), 8.44 (d, 1H, J = 4.1 Hz), 8.34 (d, 1H, J = 4.1 Hz), 8.21 (d, 1H, J = 4.1 Hz), 7.71-7.77 (m, 6H), 7.65 (d, 4H, J = 8.5 Hz), 7.44 (d, 4H, J = 8.5 Hz), 7.13 (s, 2H), 8.38 (d, 1H, J = 8.5 Hz), 8.23 (d, 1H, J = 8.5 Hz), 8.15 (d, 1H, J = 8.5 Hz), 8.08 (d, 1H, J = 8.5 Hz), 7.13 (s, 2H), 6.64 (m, 4H), 6.37 (m, 4H), 3.66-3.35 (m, 8H), 3.60 (m, 4H), 3.48 (m, 4H), 3.39 (m, 4H), 2.75 (m, 4H), 1.43 (s, 36H). ^{13}C NMR (125 MHz, CD_3CN, 298 K) δ = 152.31, 152.15, 150.85, 150.57, 147.34, 133.72, 133.53, 132.51, 132.47, 132.23, 131.64, 130.26, 130.06, 129.30, 129.23, 128.89, 128.83, 128.57, 127.69, 127.37, 126.53, 126.44, 126.13, 126.02, 125.92, 125.13, 121.30, 120.88, 112.55, 69.93, 69.82, 67.83, 34.51, 30.66, 30.64. HR-MS (ESI): calcd for $[M\text{-}BF_4]^+$, $[C_{44}H_{50}N_2O_4]^+$, m/z = 670.3765, found m/z = 670.3732.

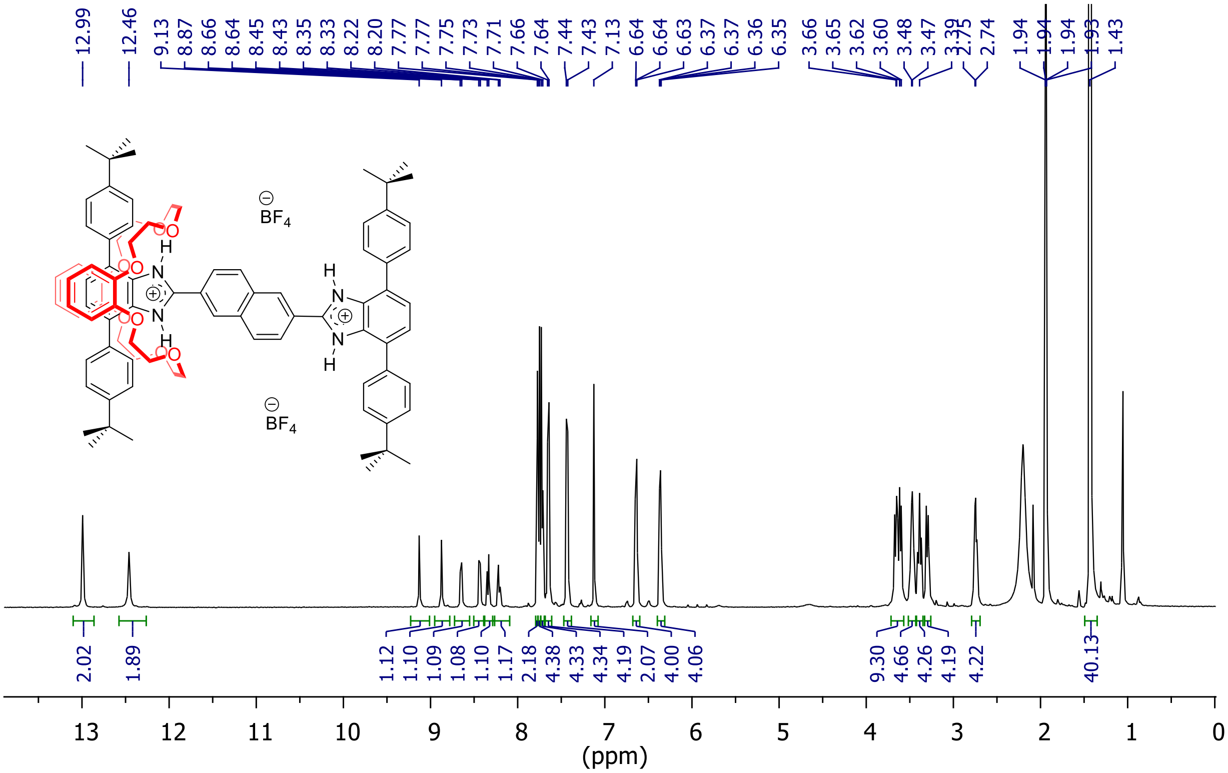

Figure S36. ^{1}H NMR spectrum of [**R_5**-H_2][BF_4]$_2$ (500 MHz, CD_3CN, 298 K).

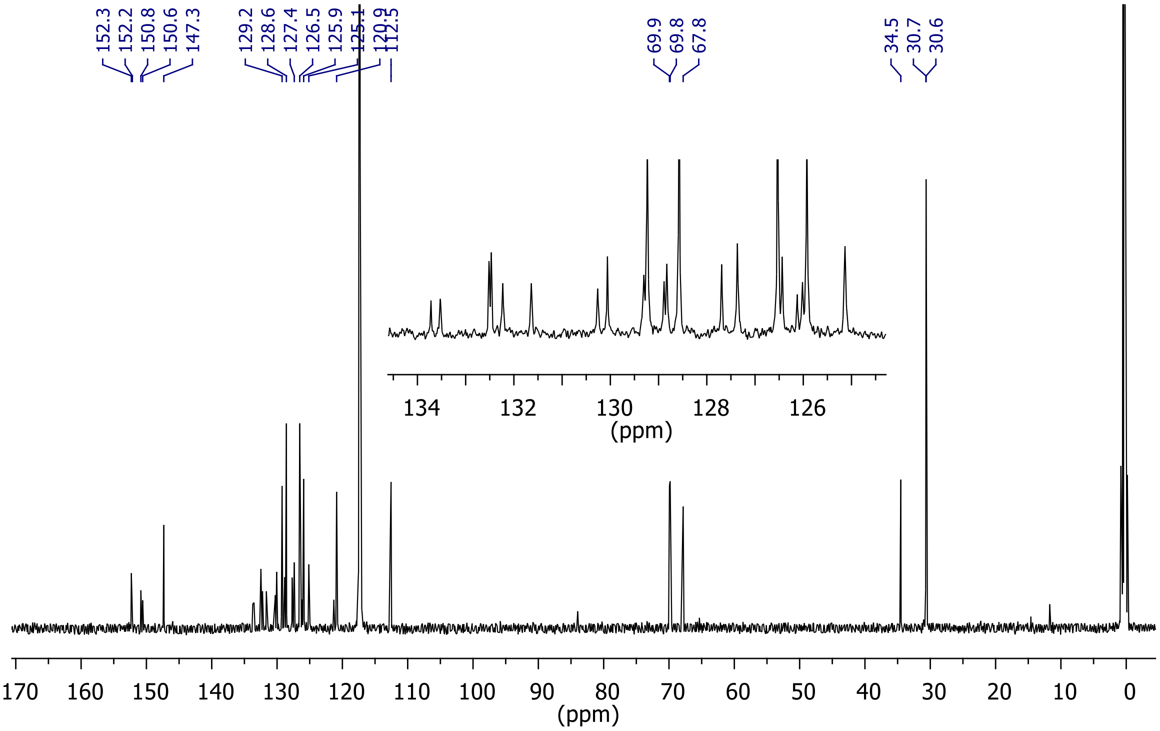

Figure S37. ^{13}C NMR spectrum of [**R_5**-H_2][BF_4]$_2$ (125 MHz, CD_3CN, 298 K).

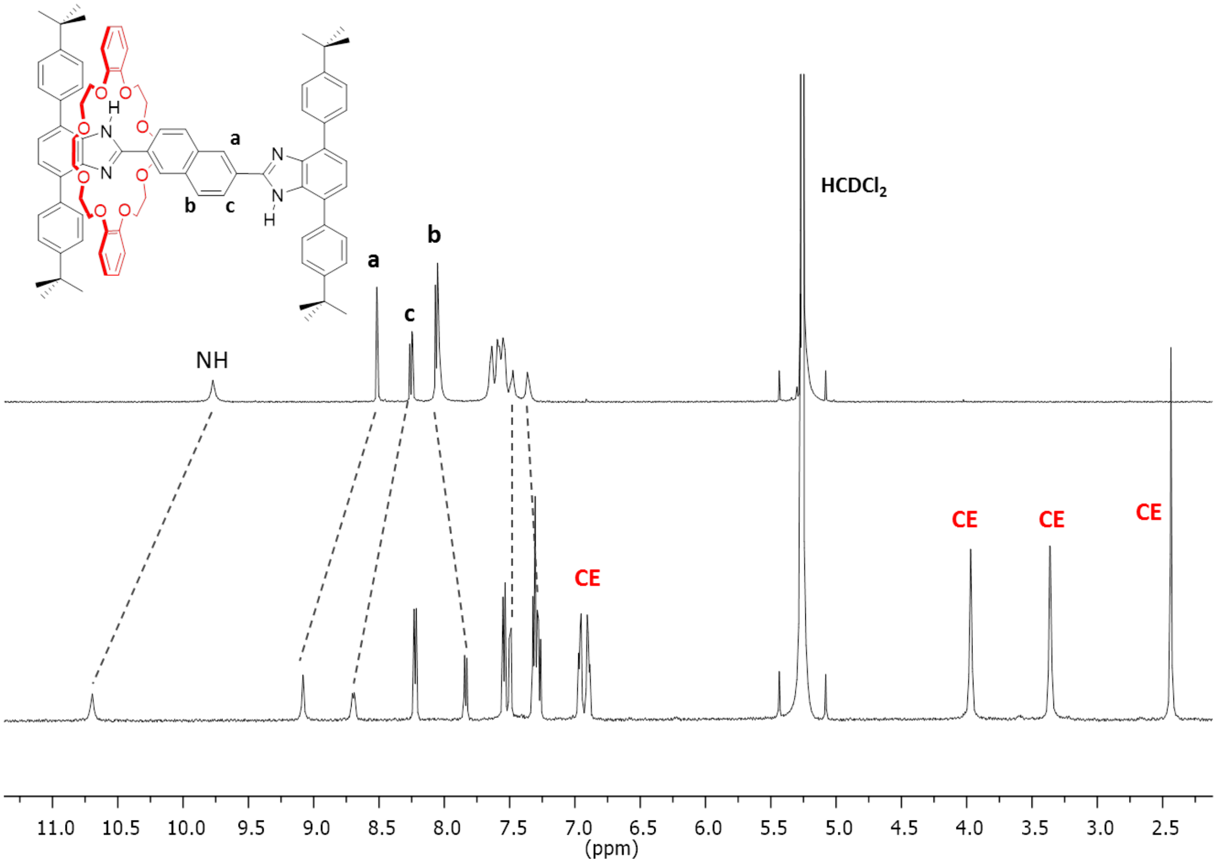

Figure S38. ^{1}H NMR (500 MHz, 298K, CD_2Cl_2); rotaxane (top) and dumbbell thread (bottom)

Determination of Shuttling Rates for Neutral [2]Rotaxane Molecular Shuttles R_2 – R_5

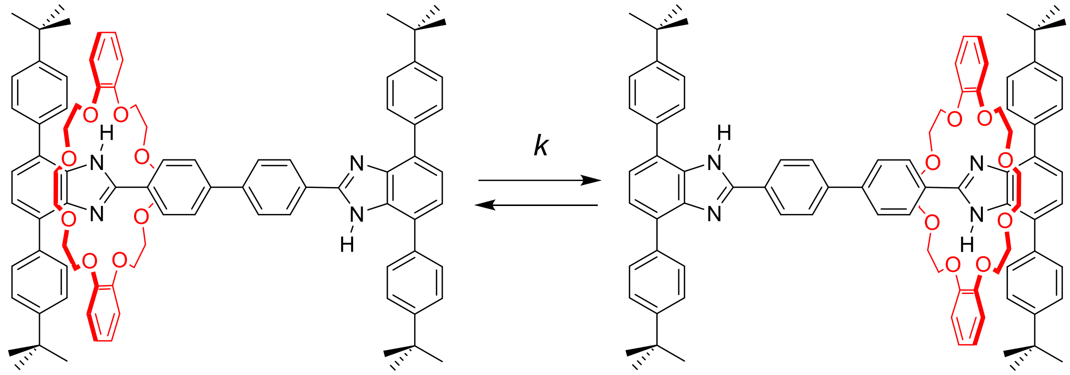

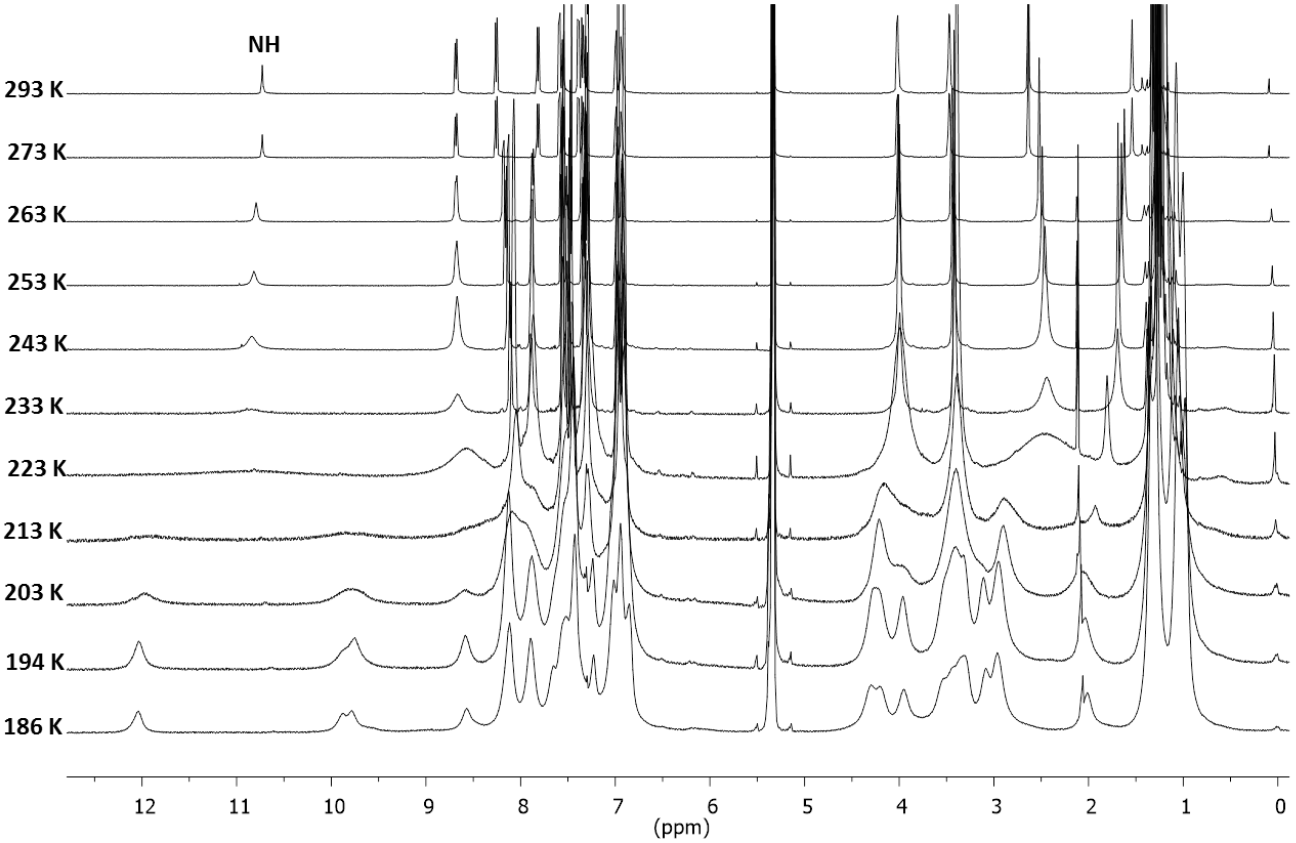

Figure S39. Variable temperature ^{1}H NMR of $\mathbf{R_2}$ (500 MHz, CD_2Cl_2)

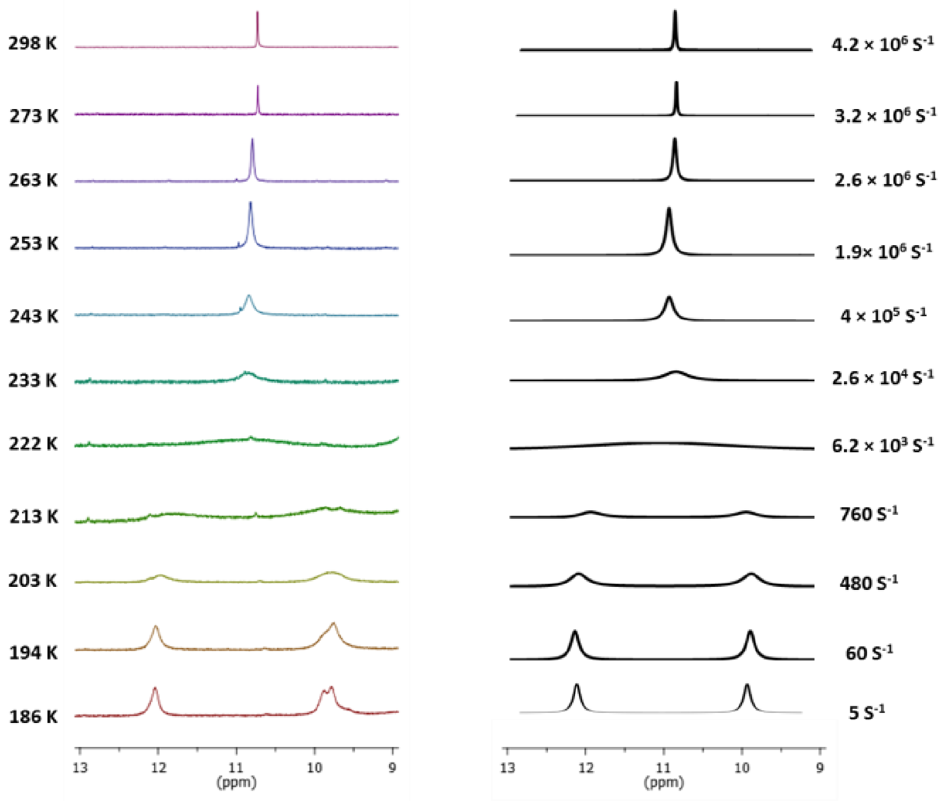

Figure S40. Comparison of the experimental and simulation ^{1}H NMR data

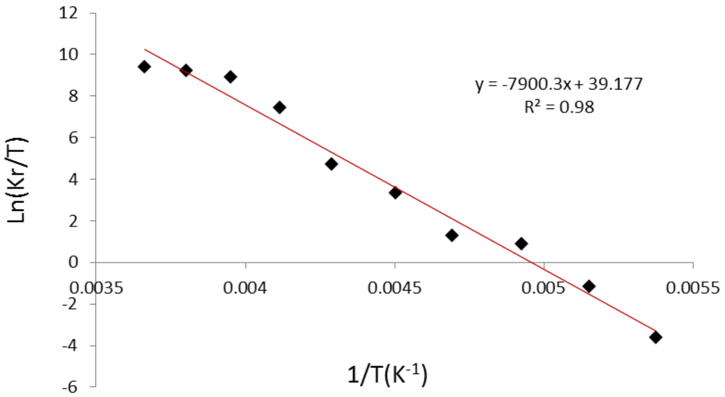

$\Delta H^{\ddagger}$ = 15.70 Kcal.mol^{-1}

$\Delta S^{\ddagger}$ = 30.64 cal.mol^{-1}.K^{-1}

Figure S41. Eyring plot generated from the simulated data for $\mathbf{R_2}$

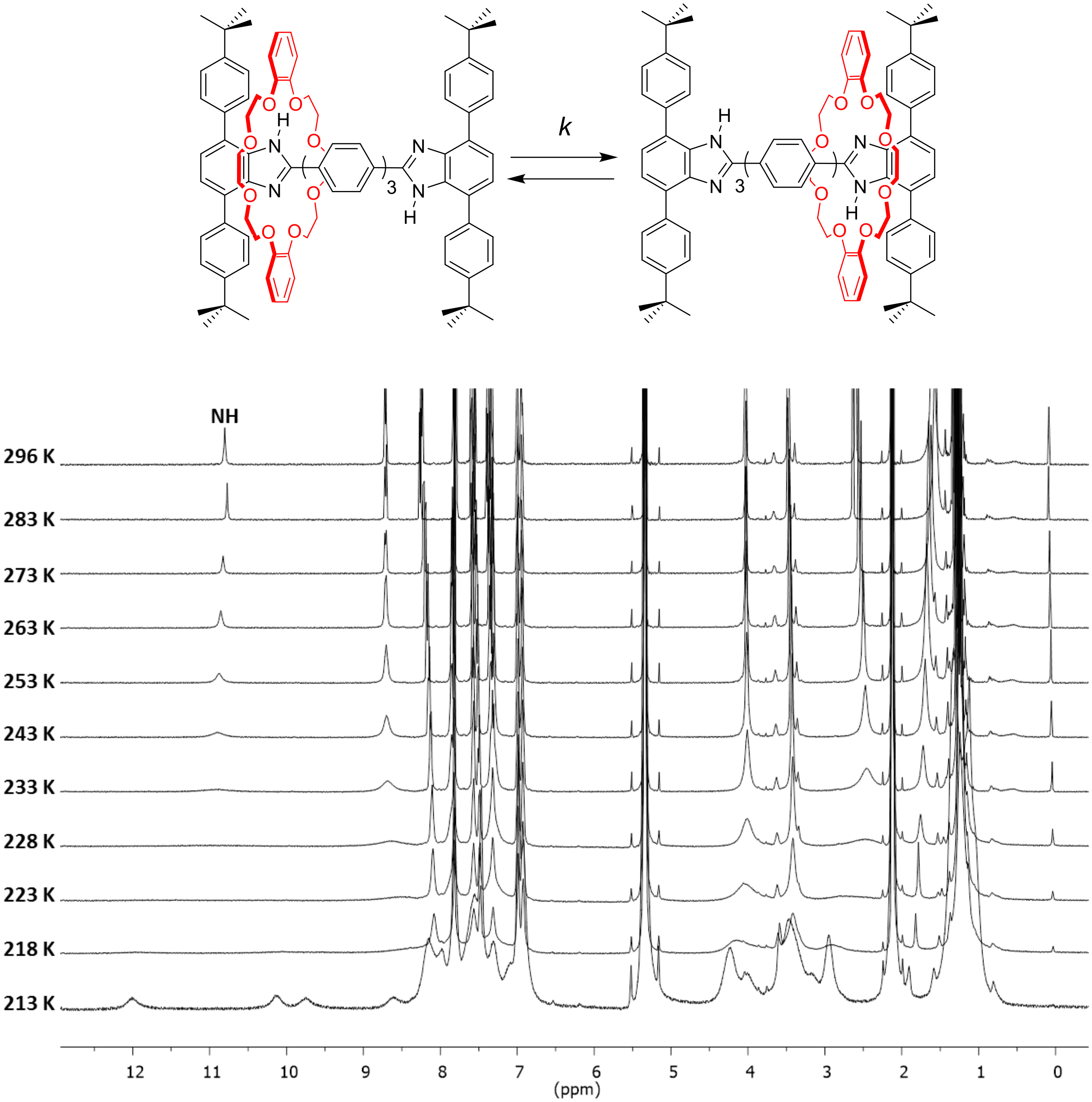

Figure S42. Variable temperature ^{1}H NMR of **R_3** (500 MHz, CD_2Cl_2)

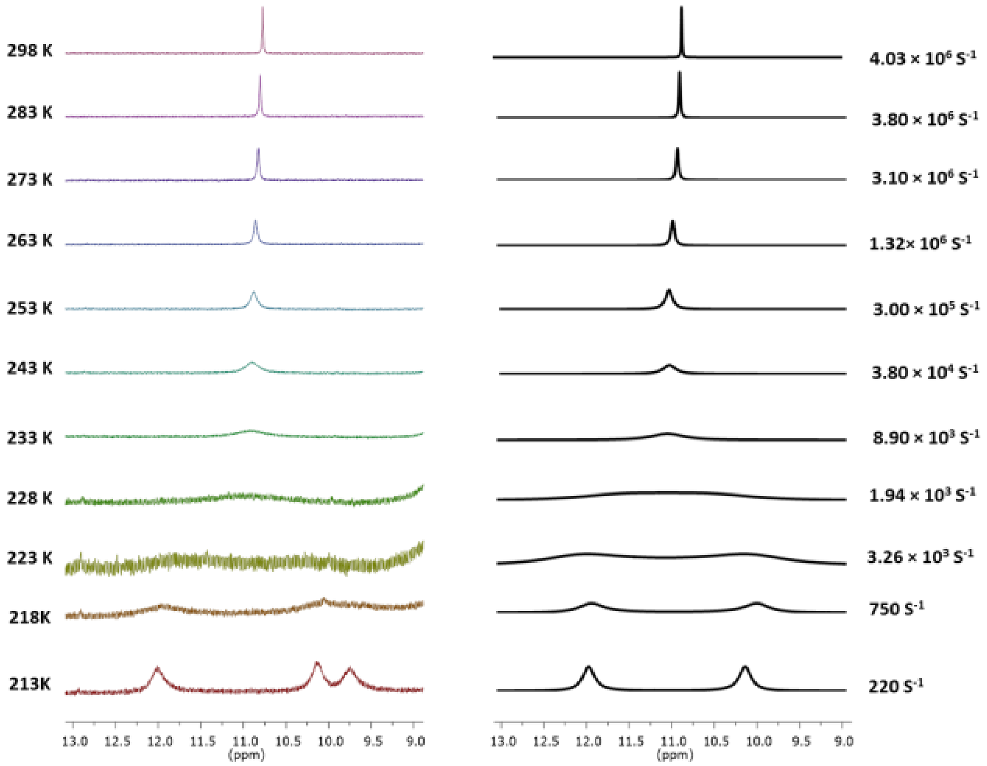

Figure S43. Comparison of the experimental and simulation ^{1}H NMR data

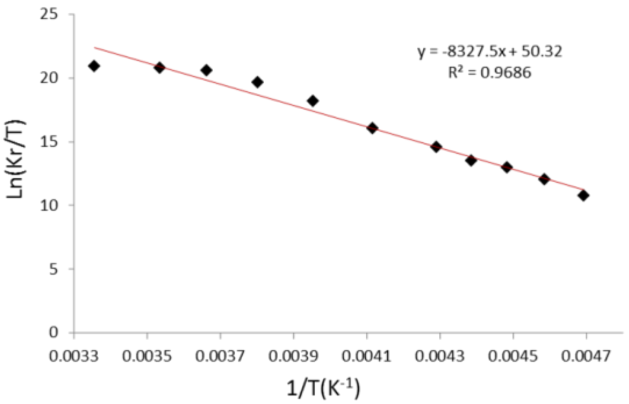

$\Delta H^{‡} = 16.55$ Kcal.mol^{-1}

$\Delta S^{‡} = 52.78$ cal.mol^{-1}.K^{-1}

Figure S44. Eyring plot generated from the simulated data (right) for **R_3**

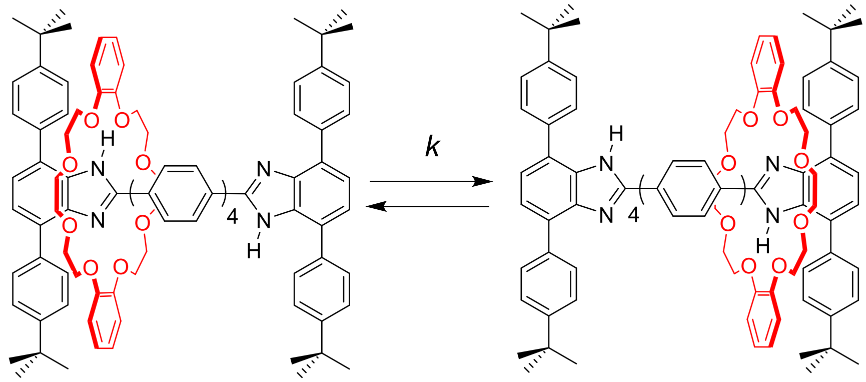

Figure S45. Variable temperature ^{1}H NMR of **R_4** (500 MHz, CD_2Cl_2)

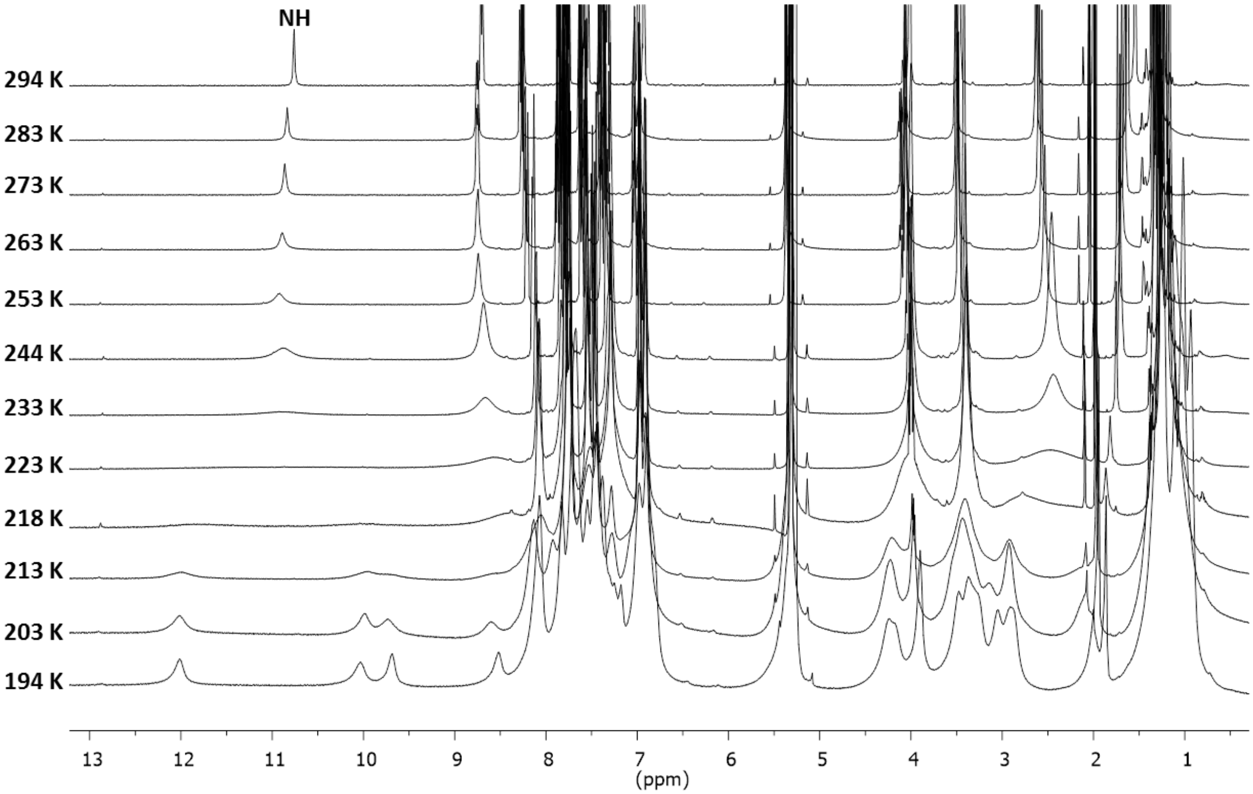

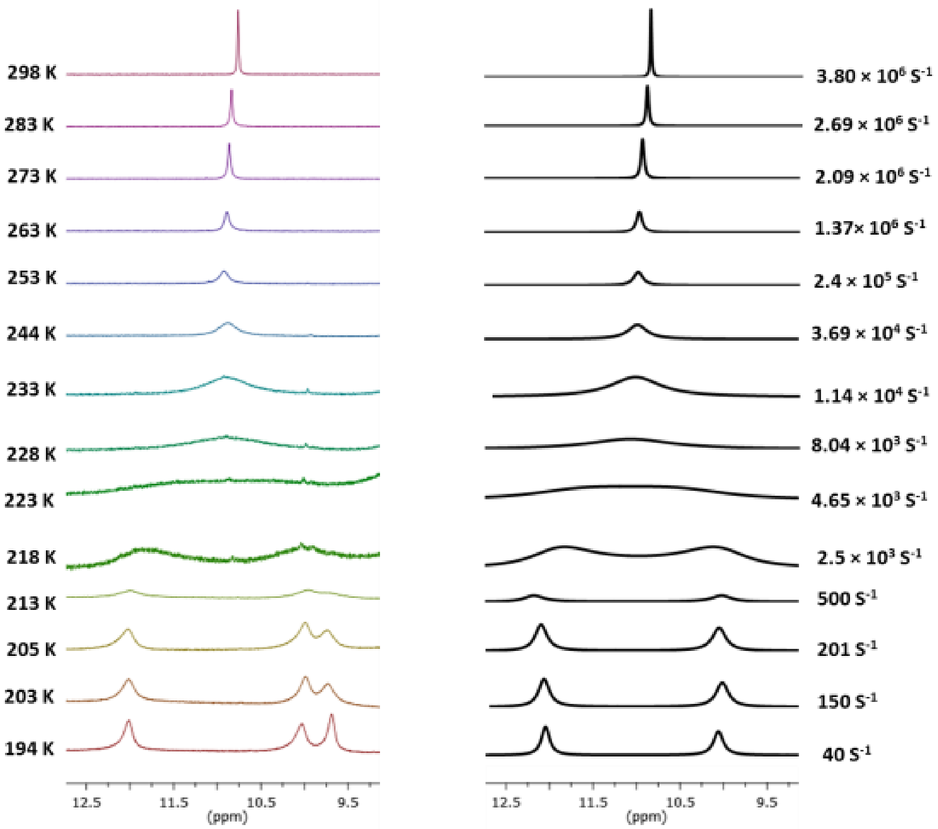

Figure S46. Comparison of the experimental and simulation ^{1}H NMR data (left)

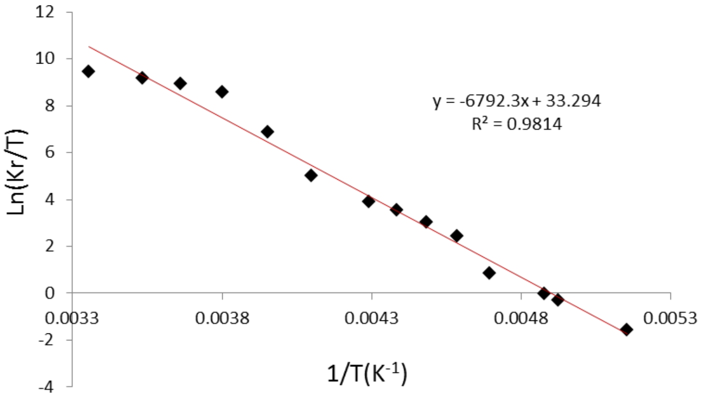

$\Delta H^{\ddagger}$ = 13.50 Kcal.mol^{-1}

$\Delta S^{\ddagger}$ = 18.95 cal.mol^{-1}.K^{-1}

Figure S47. Eyring plot generated from the simulated data (right) for $\mathbf{R_4}$

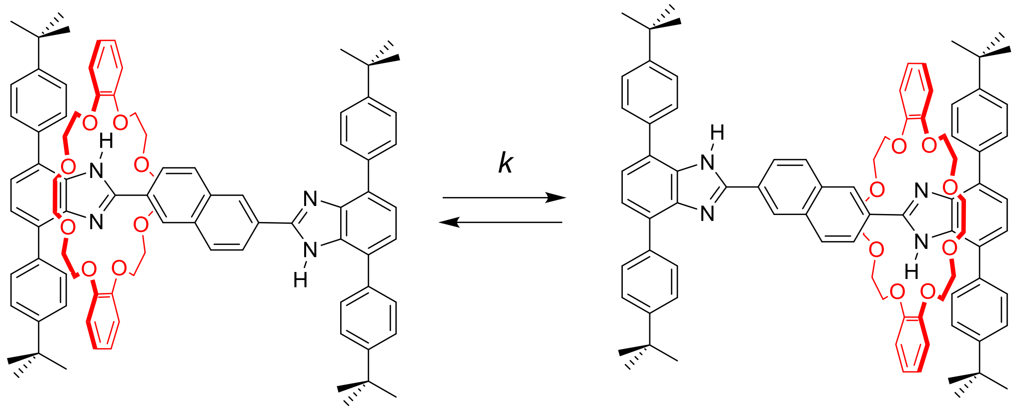

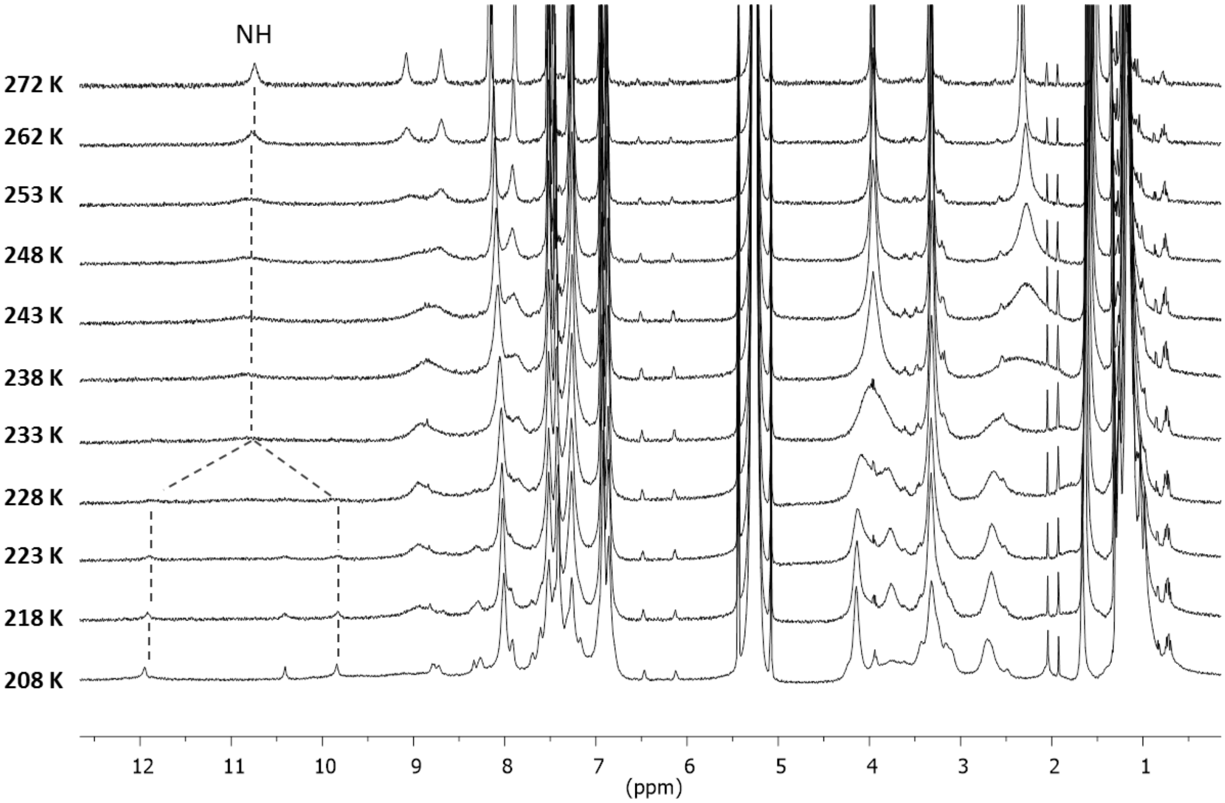

Figure S48. Variable temperature ^{1}H NMR of **R$_5$** (500 MHz, CD_2Cl_2)

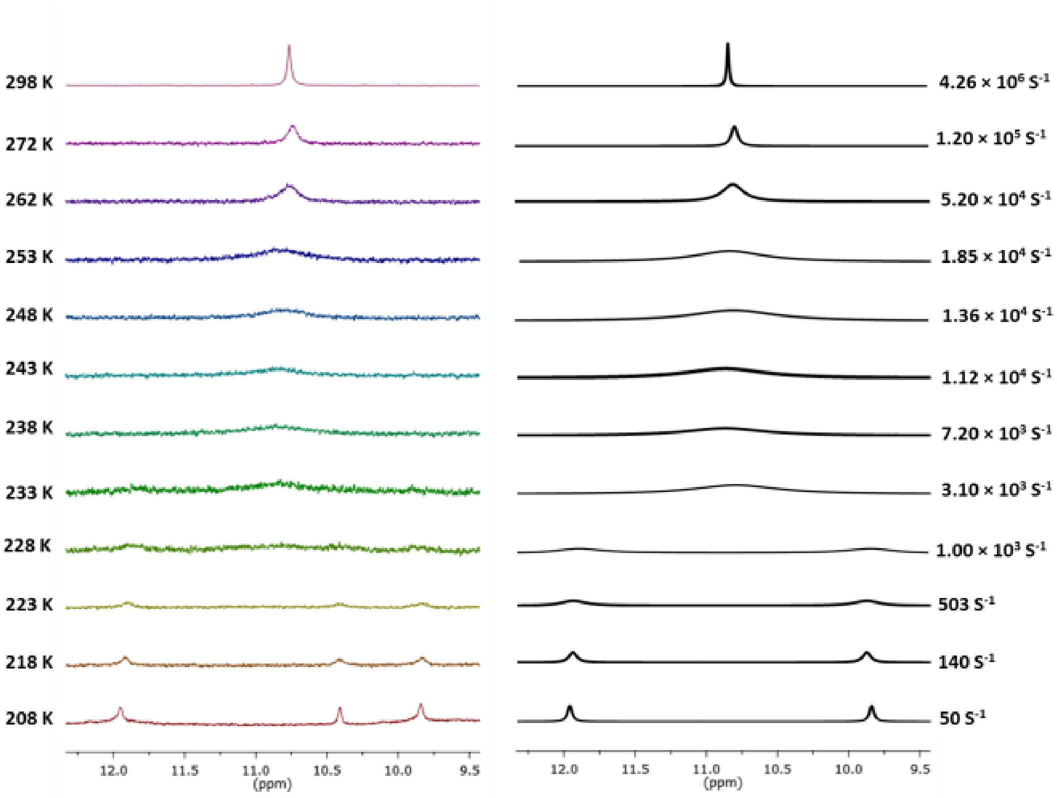

Figure S49. Comparison of the experimental and simulation ^{1}H NMR data

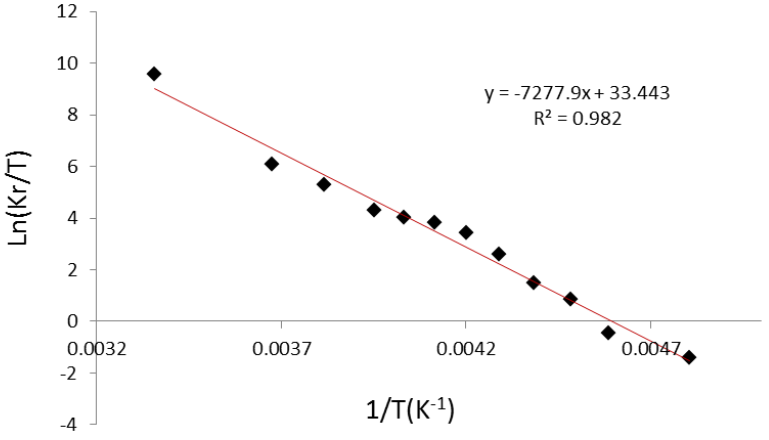

$\Delta H^{\ddagger}$ = 14.46 Kcal.mol^{-1}

$\Delta S^{\ddagger}$ = 19.24 cal.mol^{-1}.K^{-1}

Figure S50. Eyring plot generated from the simulated data (right) for **R$_5$**

The shuttling rates were calculated from a line shape analysis of the NMR spectra using the program DNMR71.EXE.[S6]

$$k_c = (\pi\Delta v)/2^{1/2}$$

k_c is the rate of shuttling at coalescence temperature. Δv is the limiting chemical shift (in Hz) between the exchanging proton resonances and can be determined by variable-temperature NMR. The Eyring equation,[S7] $\Delta G_c^{\neq} = -RT_c\ln(k_c h/k_B T_c)$, was used to estimate $\Delta G_c^{\neq}$ and extrapolate the rate of shuttling to other temperatures. T_c is the coalescence temperature, R is the gas constant, h is Planck's constant, and k_B is Boltzmann's constant.

The neutral species **R_1** could not be cooled down far enough to attain the limiting chemical shifts. We observed the coalescence temperature at 183 K, but nothing further. This data has been published[S8] and the VT spectra are available in the ESI of that article. By assuming the separation of the limiting peaks to be approximately 1000 Hz – this is about the average of the other (n = 2,3,4) samples – we roughly estimated an upper value for $\Delta G_c^{\neq}$ to be 7.73 kcal/mol.

Table S1 Summary of Shuttling Rates for Neutral [2]Rotaxane Molecular Shuttles

**shuttle**	Δv(Hz)	T_c(K)	k_c(s^{-1})	$k_{(298\ K)}$ (s^{-1})	$\boldsymbol{\Delta G_c^{\neq}}$(kcal/mol)	$\boldsymbol{\Delta G_{(298)}^{\neq}}$(kcal/mol)
**R_2**	1080	222	6200	4.20×10^6	9.01	8.41
**R_3**	932	228	3264	4.03×10^6	9.56	8.44
**R_4**	999	223	4650	3.80×10^6	9.18	8.47
**R_5**	609	233	3100	4.26×10^6	9.80	8.40

Determination of Shuttling Rates for Dicationic Molecular Shuttles $[R_1\text{-}H_2]^{2+}$ – $[R_5\text{-}H_2]^{2+}$

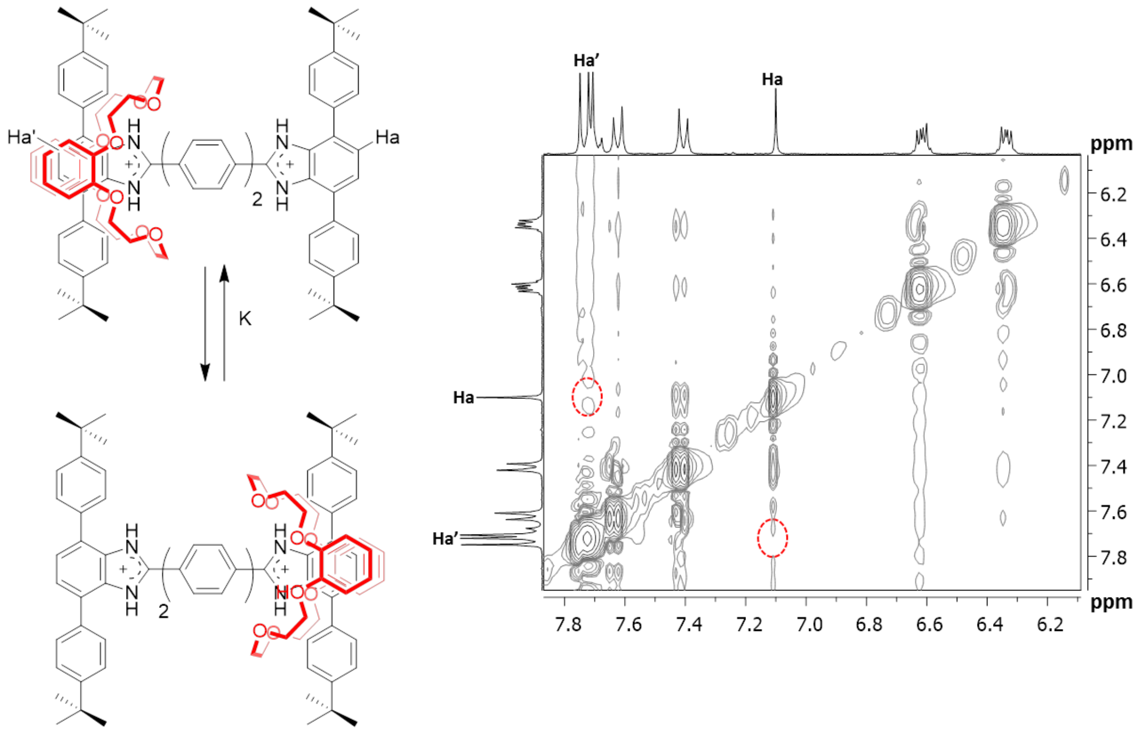

Figure S51. 2D ^{1}H-^{1}H EXSY (300 MHz, 295 K, CD_3CN) spectrum of [$\mathbf{R_2}$-H_2][BF_4]$_2$

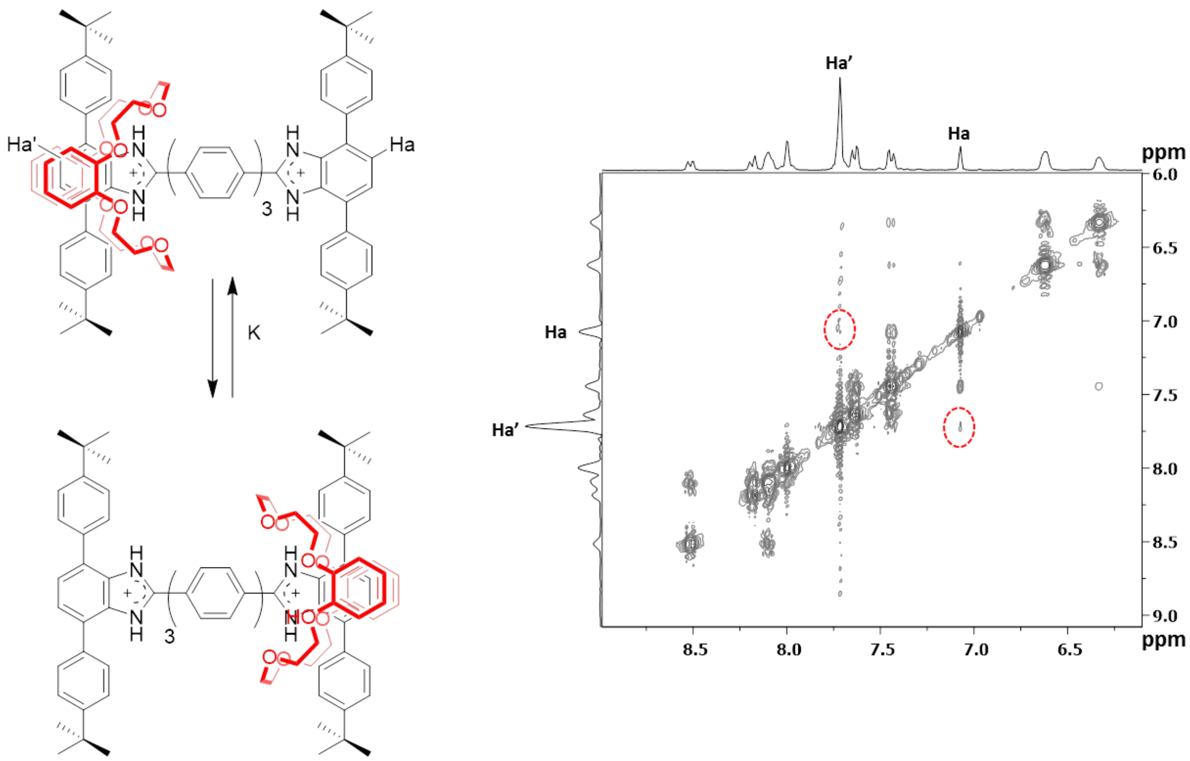

Figure S52. 2D ^{1}H-^{1}H EXSY (300 MHz, 295 K, CD_3CN) spectrum of [**R_3**-H_2][BF_4]$_2$

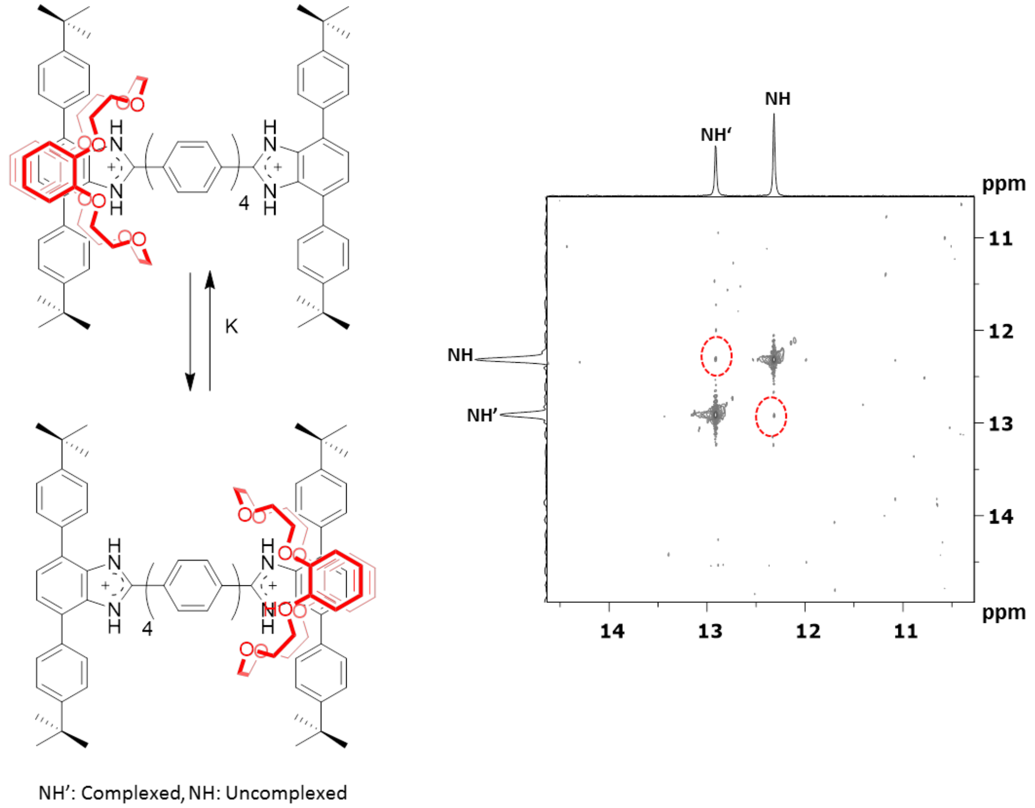

Figure S53. 2D ^1H-^1H EXSY (300 MHz, 293 K, CD_3CN) spectrum of [**R₄**-H_2][BF_4]$_2$

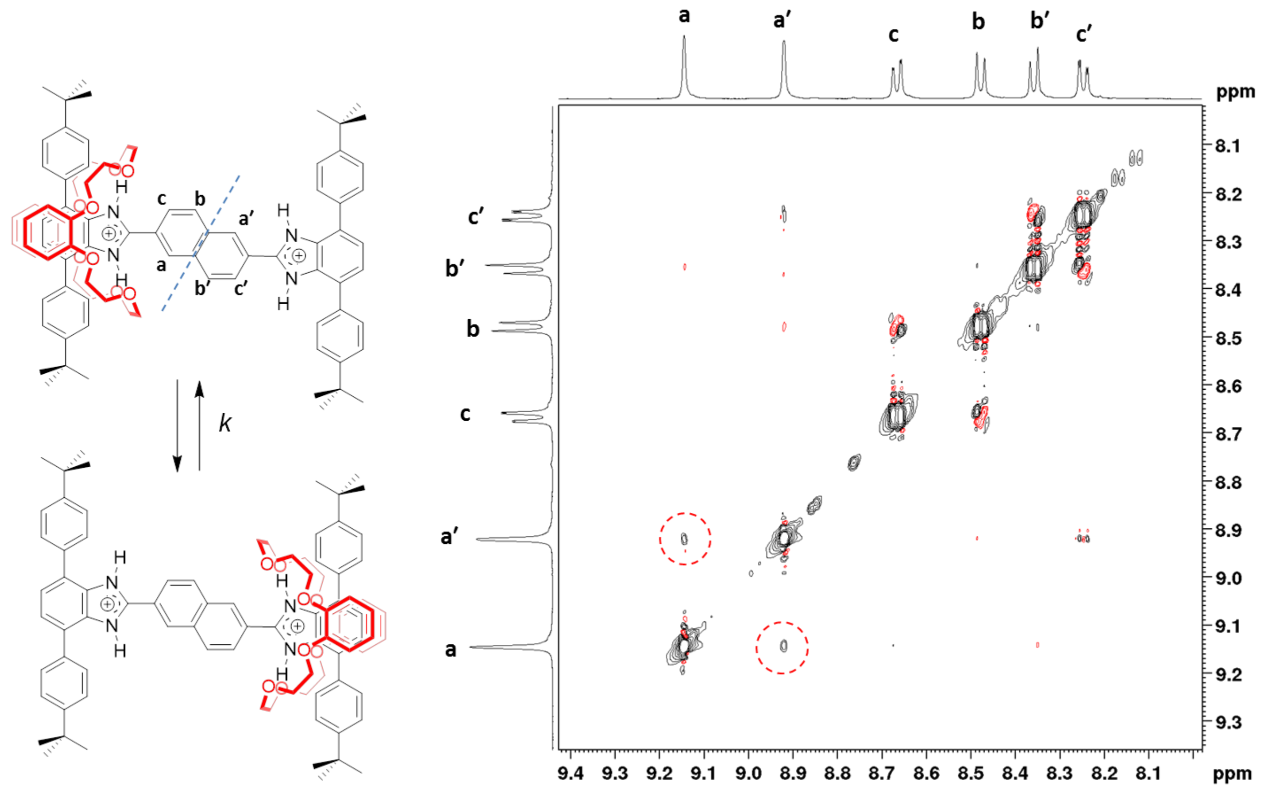

Figure S54. 2D ^{1}H-^{1}H EXSY (500 MHz, 298K, CD_3CN) spectrum of [**R5**-H_2][BF_4]$_2$

Table S2 Shuttling Rates for Dicationic [2]Rotaxane Molecular Shuttles

shuttle	I_{AA}	I_{BB}	I_{AB}	I_{BA}	k (s^{-1})	**Δ*G*≠** (kcal/mol)	**T** (K)
[**R2**-H_2][BF_4]$_2$	0.3421	0.0887	0.0013	0.00001	0.0061	20.21	295
[**R3**-H_2][BF_4]$_2$	1.7451	1.3902	0.0094	0.0033	0.0078	20.07	295
[**R4**-H_2][BF_4]$_2$	0.3385	0.3464	0.0016	0.0016	0.0090	19.83	293
[**R5**-H_2][BF_4]$_2$	0.9899	1.0000	0.0060	0.0063	0.0124	20.05	298

Details of Single X-ray Crystal Structure Determinations

X-ray Crystallography

General: Crystals were frozen in paratone oil inside a cryoloop under a cold stream of N_2. X-ray intensity data were collected at 173(2) K using a Bruker APEX diffractometer equipped with an APEX area detector. The raw area detector data frames were reduced and corrected for absorption effects using the SAINT+ and SADABS programs.[S9] Final unit cell parameters were determined by least-squares refinement taken from the data set. Diffraction data and unit-cell parameters were consistent with the assigned space groups. The structures were solved by direct methods with SHELXT.[S10] Subsequent difference Fourier calculations and full-matrix least-squares refinement against $|F^2|$ were performed with SHELXL-2014[S10] using OLEX2.[S11] All non-hydrogen atoms were refined anisotropically and hydrogen atoms placed in idealized positions and refined using a riding model. See Tables S1 and S2 for a summary of data collection, solution and refinement details. Complete details of the structures can be obtained from the Cambridge Crystallographic Data Centre at www.ccdc.cam.ac.uk for CCDC accession numbers 1563641, 1563642, 1563643 and 1563644.

X-ray structure of [2]Rotaxane R_5

Single crystals were obtained from slow evaporation of a DMF solution of $\mathbf{R_5}$. Crystals of formula $\mathbf{R_5}(DMF)_2$ were of good quality. Data was collected using MoKα radiation (λ = 0.71073 Å). The asymmetric unit contained one molecule of the [2]rotaxane ($C_{88}H_{96}N_4O_8$) and two molecules of DMF. The structure was solved in the triclinic space group P-1 (#2). One of the *t*Bu groups was disordered and modelled with occupancies of 81:19 using PART and FVAR. See Table S1 for details.

X-ray structure of [2]Rotaxane $[R_5\text{-}H_2][BF_4]_2$

Single crystals were obtained from slow evaporation of a toluene solution of $\mathbf{R_5}$-$H_2][BF_4]_2$. Crystals of formula $[\mathbf{R_5}\text{-}H_2][BF_4]_2(toluene)_4$ were of good quality. Data was collected using MoKα radiation (λ = 0.71073 Å). The asymmetric unit contained one molecule of the dicationic [2]rotaxane ($C_{88}H_{98}N_4O_8$), two BF_4 anions, three molecules of toluene and one water molecule. The structure was solved in the triclinic space group P-1 (#2). Two of the *t*Bu groups were disordered and both modelled with occupancies of 53:47 using PART and FVAR. Both anions were disordered and restrained with SAME & SIMU commands and modelled with occupancies of 84:16 and 79:21 respectively using PART and FVAR. The toluene molecules were included as rigid groups using a combination of SADI restraints. See Table S1 for details.

X-ray structure of [2]Rotaxane R_2 from THF

Single crystals were obtained from slow evaporation of a THF solution of **R_2**. Crystals of formula **R_2**(THF)$_4$ were of good quality. Data was collected using CuKα radiation (λ = 1.54178 Å). The asymmetric unit contained half a molecule of the [2]rotaxane ($C_{90}H_{98}N_4O_8$) and two molecules of THF. The structure was solved in the triclinic space group P-1 (#2). Both *t*Bu groups were disordered and modelled as rigid groups using SADI and SIMU with occupancies of 54:46 and 68:32 respectively using PART and FVAR. One of the THF molecules was disordered modelled with occupancies of 75:25 using PART and FVAR. See Table S1 for details.

X-ray structure of [2]Rotaxane R_1-Br

Single crystals were obtained from slow evaporation of an ethyl acetate solution of **R_1-Br**. Crystals of formula **R_1-Br** were of good quality. Data was collected using MoKα radiation (λ = 0.71073 Å). The asymmetric unit contained half a molecule of the [2]rotaxane ($C_{36}H_{42}Br_4N_4O_8$); no anions, no solvent. The structure was solved in the triclinic space group P-1 (#2). No restraints were required. See Table S1 for details.

Table S1. Single-crystal X-ray data collection, solution and refinement details for $\mathbf{R_5}$ and [$\mathbf{R_5}$-H_2][BF_4]$_2$.

CCDC No.	1563641	1563642
Compound	$\mathbf{R_5}$(DMF)$_2$	[$\mathbf{R_5}$-H_2][BF_4]$_2$(H_2O)(toluene)$_3$
Formula	$C_{94}H_{110}N_6O_{10}$	$C_{109}H_{124}B_2F_8N_4O_9$
M [gmol^{-1}]	1483.87	1807.73
Crystal system	Triclinic	Triclinic
Space group	P-1 (No. 2)	P-1 (No. 2)
a [Å]	14.3220(13)	14.2079(9)
b [Å]	15.9818(16)	18.9576(11)
c [Å]	20.0611(16)	20.1063(13)
α [°]	107.703(4)	97.249(3)
β [°]	90.106(4)	100.114(3)
γ [°]	110.238(4)	108.536(3)
V [Å^3]	4074.8(7)	4957.8(5)
Z	2	2
D_{calcd}. [gcm^{-3}]	1.209	1.211
μ[mm^{-1}]	0.078	0.086
Reflections	8510	20185
R_{int}	0.1148	0.0518
Parameters	1007	1400
Restraints	114	328
R1 [I > 2σ(I)]^a	0.1041	0.1057
R1 (all data)	0.2001	0.1744
wR2 [I > 2σ(I)]^b	0.2151	0.2079
wR2 (all data)	0.2713	0.2555
GoF (F^2)	1.026	1.106
$\Delta\rho$ [e Å^{-3}]	+0.39 (-0.41)	+0.63 (-0.63)

^{a}R1 = $\Sigma \,||F_o| - |F_c|| \,/\, \Sigma\, |F_o|$; R2$w$ = $[\Sigma[w(F_o^2 - F_c^2)^2] \,/\, \Sigma[w(F_o^2)^2]]^{1/2}$
^{b}$w = q[\sigma^2(F_o^2) + (aP)^2 + bP]^{-1}$.

Table S2. Single-crystal X-ray data collection, solution and refinement details for $\mathbf{R_2}$ and $\mathbf{R_1}$**-Br**.

CCDC No.	1563643	1563644
Compound	$\mathbf{R_2}(THF)_4$	$\mathbf{R_1}$**-Br**
Formula	$C_{106}H_{130}N_4O_{12}$	$C_{36}H_{42}Br_4N_4O_8$
M [$gmol^{-1}$]	1652.13	978.37
Crystal system	Triclinic	Triclinic
Space group	P-1 (No. 2)	P-1 (No. 2)
a [Å]	12.0595(2)	9.6346(14)
b [Å]	12.5637(3)	10.1658(15)
c [Å]	16.8685(3)	11.2070(17)
α [°]	74.7825(11)	68.379(2)
β [°]	76.6602(12)	82.740(2)
γ [°]	70.7332(11)	68.992(2)
V [$Å^3$]	2298.72(8)	10682(13)
Z	1	1
$D_{calcd.}$ [gcm^{-3}]	1.193	1.706
μ[mm^{-1}]	0.607	4.281
Reflections	8028	3839
R_{int}	0.0557	0.0518
Parameters	664	235
Restraints	100	0
$R1$ [$I > 2\sigma(I)$][a]	0.0584	0.0374
$R1$ (all data)	0.0962	0.0438
$wR2$ [$I > 2\sigma(I)$][b]	0.1379	0.0995
$wR2$ (all data)	0.1570	0.1042
GoF (F^2)	1.037	1.032
$\Delta\rho$ [e $Å^{-3}$]	+0.36 (-0.23)	+0.99 (-0.36)

[a] $R1 = \Sigma\,||F_o| - |F_c||\,/\,\Sigma\,|F_o|$; $R2w = [\Sigma[w(F_o^2 - F_c^2)^2]\,/\,\Sigma[w(F_o^2)^2]]^{1/2}$
[b] $w = q[\sigma^2(F_o^2) + (aP)^2 + bP]^{-1}$.

Methods and Computational Details

Quantum mechanics calculations were carried out in the framework of the Density Functional Theory (DFT) methods incorporated in the GAUSSIAN09 package.[S12] The ground state equilibrium structures and the transition state (TS) structures of the dibenzo[24]crown-8 (**DB24C8**) macrocycle and an H-shaped axle, forming a rotaxane, were fully optimized without symmetry restraints. The track length of the axle was varied in terms of the amount (n) of phenylene spacers, increasing from n=1 to n=4. The Becke-3-parameter-Lee-Yang–Parr hybrid functional (B3LYP),[S13] which incorporates 20% of Hartree-Fock exact exchange, was employed as it has been reported to achieve good agreement with X-ray geometrical structures of even large molecular systems.[S14] The Gaussian-type basis set 6-31G(d,p)[S15] was employed for all atoms and the dispersion correction (DFT-D3) to the energy was included in all the calculations using the Grimme scheme.[S16]

Molecular structures of the starting and finishing points for the shuttling of the rotaxane, which were constructed with the **DB24C8** macrocycle in each extreme of the axle, were optimized to find stationary point geometries. The structures of the TS between these geometries were optimized by applying Schlegel's synchronous-transit-guided quasi-Newton (QST3) method.[S17] The nature of the found stationary points on the potential energy surface (PES) was characterized by the computation of the harmonic vibrational frequencies obtained by the examination of the Hessian matrix, which is constituted by the second derivative of energy with respect to the spatial coordinates of the systems. The transition states were verified to be first order saddle points with only one negative eigenvalue. Implicit solvation effects were taken into account by means of the Polarized Continuum Model (PCM) using dichloromethane (DCM, ε = 8.93).[S18]

Table S3. Calculated thermochemistry values and energy barriers for $\mathbf{R_1}$ – $\mathbf{R_4}$ and $[\mathbf{R_1}\text{-}H_2]^{2+}$ – $[\mathbf{R_4}\text{-}H_2]^{2+}$.

n	Charge	Start	TS	Finish	$\Delta G^{\neq}$ (AU)$_{Th}$	$\Delta G^{\neq}$ (kcal mol^{-1})$_{Th}$
1	0	-4077.19	-4077.17	-4077.19	0.018	11.1
2	0	-4308.18	-4308.15	-4308.18	0.027	17.1
3	0	-4539.17	-4539.14	-4539.17	0.033	20.5
4	0	-4770.17	-4770.14	-4770.17	0.033	20.9
1	+2	-4078.08	-4078.05	-4078.07	0.029	18.0
2	+2	-4309.08	-4309.02	-4309.07	0.054	34.1
3	+2	-4540.08	-4540.01	-4540.07	0.068	42.6
4	+2	-4771.07	-4771.00	-4771.06	0.074	46.4

R_n	TS Structure (Neutral)	TS Structure (Charged)
n =1	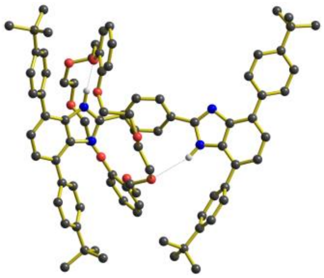	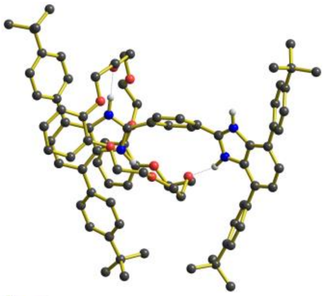
n = 2	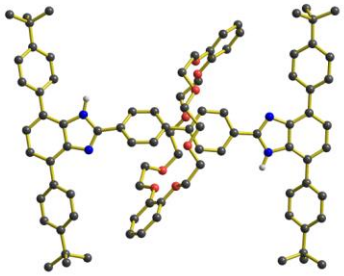	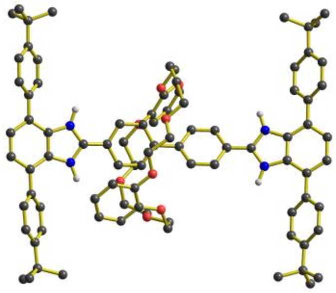
n = 3	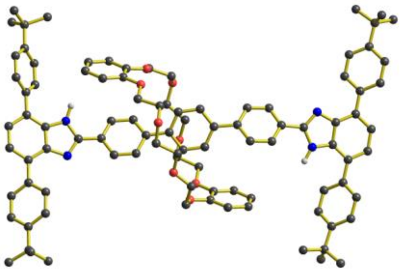	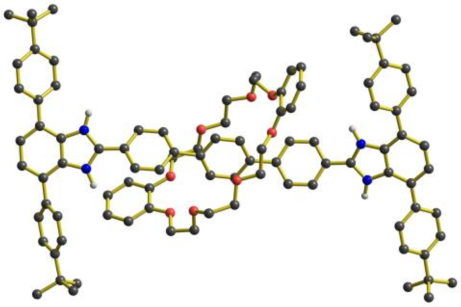
n = 4	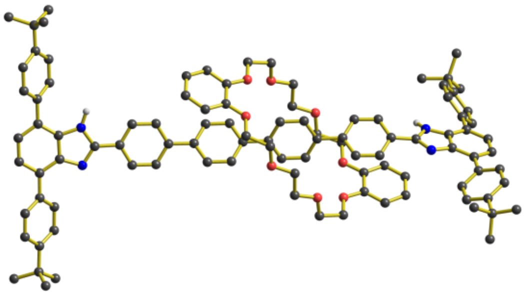	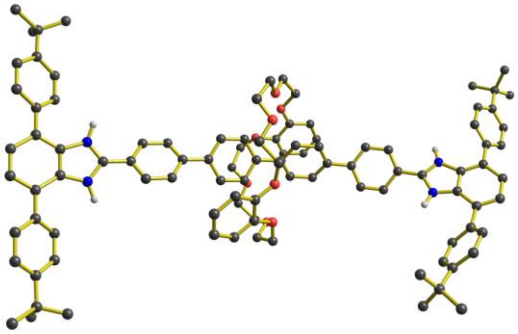

Figure S55. Calculated TS structures for $\mathbf{R_1}$ – $\mathbf{R_4}$ and $[\mathbf{R_1}\text{-}H_2]^{2+}$ – $[\mathbf{R_4}\text{-}H_2]^{2+}$.

References

S1 H. Akpinar, A. Balan, D. Baran, E. Unver, L. Toppare, *Polymer,* **2010**, 51, 6123–6131.

S2 K. Zhu, V. N. Vukotic, N. Noujeim, S. J. Loeb, *Chem. Sci.,* **2012,** 3*,* 3265–3271.

S3 M. Kozakova, M. Budesinsky, J. Hodacova, *Synthetic Communication*, **2005**, *35*, 161–167.

S4 H. C.hun Cheng, P. P. Yu Chen, Y. Oliver Su, *Dalton Trans*., **2014**, *43*, 1424–1433.

S5 G.-Y. Xie, L. Jiang, T.-B. Lu, *Dalton Trans*., **2013**, *42*, 14092–14099.

S6 DNMR71.EXE, Reich, H. J., *J. Chem. Educ. Software*, 1996.

S7 I. O. Sutherland, *Annu. Rep. NMR Spectrosc*. **1971**, *4*, 71.

S8 K. Zhu, V. N. Vukotic, N. Noujeim, S. J. Loeb, *Chem. Sci*. 2012, **3**, 3265.

S9 Bruker, SAINT+ and SADABS. Bruker AXS Inc., Madison, Wisconsin, USA, 2012.

S10 G. M. Sheldrick, *Acta Cryst*. **2015**, *C71*, 3–8.

S11 O. V. Dolomanov, L. J. Bourhis, R. J. Gildea, J. A. K. Howard, H. Puschmann*, J. Appl. Cryst*., **2009**, *42*, 339–341.

S12 M. J. Frisch, G. W. Trucks, H. B. Schlegel, G. E. Scuseria, M. A. Robb, J. R. Cheeseman, G. Scalmani, V. Barone, B. Mennucci, G. A. Petersson, H. Nakatsuji, M. Caricato, X. Li, H. P. Hratchian, A. F. Izmaylov, J. Bloino, G. Zheng, J. L. Sonnenberg, M. Hada, M. Ehara, K. Toyota, R. Fukuda, J. Hasegawa, M. Ishida, T. Nakajima, Y. Honda, O. Kitao, H. Nakai, T. Vreven, J. A. Montgomery, Jr., J. E. Peralta, F. Ogliaro, M. Bearpark, J. J. Heyd, E. Brothers, K. N. Kudin, V. N. Staroverov, R. Kobayashi, J. Normand, K. Raghavachari, A. Rendell, J. C. Burant, S. S. Iyengar, J. Tomasi, M. Cossi, N. Rega, J. M. Millam, M. Klene, J. E. Knox, J. B. Cross, V. Bakken, C. Adamo, J. Jaramillo, R. Gomperts, R. E. Stratmann, O. Yazyev, A. J. Austin, R. Cammi, C. Pomelli, J. W. Ochterski, R. L. Martin, K. Morokuma, V. G. Zakrzewski, G. A. Voth, P. Salvador, J. J. Dannenberg, S. Dapprich, A. D. Daniels, O¨. Farkas, J. B. Foresman, J. V. Ortiz and J. Cioslowski and D. J. Fox, *Gaussian 09, Revision E.01, Gaussian, Inc.*, Wallingford CT, 2009.

S13 (a) A. D. Becke, J. Chem. Phys. 1993, **98**, 5648; (b) P. J. Stephens, F. J. Devlin, C. F. Chabalowski, M. J. Frisch, *J. Phys. Chem*. 1994, **98**, 11623.

S14 (a) S. Tortorella, M. M. Talamo, A. Cardone, M. Pastore, F. De Angelis. *J. Phys.: Condens. Matter*. 2016, **28**, 074005 (11 pp); (b) M Pastore, E Mosconi, F De Angelis, M. Grätzel. *J. Phys. Chem. C.* 2010, **114**, 7205; (c) M. N. Glukhovtsev, R. D. Bach, C. J. Nagel. *J. Phys. Chem. A*. 1997, **101**, 316.

S15 M. J. Frisch, J. A. Pople, J. S. Binkley. *J. Chem. Phys*. 1984, **80**, 3265.

S16 S. Grimme, J. Antony, S. Ehrlich, H. Krieg, *J. Chem. Phys*. 2010, **132**, 1541041 (19 pp).

S17 C. Peng, H. B. Schlegel. *Israel J. Chem*. 1993, **33**, 449.

S18 M. Cossi, N. Rega, G. Scalmani and V. Barone, *J. Comput. Chem.*, 2003, **24**, 669.
